# Recent Trends in the Quantification of Biogenic Amines in Biofluids as Biomarkers of Various Disorders: A Review

**DOI:** 10.3390/jcm8050640

**Published:** 2019-05-09

**Authors:** Alina Plenis, Ilona Olędzka, Piotr Kowalski, Natalia Miękus, Tomasz Bączek

**Affiliations:** 1 Department of Pharmaceutical Chemistry, Medical University of Gdańsk, Hallera 107, 80-416 Gdańsk, Poland; ilona@gumed.edu.pl (I.O.); piotr.kowalski@gumed.edu.pl (P.K.); miekusn@gmail.com (N.M.); tbaczek@gumed.edu.pl (T.B.); 2 Department of Animal and Human Physiology, Faculty of Biology, University of Gdańsk, Wita Stwosza 59, 80-308 Gdańsk, Poland

**Keywords:** biogenic amines, biomarkers, human samples, liquid chromatography, gas chromatography, capillary electrophoresis, non-separation techniques

## Abstract

Biogenic amines (BAs) are bioactive endogenous compounds which play a significant physiological role in many cell processes like cell proliferation and differentiation, signal transduction and membrane stability. Likewise, they are important in the regulation of body temperature, the increase/decrease of blood pressure or intake of nutrition, as well as in the synthesis of nucleic acids and proteins, hormones and alkaloids. Additionally, it was confirmed that these compounds can be considered as useful biomarkers for the diagnosis, therapy and prognosis of several neuroendocrine and cardiovascular disorders, including neuroendocrine tumours (NET), schizophrenia and Parkinson’s Disease. Due to the fact that BAs are chemically unstable, light-sensitive and possess a high tendency for spontaneous oxidation and decomposition at high pH values, their determination is a real challenge. Moreover, their concentrations in biological matrices are extremely low. These issues make the measurement of BA levels in biological matrices problematic and the application of reliable bioanalytical methods for the extraction and determination of these molecules is needed. This article presents an overview of the most recent trends in the quantification of BAs in human samples with a special focus on liquid chromatography (LC), gas chromatography (GC) and capillary electrophoresis (CE) techniques. Thus, new approaches and technical possibilities applied in these methodologies for the assessment of BA profiles in human samples and the priorities for future research are reported and critically discussed. Moreover, the most important applications of LC, GC and CE in pharmacology, psychology, oncology and clinical endocrinology in the area of the analysis of BAs for the diagnosis, follow-up and monitoring of the therapy of various health disorders are presented and critically evaluated.

## 1. Introduction

The unbalanced secretion and elimination of pivotal biogenic amines (BAs) (such as serotonin (5-hydroxytryptamine, 5-HT), dopamine (DA), norepinephrine (NE) and epinephrine (E)) can have severe health consequences since they act not only as neurotransmitters but take part in the regulation of mood, sleep, immune response, thermoregulation and cardiovascular and gut functions, among others [1]. To start with, in the diagnosis and monitoring of central nervous system (CNS) pathologies, such as neurodegenerative disorders, levels of BAs and their main metabolites in body fluids differentiated the healthy persons from the patients with Alzheimer’s Disease (AD) [2]. Also, changes in the central noradrenergic, serotonergic and dopaminergic neurotransmitter systems were demonstrated to play a relevant role in the behavioural and psychological signs and symptoms of dementia manifestation [3]. Other studies revealed that not only must dopaminergic transmission be monitored for Parkinson’s Disease (PD) but also non-DA neurotransmitter systems in the brain are significantly desynchronized in PD, as well as the interaction between the different BA systems, which contributes to the development of the manifestations of PD [4]. Neuroendocrine system pathologies might be diagnosed and monitored through the determination of BAs, for example: the diagnosis of catecholamine-producing neuroendocrine tumours (NETs): pheochromocytoma (PHE) and neuroblastoma (NB), is supported by the determination of BAs in patients’ body fluids (urine, plasma, serum) and indoleamine-producing NETs–carcinoids–could be diagnosed via discriminating the level of 5-hydroxyindoleacetic acid (5-HIAA)–the main serotonin metabolite–in patients’ 24-hour urine samples [5].

**Figure 1:**
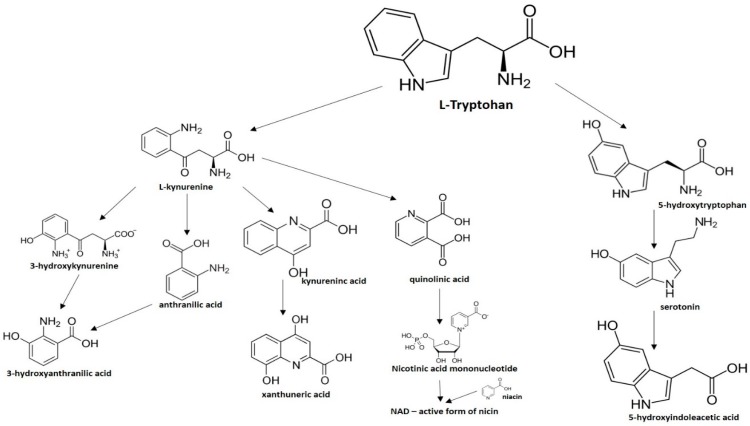
The main products of the L-Tryp metabolic pathway.

From the L-Tryptophan (L-Tryp) metabolic pathway, those analytical approaches should also be able to determine at least 5-HT and 5-HIAA in biological samples. L-Tryp is a neutral, essential amino acid and it is the only amino acid in proteins derived from indole, a bicyclic ring formed by a benzene and a pyrrole group. The L-Tryp indole ring in –R residues assists the stabilization of protein and peptide structures and allows their interactions through Van der Waals forces, whereas the indole ring in –N residues demonstrates susceptibility as a hydrogen bond donor, which could also explain the role of L-Tryp in protein binding and recognition. Furthermore, L-Tryp is an essential precursor of various bioactive compounds such as kynurenines and serotonin (5-HT) (Figure 1). L-Tryp is converted to 5-HT by enzymes in intestinal and nervous tissue. Only a very small amount of dietary L-Tryp is converted into 5-HT and about 90% of L-Tryp biotransformation consists of the breaking of the indole ring and turn over into the kynurenine (KYN) pathway and nicotinic acid (NU). 

A study of the basic biology of the L-Tryp metabolism pointed out the relevant issue of the bioavailability of this amino acid or changes in the regulation of its metabolism in tissues. Therefore, studying the unbalanced transformation of this amino acid during the pathological stages of the organism is relevant. Serotonin synthesis occurs in the periphery within the gut neurons and enterochromaffin cells and in the central nervous system within the neurons of the raphe in the brain stem. L-Tryp is hydroxylated to 5-hydroxytryptophan (5-HTrp) by the enzyme tryptophan hydroxylase type 2, the rate limiting step in brain serotonin synthesis. Furthermore, decarboxylation, via the enzymatic action of the L-aromatic acid decarboxylase, occurred leading to the formation of 5-HT. 5-HT is further decomposed with monoamine oxidase type A (MAO-A) and aldehyde dehydrogenase to its main metabolite–5-HIAA [6,7,8]. 

**Figure 2:**
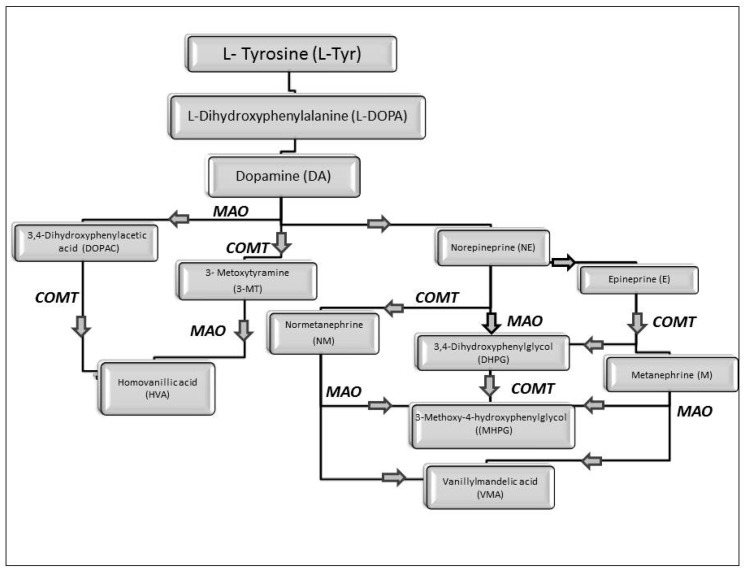
The main products of the L-Tyr metabolic pathway.

While studying the L-Tyrosine (L-Tyr) metabolic pathway, the compounds demonstrated in Figure 2 must be carefully discussed. L-Tyr is the relevant element of protein synthesis and serves as a precursor to catecholamines, tyramine⁄octopamine, melanin pigment and thyroid hormones. L-Tyr is synthesized from the essential amino acid phenylalanine via the action of the enzyme phenylalanine hydroxylase. Further transformation involves the hydroxylation of L-Tyr at the phenol ring by tyrosine hydroxylase to L-dihydroxyphenylalanine (L-DOPA) and its subsequent decarboxylation to DA by the aromatic amino acid decarboxylase. DA is an important neurotransmitter in the brain but a meaningful part of overall DA in the organism is produced by the mesenteric organ. DA is subsequently metabolized into several metabolites, like 3,4-dihydroxyphenylacetic acid (DOPAC) and homovanillic acid (HVA). DA is also the precursor of E and NE. All the compounds involved in the L-Tyr metabolic pathway except DA are chiral and only the L-enantiomers of the involved amino acids can lead to these important neuroactive molecules. Indeed, L-amino acids are the most abundant, naturally existing forms of those molecules in the human body [9]. Important but not yet widely studied, metabolites of the L-Tyr pathway are 3,4-dihydroxyphenylglycol (DHPG) and 3-methoxy-4-hydroxyphenylglycol (MHPG). DHPG is produced by the metabolism of NE by MAO and could be further transformed by catechol-O-methyltransferase (COMT) to MHPG. MHPG can also be formed by the deamination of normetanephrine (NM) and metanephrine (M). COMT also catalyses the methyl conjugation of DA and DOPAC, which results in the formation of 3-methoxytyramine (3-MT) and HVA, respectively. COMT allows the inactivation of the catecholamines and is the most important regulator of the prefrontal dopamine function. Further changes in MHPG lead to the formation of VMA, the principal end-product of NE and E metabolism. VMA is formed when MHPG is submitted to oxidation and catalysed by the sequential actions of alcohol and aldehyde dehydrogenases [10,11,12].

As it was mentioned above, data based on dynamic concentration changes of endogenous BAs and their metabolites in the human body play an important role in the diagnosis and patient therapy of several diseases. This allows one to estimate the health state of the patients and their response to applied treatments as well as to fully understand the pathogenesis of oncological, endocrinal, cardiac or neurodegenerative diseases. Due to the fact that physiological as well as pathological concentrations of BAs in human specimens are extremely low, the optimization of new, straightforward analytical separation and detection methods is needed. Furthermore, the methods should allow the determination of multiple biomarkers within one run since none of the above-mentioned endogenous compounds are specific and selective enough for the unbiased diagnosis of the disease.

**Figure 3:**
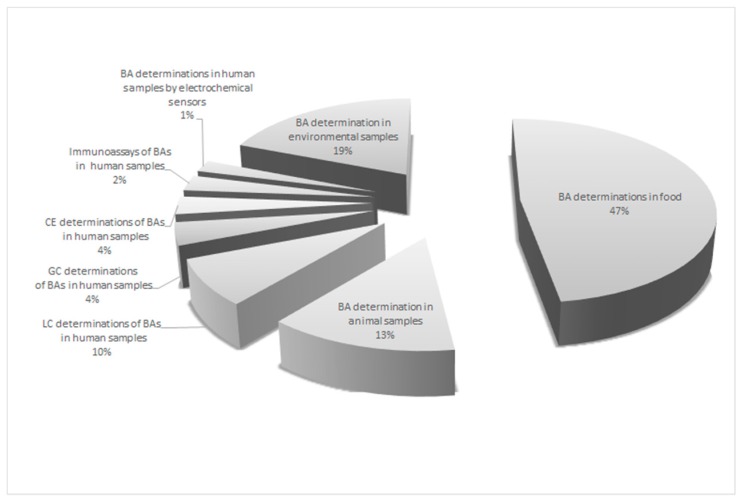
The distribution of the publications describing the methods for the quantification of biogenic amines (BAs) in various matrices.

A lot of publications can be found in the literature related to the determination of BAs in food products, while less have focused on the development of methods for the analysis of the compounds of interest in biological matrices, including human specimens (Figure 3). The methodologies for BA identification and quantification in animal and human matrices, mainly based on liquid chromatography coupled with tandem mass spectrometry (LC-MS/MS) have been summarized in a few review papers [13,14,15]. On the other hand, no report comparing both LC-MS/MS, GC and CE techniques in respect of non-separation approaches, like immunoassay and electrochemical sensor techniques, was presented.

The review aims to present recent advances towards the determination of BAs in human samples since 2010, with a special focus on sample preparation approaches and LC, GC and CE techniques. Furthermore, those techniques are compared to non-separation methods and additionally discussed, not only from the analytical point of view but also clinical, since the review comprises the newest discoveries that involved the measurement of BAs in human fluids as biomarkers of oncological, neurological, cardiologic and endocrine patients.

## 2. Sample Preparation

For the effective application of separation techniques during qualitative and/or quantitative analysis, extraction methods need to be developed and optimized for the selective isolation of analytes of interest from biological samples. Because of the very low levels of biogenic amines in biofluids such as plasma, urine or cerebrospinal fluid (CSF), the procedures for analyte extraction and preconcentration are crucial to develop more sensitive and selective assays. The developed methods must not only meet the validation criteria required from analytical methods but also allow the determination of the analytes at the appropriate, frequently nanomolar, concentration level for effective pharmaceutical or clinical application. To achieve this goal, it is necessary to select the appropriate type of biological matrix, in which the analyte will reflect the current state of the body. Moreover, the proper selection of the extraction procedure to prepare the samples before analysis requires optimal conditions. This stage includes, beyond the selective isolation of analytes, the also-mentioned preconcentration of analytes, derivatization (if it is required by the detection method) and the purification of the sample from ballast substances. It should be noted that the choice of the sample preparation procedure is strongly dependent on the physicochemical character of the analytes (Table 1), the type of biological matrix, the type of separation method and the type of detection.

The body fluids that are usually used for the clinical analysis of BAs are serum, plasma or urine. Urine is a biofluid of major interest because of its fairly simple, non-invasive and inexpensive collection and availability in large quantities. These attributes make urine a practical choice for developing biomarker studies. Although, as the literature data indicated, the neurotransmitter levels in paediatric patients were estimated using different biological matrices, urine was very often used in this analysis. However, in order to precisely estimate the BA levels in urine samples, it is necessary to collect 24-h urine samples. Unfortunately, this approach seemed impractical in the case of paediatric patients. Therefore, more often, plasma samples or random urine could be selected as a reliable matrix as a fast and sensitive method for determining a panel of neurotransmitters. Occasionally, BAs are isolated from CSF. It should be added that CSF is a very good biofluid but strongly difficult and invasive to collect [16]. It is a watery liquid that circulates within the ventricles of the brain and surrounds the brain and spinal column. It contains salts, enzymes, proteins, peptides and small molecules, including BAs, that play critical roles in many physiological processes and may reflect pathological processes in the CNS. As was emphasized by Taj et al. [17], the conventional laboratory examination of CSF for both the diagnosis and treatment of various CNS infections consists of CSF pressure, measurement, total and differential leukocyte count, glucose and protein quantification, Gram stain, bacterial and viral culture and the polymerase chain reaction (PCR) of different etiological agents. The authors focused on the determination of BAs in CSF, which may additionally contribute to developing new diagnostic tools of CNS infections. The researchers proved that the analysis of BAs in CSF samples reflected a correlation between the concentrations of some BAs with infection-associated ethology. The authors emphasized that infections may disturb the neurotransmitter degradation pathway. The results of this work indicated the complete degradation of DA into its metabolic products, that is, DOPAC and HVA in *herpes simplex virus* infection. In turn, in *Listeria monocytogenes* infection, DA is catabolized into DOPAC and devoid of further degradation into HVA. Moreover, in the literature, the fact is documented that elevated levels of neurotransmitters in CSF confirm the disruption of the blood brain barrier during meningitis [18]. Likewise, there are also significant differences in the concentrations of BAs in the matrix according to the body fluid. For example, the amount of DA in the urine of a healthy person is up to 10 times greater than the amount of NE, while the total plasma catecholamine content is 10 times higher than the content of NE [19]. For the comprehensive analysis of BAs, it is necessary to develop preparation procedures for samples of different body fluids. The effect of the sample preparation procedure of biological material (blood, urine) on the content of selected BAs depends on the type of sample matrix and the chemical nature of the analytes, including pKa (Table 1). It is also very important to ensure the stability of analytes during collection and storage. An effective and frequent practice to prevent the decomposition of BAs was the acidification of the sample to a pH below 3 using 6 M HCl. Many authors recommend this method, because BAs remain stable during storage at room temperature for 3 days and at −80 °C even to 10 months [20]. In turn, the initial deproteinization of the sample and the type of applied extraction technique affect the degree of purification of the sample of interfering substances, for the selective isolation of analytes from a sample matrix and their potential preconcentration. The challenge is due not only to the complexity of biological fluids (urine, blood, plasma, saliva, etc.) but also to the volume of the sample (optimization of the starting sample volume), the analyte content, the wrong physical state for the analysis method and interfering matrix components that may give a false-positive or a negative reading in the measurement. For this reason, a lot of sample preparation procedures have been described in the literature. The most commonly used clean-up techniques, which allow a high concentration degree of analytes, are *liquid-liquid extraction* (LLE), *dispersive liquid-liquid extraction* (DLLME), *solid-phase extraction* (SPE), *solid-phase microextraction* (SPME) and their different varieties. Each of them provides a different degree of preconcentration of the analytes (*off-line preconcentration*). In the case of BAs, this is particularly important, because their concentration in biological matrices is very low (ng, pg or less) and additionally, BAs have a very hydrophilic character, poor ionizability and instability. According to Cudjoe and Pawliszyn, the sampling of highly hydrophilic molecules in biological matrices is characterized by low recoveries and relatively poorer chromatographic separation was achieved [21]. However, some reports describing methods for the quantification of BAs in human matrices based only on the dilution of the sample have also been reported in the literature.

### 2.1. Sample Pretreatment Based on the Dilution of the Sample

As it was mentioned above, the simplest way to prepare a sample for further separation analysis is just dilution. This approach was applied mainly to determine BAs in urine samples. Remane et al. tested this procedure to quantify many exogenous and endogenous compounds, including HVA and indole-3-acetic acid (IAA) in 50 µL of urine samples. The limits of quantification (LOQs) for BAs were in the range of 45 mg/L for HVA to 63 mg/L for IAA, which indicates that low extraction efficiency was obtained [22]. A similar urine sample volume, collected from patients suffering from carcinoid tumour, was investigated by Clark et al. to determine 5-HIAA in the range of 0.5 to 100 mg/mL by LC with tandem mass spectrometry (LC-MS/MS) [23]. In turn, Marcos et al. used 100 and 150 µL of urine and plasma samples, respectively, to determine L-Tryp and L-Tyr metabolites in patients with neurological and inflammatory disorders [7]. The urine sample was 10-fold diluted with water, whereas the plasma sample was mixed with acetonitrile (ACN) in order to precipitate the proteins before the LC-MS/MS analysis. This method achieves limit of detection (LODs) values ranging from 10 ng/mL for DA and 5-HT to 1 µg/mL for HVA in urine and from 0.5 ng/mL for 5-HT to 100 ng/mL for L-Tryp and L-Tyr in plasma. These data clearly confirmed that deproteinization was more effective, because lower LODs were calculated for the analytes, for example 10 versus 0.5 ng/mL for 5-HT in urine and plasma, respectively. In turn, recovery values were from 55% for DA to 97% for HVA in urine and from 59% for 5-HT to 94% for 5-HIAA in plasma. Zhao et al. used a sample preparation procedure based on the centrifugation of the urine sample (200 µL) and dilution in water before high performance liquid chromatography with ultraviolet detection (HPLC-UV) or fluorescence detection (HPLC-FD) to determine L-Tryp and its three metabolites. The obtained LOD for L-Tryp was 0.02 µmol/L and 0.01 µmol/L for 5-HIAA [24]. The extraction efficiency expressed as recovery was in the range of 96.7–105.2% for L-Tryp and 96.1 to 99.7% for 5-HIAA.

### 2.2. Deproteinization

Deproteinization is another approach in sample treatment, which allows the removal of ballast, such as proteins, from the biological sample, which can significantly increase selectivity and sensitivity. According to the literature data, a lot of methods for the quantification of BAs in human specimens were based on deproteinization using acetone [25,26], ACN added to the samples in the ratios of 2:1 or 1:1 (*v*/*v*) [26,27,28,29], methanol (MeOH) with zinc sulphate in water (80/20, *v*/*v*) [30], 0.05% (*v*/*v*) formic acid (FA) [23,31], 0.6 M perchloric acid [32] or 10% trichloroacetic acid (TCA) [33]. Cai et al. applied ice-cold ACN for the deproteinization of a 0.3 mL plasma sample, followed by derivatization with dansyl chloride [27]. This approach was applied for the determination of DA, DOPAC, HVA, NE, VMA, MHPG, 5-HT, 5-HIAA and other analytes. The authors achieved good purification of the sample and low values of LOQs ranging from 0.27 to 1.62 pmol/mL. In turn, Fang et al., for the determination of 4-hydroxy-3-methoxyphenylglycol sulphate (HMPG sulphate), VMA and HVA present in 500 µL sample of human serum, applied deproteinization with ACN, while a 500 µL urine sample was diluted with water and directly analysed by LC-MS/MS [29]. The method provides LODs of 0.2 ng/mL for VMA, 0.03 ng/mL for HMPG sulphate and 0.7 ng/mL for HVA and high recovery results for HVA-90.6%, VMA - 101.6% and 111.1% for HMPG sulphate. Exceptionally, only centrifugation was used as way to remove protein from urine samples [34].

### 2.3. Derivatization

Several different approaches have been reported to increase the sensitivity and selectivity for the LC–MS/MS detection of BAs in different human matrices, including derivatization. Only evaporation and derivatization with phenyl-isothiocyanate (PITC) on a 96-well filter plate was used by Zheng et al. for the isolation of DA, 5-HT, tyramine, phenylethylamine (PEA), E, NE, M and NM from 25 µL samples of human serum [35]. This procedure, coupled with LC-MS/MS, provided a nanomolar limit of detection and recovery rates of spiked serum samples in the range of 93.2–113%. Moreover, to avoid the decomposition of analytes, the samples were thawed on ice, in the dark.

To identify previously unrecognized biological pathways and biomarkers that might expand the inflammatory hypothesis of depression, a similar method, based on the derivatization of selected BAs with phenyl-isothiocyanate (PITC), was used by Baranyi et al. [36]. The application required only 10 µL of plasma samples and the analytes were eluted with 5 mM of ammonium acetate in MeOH and separated by LC-MS/MS. Vermeiren et al. used benzylamine and 1,2-diphenylethyleendiamine (DPE) for the derivatization of NE, 5-HT, 5-HIAA, DA, DOPAC and HVA present in CSF samples collected from patients with Alzheimer’s Disease (AD), as well as non-AD dementia patients [3]. Sakaguchi et al. applied sample derivatization with 2-(perfluorooctyl)ethyl isocyanate (PFOEI) for the determination of L-DOPA, DA, NE, E, M, L-Tryp, 5-HTrp and 5-HT in 10 µL of urine samples, reaching LODs from 0.21 to 4.2 nmol/mL and with recovery results from 96.9 to 103.3% [37]. In a different study concerning plasma samples, Sakaguchi et al. used tetrahydrofuran (THF) and 4-(heptadecafluorodecyl)benzylamine (HFBA) to deproteinize and derivatize 5-HT, 5-HIAA and three other 5-hydroxyindoles in 70 µL of plasma samples [38]. This method provides LODs of 4.3 pmol/mL for 5-HT and 1.5 pmol/mL for 5-HIAA but the extraction efficiencies were worse and equalled 84.2% for 5-HT and 72.2% for 5-HIAA. For pre-column derivatization with a fluorescence reagent, (*R*)-(–)-4-(*N*, *N*-dimethylaminosulfonyl)-7-(3-isothiocyanatopyrrolidin-1-yl)-2,1,3-benzoxadi-azole (DBD-PyNCS) was used by Ohashi et al. for the determination of L-Tryp and kynurenine (KYN) in 10 µL of human serum samples by the LC-MS technique [39]. This methodology allowed LODs of 150 nM to be achieved for both analytes. The obtained sensitivity and selectivity was enough for the quantification of altered L-Tryp and its metabolite levels in various disease states [40].

In turn, Ellis et al. used only the dilution of 50 µL of urine with formic acid and derivatized NE, E, DA, NM, M and 3-MT with acetaldehyde solution to mono-ethyl or diethyl derivatives [41]. The results showed that the conversion of BAs to less polar ethyl derivatives increased their mass and enhanced the intensity of their molecular ions and fragments. Ethylation also improved the chromatographic properties of the amines, with greater retention and elution from reverse-phase LC columns. The signal response of MS/MS detection was increased up to 50-fold for ethyl metanephrines compared to non-derivatized compounds. This increase allowed for the omission of SPE as a clean-up step prior to the analysis of BAs in urine samples.

### 2.4. Microdialysis

Similarly, the *on-line* microdialysis technique described by Tang et al. allowed the sample preparation time to be significantly shortened while providing sensitivity. For example, the LOD of 2 pg was calculated for 5-HT, while 10 pg was found for DA and NE in carcinoma stem cell samples (ECSC) [42]. Microdialysis is one such technique that is widely used by perfusing a fluid across a semipermeable membrane in a probe inserted into an area of interest.

### 2.5. LLE

A sample preparation procedure for BA isolation from human specimens based on liquid-liquid extraction (LLE) was mainly performed using ethyl acetate [43,44,45] and TCA [46] as extraction solvents. This technique is especially popular when urine samples are investigated. Sadilkowa et al. used ethyl acetate for the extraction of VMA and HVA from 0.5 mL of serum samples instead of 24-h urine [43]. Thanks to the application of LC-MS/MS, the LODs were 0.02 ng/mL for VMA and 0.18 ng/mL for HVA, while the extraction efficiency, expressed as the recovery, was found to be 110.5% for VMA and 108.0% for HVA, respectively. The same extraction solvent was used in studies described by Diniz et al. [44] and Tran et al. [45] allowing the recoveries of the analytes extracted from 1 mL of urine samples to reach 92.1–108.8% and 92–96%, respectively.

Gosetti et al., during the determination of many analytes, including E, DA, 3-MT, NE, 5-HT and L-Tyr, from 50 µL of urine samples, used LLE with TCA [46]. This allowed LODs to be achieved from 0.3 to 6.6 μg/L and recoveries from 72.9 to 100.0%. Additionally, TCA avoids catechol group oxidation in this study. A more complicated method was elaborated by Husek et al. for the determination of 56 amino acids and their conjugates, 84 organic acids, 9 biogenic amines and 4 other polar analytes in 25 µL of human urine samples [47]. They applied liquid-liquid microextraction (LLME) with a tris(3-hydroxypropyl)phosphine (THP) reducing agent and re-extraction into an isooctane with HCl prior to in-situ derivatization of amino-carboxylic metabolites with 1,1,1,2,2,3,3-heptafluorobutyl chloroformate reagent (HFBCF) and GC–MS separation. High recoveries (>90%) for all analytes were achieved.

### 2.6. DLLME

Compared to LLE, a major advantage of the dispersive liquid-liquid microextraction (DLLME) procedure is the ease and speed of sample preparation. Initially, DLLME was elaborated for water and environmental samples and was rarely used for complex biological matrices, which require the removal of ballast, such as proteins. During optimizing this approach, the authors took into account a few important parameters affecting the efficiency of extraction, such as: the selection of extraction and dispersion solvents and their appropriate volumes, the effect of the sample pH and salinity on extraction efficiency. The selection of the proper extraction solvent and disperser solvent was the first and most important step in the DLLME procedure. The extraction solvent should possess several characteristics, including low solubility in water, a high affinity to analytes and a higher density and lower miscibility than the aqueous phase. Commonly used extraction solvents in DLLME are: chloroform, dichloromethane and carbon tetrachloride.

As reported by Konieczna et al., a DLLME procedure followed by LC-MS with a hydrophilic interaction chromatography (HILIC) column for the extraction of 13 compounds of different polarities, including BAs, was developed [48]. The studied BAs were extracted from 1 mL of urine (pH 2) using dichloromethane (extraction solvent) and ethanol (dispenser solvent). This achieved LODs of 5 ng/mL for E, 5-HT, Tyr, Tryp, 3-MT, 5-HIAA and VMA and 10 ng/mL for DA, NE, L-DOPA, 5-HTrp, HVA and DOPAC. The mean absolute recoveries were above 99.0% for all the studied analytes. Similar DLLME conditions were applied for the determination of these compounds in 250 µL samples of plasma [25]. Mean recoveries of the analytes ranging between 76.4% and 99.3% were obtained, while matrix effects did not exceed 15%. DLLME, using a mixture of ionic liquid (1-hexyl-3-methylimidazoliumhexafluorophosphate) as the extraction solvent, MeOH as the disperser and water containing fluorescence derivatization reagents (benzylamine and potassium hexacyanoferrate (III)), was applied by Hayama et al. for the determination of 5-HT, 5-HIAA and other analytes in 500 µL samples of human serum [49]. In this case, the mean recoveries ranged from 66 to 98% and the LOD values from 0.08 to 0.33 nM.

### 2.7. SPE

The isolation procedure based on solid-phase extraction (SPE) is a sample preparation process by which compounds are dissolved or suspended in a liquid mixture and are separated from other compounds in the mixture according to their physical and chemical properties. This technique has been widely employed to concentrate and purify samples for analysis. During SPE, steps including the removal of interfering compounds and the enrichment of the analytes with different types of sorbents, such as: strong cation exchange (SCX), weak cation exchange (WCX) and mixed mode cation exchange (MXC), hydrophilic-lipophilic balanced copolymer (HLB), alumina B and C18, during the isolation of BAs from human matrices, were investigated. Moreover, SPE can be used to isolate analytes of interest from a wide variety of matrices, including urine, whole blood, plasma, serum, cerebrospinal fluid or human peripheral blood mononuclear cells [16,50,51,52,53,54,55,56,57,58,59,60,61,62,63,64,65,66,67].

Ion-exchange SPE cartridges were used for compounds that are charged when in a solution. Cationic (positively charged) compounds are isolated by using SCX or WCX silica-bonded cartridges. In this case, the retention mechanism of the compound is based mainly on the electrostatic attraction of the charged functional group on the compound to the charged group that is bonded to the silica surface. In order for a compound to be retained by ion exchange from an aqueous solution, the pH of the sample matrix must be one at which both the compound of interest and the functional group on the bonded silica are charged. As was reported, silica-bonded sulfonic acid with Na^+^ counterion (SCX) for strong cation exchange was rarely used by Zhang et al. [54]. For the isolation of catecholamines from plasma or urine, WCX cartridges with silica-bonded carboxylic acid with Na^+^ counterion were more popular [53,55,64,68]. The carboxylic acid group is a weak anion, has a pKa of about 4.8, will be negatively charged in solutions of at least 2 pH units above this value and will isolate cations if the pH is one at which they are both charged and is thus considered (WCX). This solution achieves a high recovery of catecholamines from human samples, such as: >88% from 100 µL of plasma [53], >98% from 200 µL of plasma [55], ranging from 60 to 96% from 100 µL of urine [68] or ranging from 66 to 93% from 900 µL of plasma [64]. Woo et al., for the determination by LC-MS/MS of free E, NE, DA, M and NM from 100 µL of 24-h urine or plasma samples, applied the Strata-X-CW sorbent [50]. Moreover, to measure the total urinary metanephrines, the hydrolysis of the urine by HCl was applied before SPE was used. The elaborated extraction procedure provides recoveries ranging from 61 to 107%.

**Figure 4:**
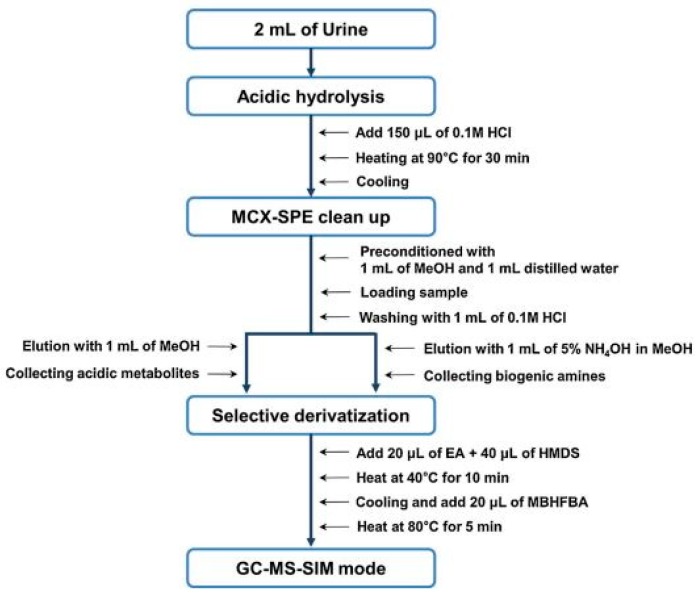
Overall analytical procedure for the analysis of biogenic amines and their acidic metabolites in urine by GC/MS in selective ion monitoring (SIM) mode. Figure adopted from reference [66] with the copyright permission.

For the effective extraction and clean-up of DA, HVA, L-DOPA, NM, E, NE, DOPAC, VMA, 5-HT and 5-HIAA from 2 mL samples of urine, MCX SPE cartridges were used by Park et al. [66]. The SPE procedure was also preceded by hydrolysis with HCl. In order to conduct the GC/MS separation analysis, according to the protocol presented in Figure 4, derivatization was also performed with hexamethyldisilazane (HMDS)/-N-methyl-bis-heptafluorobutyramide (MBHFBA).

The same procedure for the simultaneous determination and separation of many BAs from 2 mL samples of urine was applied by Shin et al. [67]. Similar LOD values, ranging from 0.47 ng/mL for DA to 1.72 ng/mL for VMA, were obtained.

A clean-up procedure with HLB sorbent, which is a universal polymeric reversed-phase sorbent developed for the extraction of a wide range of acidic, basic and neutral compounds from various matrices, was reported by Li et al., who carried out an isolation procedure of NE, E and DA from 0.6 mL of human urine mixed with a diphenyl borate (PBA) buffer (pH 8.5) [65]. In contrast to the majority of works in which the acidic condition was preferred, in this work, alkaline conditions allowed the stability of analytes to be effectively improved through the instant formation of a stable catecholamine-PBA complex. The same method was applied during the isolation of these compounds [59]. The elaborated method proved to be efficient enough to ensure recoveries in the range of 81.0–100.5% for the analytes. The limits of detection for NE, E and DA were 2.0, 0.5 and 2.0 pg/mL. A larger group of analytes, namely NE, E, DA, NM and M, were isolated by Li et al. from 10 μL of human urine samples under the same conditions reaching recoveries from 74.1% to 96.3% [63].

Alumina sorbent is a type of strong polarity adsorption sorbent used in the SPE technique, possessing properties similar to silica. Alumina B sorbent with a basic pH ~8.5, for the adsorption extraction of polar compounds and cation exchange, was used. The properties of Alumina B, for the isolation of E and NE from 350 μL of urine were used by Bergman et al. [60]. In turn, Zhang et al. applied this sorbent for the extraction of the mentioned BAs from 0.5 mL of human plasma [52]. The extraction efficiency obtained in both studies was above 66% for E and NE.

The study reported by He et al. described the application of a unique graphite carbon-based material in HyperSep Hypercarb SPE cartridges for the isolation of M and NM from 0.5 mL of plasma samples [57]. This kind of sorbent effectively retains highly polar compounds, which allowed LOQs to be reached of 7.2 pg/mL for M and 18.0 pg/mL for NM, whereas the recovery value was from 90.5 to 97.5% for both analytes.

Alternatively, microextraction by packed sorbent (MEPS), as a miniaturized form of SPE, has emerged as a new sample preparation technique with the advantages of using reduced extraction sorbent and solvent. Another great achievement of MEPS is the possibility of directly injecting the eluates (typically 20–50 µL) into LC, GC and CE systems without any modifications to the device, facilitating *on-line* coupling to any of these systems. The optimization of MEPS is focused on evaluating crucial parameters, such as sorbent type, sample pH, sample volume and elution conditions, strongly influencing the extraction efficiency. The choice of sorbent type is the most important step in eVols MEPS analysis due to the specific interactions of analytes with the stationary phase in the needle, mostly dependent on the physicochemical properties of the analysed compounds and on the chemical composition of the sorbent. Konieczna et al. used MEPS to isolate 12 BAs from 50 µL of urine and 100 μL of plasma samples [69]. In order to select the appropriate sorbent for the measurements of multiple neurotransmitters with different polarities, four commercially available eVols MEPS sorbents (C8, C18, amino-propyl silane (APS) and MIX having both C8 and C18 groups) were investigated. This is due to the fact that most analysed BAs are very polar compounds and the polarity of the APS sorbent was the highest, therefore this sorbent can be found as the most effective. The recovery results were between 87.6–104.3% for plasma and 84.2–98.6% for urine, respectively. Another study reported by Oppolzer et al. shows the application of MEPS for the extraction of 5-HT, DA and NE from 500 µL of human urine [70]. The obtained recovery results were from 91.97% to 110.06%, whereas the LOD was 20 ng/mL for 5-HT and 2 ng/mL for DA and NE. In turn, Saracino et al. applied MEPS for the first time to extract NE, E and DA from dried plasma spots (DPSs) and dried urine spots (DUSs) as well as from samples of 150 µL of human plasma and 10 µL of urine [71]. In general, the analytes studied in dried matrix spots are reported to be fairly stable; in fact, the use of dried samples affords numerous advantages for clinicians and treatment providers, such as low biohazard risk and costs, feasibility and easiness for sample storage and transport to laboratories. In this study, the LOD was 0.03 ng/mL for all analytes, whereas recovery values were between 86.0–95.2%.

### 2.8. SPME

During the determination of BAs for the sample preparation step, also another alternative technique, solid-phase microextraction (SPME) was used. SPME holds some advantages over traditional sample preparation methods, such as the possibility to perform preconcentration and extraction in one step, no consumption of toxic and non-environmentally friendly organic solvents and the relative ease of online coupling to chromatographic systems. Comparable to other extraction techniques, in the case of SPME, the key extraction parameters, such as the type of SPME sorbent, pH and salinity of the sample, the type of extraction and desorption solution, the time of the extraction/desorption process and the temperature have to be optimized. For example, Lindstrom et al. applied SPME before LC-MS/MS assays for the determination of serum 5-HT and 5-HIAA in the diagnosis of serotonin producing neuroendocrine neoplasms (NENs) [72]. In this study, 100 µL samples of serum were analysed on 96-well Oasis WCX μElution plates (SPME) and washed with a mixture of methanol/formic acid/water (30/5/65, *v*/*v*). The analytes were eluted with 3% 90 mmol/L ammonium formate in ACN (pH 4). In these conditions, the recovery value was between 95–115% for both analytes, while the LODs and LOQs were 2.5 nmol/L and 10 nmol/L, respectively. Monteleone et al. carried out an analysis of HVA, VMA and 5-HIAA in 50 µL samples of human urine supported by derivatization with methyl- or ethyl- chloroformate and SPME [73]. In this study, the efficiency of the SPME fibre at adsorbing the analytes was tested, resulting in physicochemical properties achieved by the derivatized analytes as a function of the added moieties and used SPME fibres. Finally, the polyacrylate (PA) fibres in immersion mode were selected as the most efficient for the extraction of the analytes. The LODs were 1.3, 0.046 and 24.3 µg/L for HVA, VMA and 5-HIAA, respectively. Under the same extraction conditions, Naccarato et al. detected DA, 5-HT and NE in 600 µL of human urine [74]. However, the recovery results were not shown in both studies.

## 3. Analytical Strategies of BA determinations Based on Separation Techniques

As it was earlier mentioned, the identification and quantification of BAs in human samples is a challenging task because of variable concentration levels, which are mainly very low, the need of the simultaneous determination of many analytes in one sample or only selected compounds in the presence of others having very similar physicochemical characteristics, as well as the complexity of the matrix sample, which contains a lot of potentially interfering substances. Moreover, most BAs are highly polar, which causes the poor solubility of these compounds in organic solvents (Table 1). Additionally, the lack of the intrinsic properties required for the application of detectors commonly applied in many laboratories makes the quantification of BAs difficult. Many various methods have been developed for the determination of these analytes in human samples. However, the most popular are separation techniques based on chromatographic and electromigration techniques. Among them, high-performance liquid chromatography (HPLC) is the predominant method, while gas chromatography (GC) and capillary electrophoresis (CE) are relatively rarely applied.

### 3.1. Conventional HPLC

As mentioned above, BAs are low-molecular weight substances which possess a very high polar nature and a positive charge under acidic conditions. For years, these compounds have separated on conventional reversed-phase C18 columns due to fast elution and the relatively short run-time of the analysis. The monitoring of these analytes was commonly based on ultraviolet (UV) or combined ultraviolet and visible radiation (UV/Vis) detectors, as well as diode array detection (DAD), which is able to register light intensity between 190 and 600 nm. Both UV-Vis and DAD detectors belong to the standard equipment of many laboratories and staff are usually experienced in using them, making this detection method attractive for many pharmaceutical and clinical applications. It should be noticed that despite the fact that there are a great variety of chromatographic columns available on the market, since 2010, the reported conventional methods for the quantification of BAs were based only on C18 stationary phases (Table 2). For example, a C18 column and UV detector at λ = 254 nm was applied for the LC analysis of E and NE in human serum [75]. The same type of stationary phase was used for the quantification of L-Tryp and its metabolite in human plasma using UV detection at 280 nm [32]. Unfortunately, major BAs exhibit low absorption at Vis and UV wavelengths, which means that a lot of LC-UV(DAD) methods are not sensitive and selective enough for many specific clinical applications. For the improvement of these parameters, relatively large volume samples, extensive sample preparation and/or tedious, time-consuming derivative procedures were commonly applied before the LC-UV analysis which was earlier described. Another approach to overcome these limitations was the application of a fluorescence (FL) detector, especially that major BAs exhibit native fluorescence. FL detection allows significantly lower values of LOD and LOQ of the compounds of interest to be obtained in comparison to with UV. However, the emission wavelength for these analytes is short and it is not enough for their quantification at small concentrations in real biological samples.

**Figure 5:**
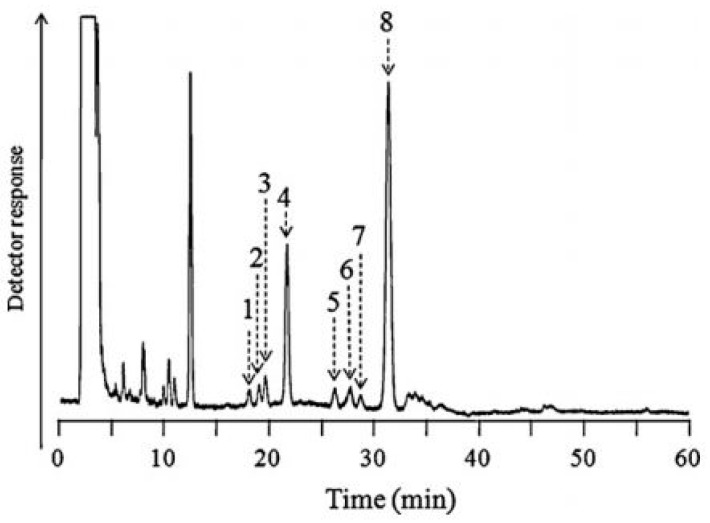
Chromatogram obtained from urine sample with PFOEI derivatization using fluorous LC separation. LC conditions: Column Fluofix-II 120E column (250 mm x 4.6 mm i.d.): mobile phase, acetonitrile-water-trifluoroacetic acid (60:40:0.05, *v*/*v*): flow rate, 1.0 mL/min: column temperature, 30 °C. Peaks and concentrations (nmol/mL urine): 1, DOPA (6.4): 2, NE (4.3): 3, 5-HTrp (1.6): 4, E (1.7): 5, DA (5.2): 6, 5-HT (2.6): 7, MN (3.4),: 8, Trp (26). Figure adopted from reference [37] with the copyright permission.

Therefore, pre-column or post-column chemical derivatizations have frequently been performed to increase the sensitivity and selectivity of BA determinations. The choice of pre-column or post-column derivatization mode is dependent on the equipment of the laboratory. Post-column derivatization requires additional instrumentation in the HPLC system, which can be omitted using pre-column derivatization. In each case, the optimization of the fluorescence reaction should be performed, which involves the selection of the optimal derivatizing agent and its concentration, the temperature and the reaction time. Due to the fact that these reactions can be long, automation of the fluorescence procedure is recommended. As it was earlier mentioned, the main chemical reagents for fluorescence derivatization of BAs include benzylamine, ethylenediamine or meso-1,2-diphenylethylenediamine (DPE), which react with BAs in the presence of potassium hexacyanoferrate (III), 2-(perfluorooctyl)ethyl isocyanate (PFOEI), as well as 4-(heptadecafluorodecyl)benzylamine (HFBA). Figure 5 presents representative chromatograms obtained by LC-FD for the quantification of eight BAs in urine after derivatization with the use of PFOEI and separation performed on a C18 stationary phase. This method allowed LODs to be obtained in the range of 0.21–4.2 nmol/mL [37].

Another derivatizing agent, such as HFBA, was applied before the LC-FL analysis of 5-HT and 5-HIAA in plasma, based on a fluorous phase column [38]. Moreover, the derivatization of the abovementioned analytes with benzylamine and potassium hexacyanoferrate (III) before the LC-FL assay based on RP18 was also reported [49]. However, the efficiency of this method was significantly lower despite the fact that a large sample volume was used. Unfortunately, the LC-FL technique for BA determinations also suffers from major drawbacks because even after derivatization with chemical agents, fluorimetric detection may be disturbed by endogenous co-eluting compounds.

This has meant that in recent years, the importance of the conventional HPLC method based on FL detection for BA quantification has systematically decreased. Nowadays, this methodology has effectively been replaced by ultrafast performance liquid chromatography (UPLC) with FL detection. For example, the UPLC-FL method based on a C18 stationary phase and derivatization with benzylamine and DPE, instead of HVA, was used for the quantification of NE, 5-HT, 5-HIAA, DA and DOPAC in CSF [3]. This allowed the quantitation of the analytes with LODs of 50 pmol/L. The UPLC technique has been described in more detail in Section 3.3.

Another interesting alternative to obtain more sensitive assays is the use of electrochemical detection (ED), due to the fact that BAs are electroactive compounds. ED detection exists in two forms: coulometry and amperometry. In both, the current generated by the reaction is directly proportional to the concentration of the analyte in the solution. However, coulometric detection is considered as more sensitive and specific compared to amperometry, which allows smaller sample volumes to be used and lower detection limits obtained. According to the literature data, the LC analysis of 5-HT, DA and NE in human urine was performed on a C18 column using an ED detector based on amperometry for monitoring the analytes [70]. It allowed the quantification of 5-HT at the level of 50 ng/mL while the LOQ for DA and NE was 5 ng/mL. However, a relatively large sample volume (0.5 mL) and a tedious sample preparation procedure based on MEPS were required for the improvement of sensitivity and selectivity.

**Figure 6:**
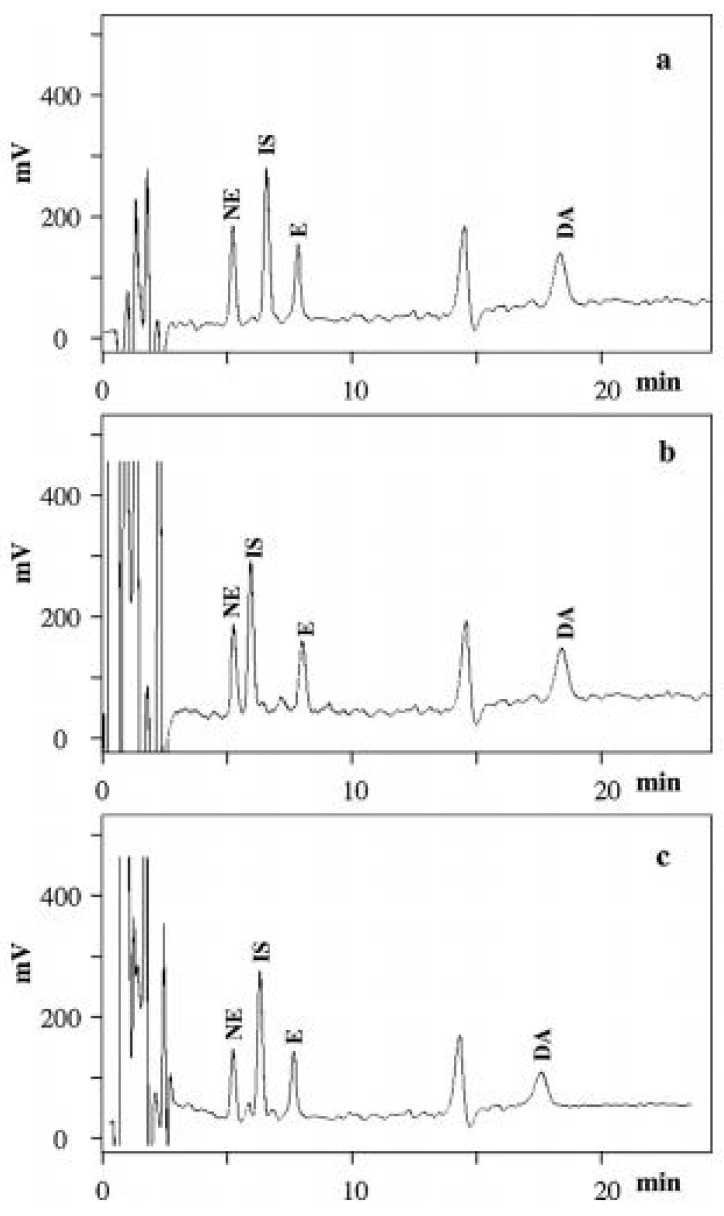
Chromatograms of (**a**) standard solution: (**b**) blank DPS: (**c**) blank DUS. The on-column concentrations were 5 ng/mL for NE, E and DA: 2.5 ng/mL for IS. Figure adopted from reference [71] with the copyright permission.

A coulometric detector was applied for monitoring NE, E and DA in plasma, urine, DPSs and DUSs samples, where the MEPS procedure with separation on a C18 stationary phase was also performed. This analytical procedure allowed significantly lower LODs and LOQs to be obtained for the compounds of interest (Figure 6) [71].

Summarizing, in recent years, the importance of conventional HPLC methods for determining BAs in human biological samples has systematically decreased due to not enough sensitivity and selectivity for many pharmaceutical and clinical applications. Nowadays, LC-MS or LC-MS/MS have become predominant techniques for BA quantifications.

### 3.2. LC-MS/MS

The wide application of LC-MS/MS for measurements of BAs in clinical laboratories is related to the many advantages of this analytical technique, such as high specificity and sensitivity, a wide concentration range, minimal sample preparation, high-throughput and the ability of detecting both free and conjugated forms of BAs. It is related to the fact that mass spectrometry is able to quantify and identify compounds on the basis of a mass-dependent transition between the precursor ion and the product ions. Selective reaction monitoring (SRM) or multiple reaction monitoring (MRM) chromatograms of the transitions are extracted after the positive or negative ionization of the compounds of interest. In the case of BA determinations, mainly electrospray ionization (ESI) was used (Table 2). It is related to the fact that ESI is preferred for polar molecules. Moreover, it also offers other advantages, like the ability of the precise and accurate quantification of low- and high-molecular weight compounds, high sensitivity and selectivity, as well as an amenability to automation. Therefore, most BA quantifications were performed using ESI in the positive mode, which is preferred for amines, although the negative mode was also applied, especially for VMA, HVA and DOPAC (acids) (Table 1) [25,29,34,39,43,48,58]. Another type of ionization, such as atmospheric pressure chemical ionization (APCI), which is preferred for molecules with a low to medium polarity, was also applied for the quantification of BAs in urine samples [46]. The chromatograms were mainly extracted in MRM [25,26,29,30,31,35,41,42,44,54,55,59,60,62,64,65,68,76,77]; although the SRM mode was also used for BA quantifications [7,28,57]. However, analysis in MRM mode is preferred in clinical practice to avoid false-positive results. An interesting approach is also the application of the MRM^3^ mode, which can improve the reliability of the obtained results [78]. Therefore, the LC-MS/MS technique was applied for the identification and the quantification of BAs in serum [29,31,35,39,43,49,58,61,72,75], urine [7,22,23,24,29,31,32,34,37,41,44,46,48,50,60,63,65,68,69], plasma [7,16,25,26,27,30,32,38,52,53,57,69,71], whole blood [51] and other human samples [3,16,31,42,59,76] (Table 2). A method based only on ESI-MS/MS for the determination of 13 amino acids in human plasma, including L-Tyr, has also been reported [56]. This type of detection is considered as adequate for the direct monitoring of BAs but derivatization, as it was earlier mentioned, leads to a significant improvement in sensitivity and selectivity. For this reason, many LC-MS/MS procedures based on the pre-column or post-column derivatization of BAs using several derivatization agents, such as dansyl chloride [27], phenylisothiocyanate (PITC) [35], acetaldehyde [16,41,52], propionic anhydride with pyridine [54], 4-(*N*, *N*-dimethylaminosulfonyl)-7-(3-isothiocyanatopyrrolidin-1-yl)-2,1,3-benzoxadiaz-ole (DBD-PyNCS) [39] and perfluoroheptanoic acid (PFHA) [57], were applied.

**Figure 7:**
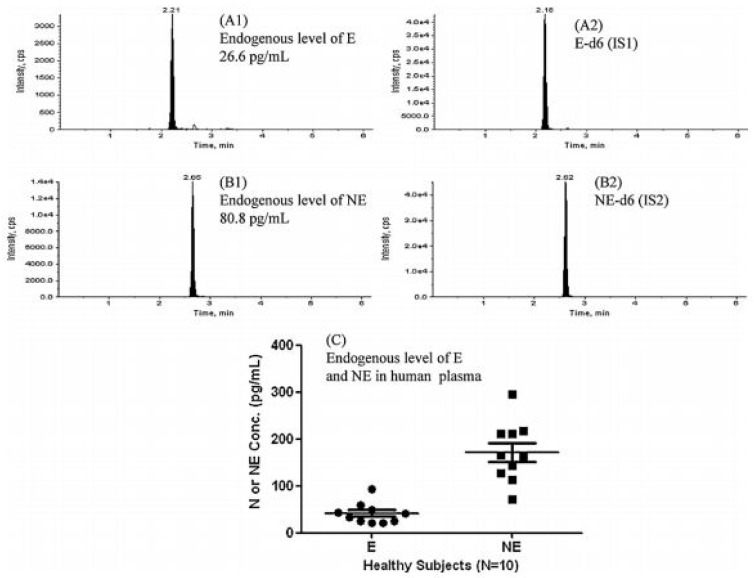
Representative LC-MS/MS chromatograms obtained while the monitoring of endogenous E and NE in the commercial healthy human plasma was carried out. (**A1**) 26.6 pg/mL of basal level of E in an individual human plasma, (**A2**) E-d6 (IS1), (**B1**) 80.8 pg/mL of basal level of NE in an individual human plasma. (**B2**) NE-d6 (IS2) and (**C**) the endogenous level of E and NE in 10 individual healthy human plasma samples. Figure adopted from reference [52] with the copyright permission.

It should also be noticed that several LC-MS/MS methods reported in the literature were based on traditional C18 stationary phases but most of them used shorter columns with a smaller diameter of the particles (mainly 3 microns) and a smaller inner diameter of the column (mainly ≤ 3 mm) in respect to conventional HPLC techniques. This allowed higher selectivity and sensitivity to be obtained [16,27,29,30,32,35,39,44,50]. On the other hand, the danger of the too fast elution and/or poor peak resolution of BAs in LC-MS/MS methods, because of their too low retention in the hydrophobic stationary phases, was not completely resolved. This analytical problem can be reduced by the application of columns having slightly different physicochemical characters. For example, an Atlantis T3 column with C18-alkyl phases bonded at the ligands with a lower density to those used in traditional hydrophobic stationary phases was used for the determination of BAs [31,41]. Another possibility was the use of a base-deactivated silica (BDS) column, which allowed the reduction of the peak-tailing of polar analytes by the reduction of unwanted silanol interactions of covalently-bonded silica stationary phases with the polar compounds [44]. The BDS column was applied for the effective quantification of E, NE, DA, M and NM in urine samples, with LOQs between 1 and 11.11 µg/L. Another interesting approach is the use of a porous graphitic carbon (PGC) column with a particular retention mechanism based on the interactions between polar compounds and the polarizable surface of graphite. The PGC column was able to quantify M and NM in human plasma at the level of 7.2 and 18.0 pg/mL, respectively [57]. A pentafluorophenyl propyl stationary phase can increase the retention of the protonated polar compounds. This column was applied for the effective LC separation of NE, E and DA in urine samples, with LOQs between 0.25 and 2.5 ng/mL [65]. The application of a C18 monolithic column possessing porous rods of silica instead of individual silica particles allowed for improvements in the separation and the quantification of E and NE (LOQs of 5.00 and 20.0 pg/mL for E and NE, respectively) (Figure 7) [52].

Another alternative was the use of hydrophilic interaction liquid chromatography (HILIC), which possesses a water-rich layer over the polar stationary phase. It means that the compounds of interest can interact with the mobile phase and the hydrophilic environment by hydrogen and electrostatic bonding. HILIC columns are especially suitable for the isolation of polar analytes in the presence of high matrix interferences. Moreover, they are able to enhance the sensitivity of the methods with electrospray ionization. HILIC columns were used for the separation of BAs in human serum [58,61,72], plasma [53,55], carcinoma stem cells [42], as well as in whole blood samples [51]. The disadvantage of the application of HILIC is the poor separation of O-methylated metabolites. The introduction of amide HILIC stationary phases allows these issues to be overcome, allowing the separation of these compounds while still retaining the benefits of HILIC [25,48,69,76]. Moreover, a mixed mode WCX stationary phase was used for the effective separation of BAs using ion-exchange chromatography [46], whereas a mixed-mode column containing both reversed-phase and anion exchange groups was applied for the analysis of 5-HIAA in human plasma samples [26].

### 3.3. UPLC-MS/MS

**Figure 8:**
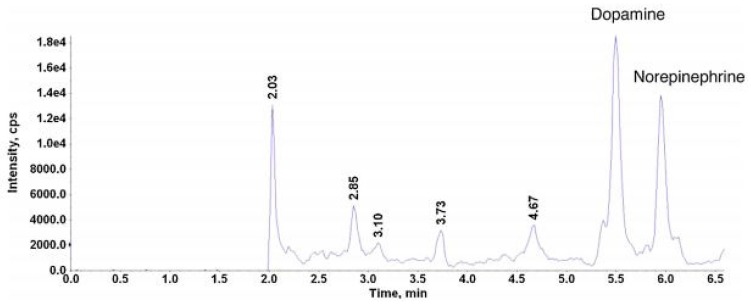
Chromatograms of dopamine and norepinephrine in a plasma sample. Figure adopted from reference [54] with the copyright permission.

Since 2004, new generations of stationary phases compatible with LC systems have been introduced on the market under the name Ultra Performance Liquid Chromatography (UPLC). In UPLC systems, short columns with a small diameter of the particles (<2 µm) and the inner diameter of the column within 2.0–2.1 mm are applied. It allows a substantial reduction in the dwell volume from 1-2 mL to about 120 µL. In consequence, the analysis time can be significantly decreased without loss in efficiency. In comparison to the conventional HPLC methods, UPLC shows many advantages, including higher throughput, better resolution, higher sensitivity and less solvent consumption. However, UPLC systems require specially designed chromatographic instruments because of the column back pressure, which increases to 1200 bar. According to the literature, the UPLC-MS/MS analyses of BA profiles in human samples were commonly performed on C18 [7,54,77], although other stationary phases were also applied [28,43,64,68]. For example, the representative chromatogram obtained by the LC-MS/MS method carried out on a UPLC BEH C18 column (2.1 × 100 mm, 1.7 µm) for the quantification of DA in the presence of NE in human neonate plasma was shown in Figure 8. This method was able to determine the analyte with LOQ of 10 pg/mL [54].

The UPLC-MS/MS assay of E and NE in human plasma using a BEH phenyl column supported by fully automated protein precipitation and reductive ethylation labelling allowed a LOQ of 0.05 ng/mL to be obtained for both analytes [28]. UPLC-MS/MS after LLE was performed on an HSS T3 column for the measurement of VMA and HVA in human serum samples [43]. The LOQs were 0.02 ng/mL for VMA and 0.18 ng/mL for HVA, respectively.

Despite many advantages, LC(UPLC)-MS/MS possesses some limitations, which should also be considered during method development. One of them is its susceptibility to matrix constituents, namely matrix effects which can enhance or suppress the ionization efficiency of the analytes. If such compounds co-elute with the analyte they can incorrectly change the response of the MS/MS detector. Moreover, isobaric interference can occur between the compounds with identical molecular masses, although this phenomenon is less problematic for BAs and their metabolites compared to other analytes (e.g., steroids). Additionally, ionic cross talk can occur for the analytes which are not chromatographically resolved and where in-source fragmentation can result in the formation of ions mimicking the compound of interest. It can lead to the over-estimation of concentrations of the analyte [79]. Therefore, matrix effects should be estimated, especially that they are mostly related to ESI sources [80,81]. The correction of the ion suppression/enhancement effect can be calculated when an internal standard is used during the development and validation of LC(UPLC)-MS or LC(UPLC)-MS/MS methods. Moreover, both MS/MS-based systems are expensive and require skilled laboratory personnel in implementing the methods, as well as specialized technical support for the cleaning and maintenance of the instruments. Another difficulty is the fact that these methodologies are more sensitive and specific compared to other detection techniques and there is a need to establish new reference intervals in terms of their implementation in clinical practice. So far, LC-MS/MS reference intervals have been calculated for many catecholamines and metabolites, including BAs [82,83,84,85,86,87,88].

### 3.4. Mobile Phase

Independent of the used LC system, the optimization of the mobile phase composition is extremely important because the physicochemical character of the mobile phase can decide on the success or defeat of each chromatographic separation. Therefore, the proportion of the organic modifier, the qualitative and quantitative composition of the buffer solution if it is applied as an aqueous phase, the selection of an ion-pairing reagent and the final pH value should be considered and carefully chosen.

According to the literature data, reverse-phase liquid chromatography (RP-LC) was the predominant technique for the determination of BAs, in which a weak solvent for the mobile phase possesses polar characteristics, whereas a strong solvent has hydrophobic properties. As the weak solvent, usually water adjusted to acidic pH with formic acid (FA) (e.g., [23,29,30]) or acetic acid [39,46] was used. Moreover, an ammonium formate (AF) buffer in water (e.g., [22,55,75]), AF or ammonium acetate (AA) buffer in water adjusted to acidic pH with FA (e.g., [16,26,51,69]) were applied as the weak solvent of the mobile phase. In the case of components of the mobile phase having a strong hydrophobic character, usually pure ACN (e.g., [58,61,75]) and ACN adjusted to acidic pH with FA or and/or AF buffer were applied (e.g., [16,29,31]). Moreover, pure MeOH (e.g., [28,52,65]) and MeOH adjusted to acidic pH with FA and/or an ammonium buffer [7,26,42,60] were also reported. Considering the pH value of the mobile phases used for the quantification of BAs in human samples, it was between 2.2 and 6.55, although values lower than 3.5 were mainly reported (Table 2). These pH values were optimal due to the decreased risk of the loss of BAs in the sample, related to their oxidation tendency. Moreover, the pH of the mobile phase decided on the ionization degree of the analytes, which is dependent on their pKa values and the pH-value of the mobile phase (Table 1). In effect, depending on the used pH of the mobile phase, the analytes can possess different charge forms, which can change their behaviour in specific chromatographic conditions. For BA determinations, more effective separations can be obtained using an acidic pH of the mobile phase.

The data summarizing chromatographic methods for BA determination confirmed that gradient elution of BAs was mostly applied, based on the controlled modification of the composition of the mobile phase during the run-time of the analysis, although the isocratic mode with a constant mobile phase composition during chromatographic separation was also reported [29,38,42,44,51,70,71]. Gradient elution allows the separation of the compounds whose peak resolution under isocratic conditions is unsuccessful. It is especially important when a lot of compounds should be separated and quantified simultaneously in the same sample during one chromatographic run analysis time. This is common for BA determinations in human samples, which explains the frequent application of this elution mode in many pharmaceutical and clinical applications. On the other hand, gradient elution, because of the modification of the mobile phase composition, can cause a change of the pH-values. In consequence, various chromatographic behaviours of the analytes can be observed during chromatographic separation. This phenomenon should be investigated during the method development.

It should also be noticed that the composition of the mobile phase, the used flow rate and the elution mode, combined with the selected stationary phase, is optimized in terms of obtaining high selectivity and sensitivity in a possible short analysis time. Of course, this task is more complicated for the simultaneous determination of many analytes with different physicochemical properties. And according to the literature data, the separations of BAs in human samples with the equilibration of the column involved an analysis time from 3.0 [23] to 40 min [39].

### 3.5. GC-MS/MS

Gas chromatography, coupled to mass spectrometry (GC-MS) or tandem mass spectrometry (GC-MS/MS), is commonly considered as a valuable tool in clinical chemistry because of high resolution, which allows even minor structural differences between analytes to be identified and high sensitivity. An advantage is also the very low sample volume required for GC analysis (usually 1–5 µL). Despite the unquestionable advantages of GC-MS(MS), this separation technique has not become widely used as a routine technique for quantifying BAs in clinical laboratories. This is related to the chemical structures of the compounds of interest, which are non-volatile and thermolabile. It means that a derivatization step is required before GC separation for producing volatile and stable adducts of BAs which are amenable to ionization techniques. The derivatization reagent should produce only one derivative, remain stable in the analysis and possess appropriate properties with regard to the used detection mode. If this process is not correctly optimized, multiple derivatives can be created, which may hinder the identification and quantification of the analytes. Unfortunately, there are a limited number of standard methods or established protocols which have been reported in the literature for the reliable derivatization of BAs. Moreover, time-consuming and labour-intensive sample preparation is required, including both extraction procedures and derivatization, as was described in part 2.2.

**Figure 9:**
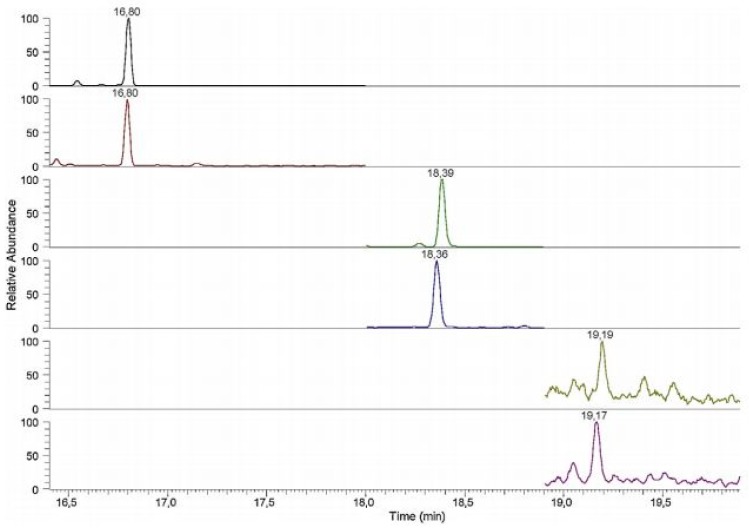
SPME-GC-QqQ-MS (SRM acquisition mode) chromatogram of a real urine sample from an healthy individual (retention times: 16.80 DA: 16.80 DA-d3; 18.39 5-HT: 18.36 5-HT-d4: 19.19 NE: 19.17 ME-d6). Figure adopted from reference [74] with the copyright permission.

Since 2010, limited numbers of GC-MS(MS) methods for the quantification of BAs in human samples have been reported (Table 3). In these methods, the analytes were mainly ionized using electron ionization (EI) [47,73,74]. The chromatograms were extracted in both SIM [33,45,47,66], SRM [74] and MRM mode [67,73]. For example, GC-EI-MS supported by SPE and derivatization was applied for the quantification of DA, HVA, L-DOPA, NM, E, NE, DOPAC, VMA, 5-HT and 5-HIAA in human urine samples. The used GC-MS conditions allowed LOQs between 0.17 and 17.84 ng/mL to be obtained for the analytes studied [66]. The same derivatizing agent was applied before the GC-EI-MS/MS analysis of many analytes, including HVA, MHPG, VMA, 5-HIAA, DA, M, L-Tryp, 5-HT, L-DOPA, DOPAC, NM, E and NE, in human urine [67]. The LOQs for the mentioned BAs were calculated from 1 to 25 ng/mL. Gas chromatography-triple quadrupole mass spectrometry (GC-QqQ-MS) for the measurement of HVA, VMA and 5-HIAA in human urine supported by derivatization and SPME was also developed [73]. The application of EI in MRM mode allowed the LOQs of 2.7, 0.063 and 49.6 µg/L to be obtained for HVA, VMA and 5-HIAA, respectively. GC-QqQ-MS technique was also used for the quantification of DA, 5-HT and NE in the urine samples [74]. The chromatogram of the real urine sample was presented in Figure 9.

However, as mentioned above, nowadays, GC-MS(MS) applications, due to their poor practicability and high requirements for operational skills and expertise have remained limited in clinical practice, whereas, in parallel, increased interest in the use of capillary electrophoresis (CE) for the determination of BAs in human samples has been noticed.

### 3.6. CE

An alternative to LC methods for the simultaneous determination of BAs in human samples are electromigration techniques as an analytical tool, thanks to their advantages, such as higher efficiency and separation resolution, simpler instrumentation, minor consumption both of the sample and chemicals and tolerance to complex mixtures. Moreover, because of their satisfactory water solubility and multiple ionization sites, they are more amenable for electrophoretic separation than chromatographic analysis. BAs have an amphoteric nature, in contrast with some of their metabolites, such as DOPAC, HVA and VMA. Their catechol group is quite stable in an acidic medium; however, it can oxidize to a quinone species both in neutral and alkaline conditions, making them electroactive compounds. It is worth noting that BAs usually have two pKa values, (pKa1 and pKa2) (Table 1) but when one of their substituents is a carboxyl group, the pKa (COOH) value increases [14]. BAs with an alkaline nature show the highest levels of electrophoretic mobility, therefore in the separation system, at normal polarization, they migrate first. Zwitterion BAs, in these circumstances, travel along the capillary a bit slower and at the last one, the window of detection, they obtain acidic amines, those with the lowest values of electrophoretic mobility.

#### 3.6.1. Electrolyte Composition

As capillary electromigration methods evolved, it became obvious that wide versatility in the selectivity of separation can be achieved by changes in the buffer composition, mode of CE technique and proper selection of the type of detector. Because most compounds of interest are protonated under physiological conditions, they are cations and thus their separations by capillary zone electrophoresis (CZE) can be carried out under acidic conditions in the presence of a small electroosmotic flow (EOF) [89,90] or weakly acidic electrolytes [91,92,93,94]. A CZE mode capable of carrying out the simultaneous separation of 10 key BAs in urine samples from healthy male athletes with 150 mM phosphate and 1 mM borax at a pH of 6.1 as the running buffer was developed [91] (Table 4).

However, at very acidic electrolytes, the acidic metabolites of BAs were separated with satisfactory efficiency but the basic analytes were not separated at low pH conditions [95]. On the other hand, separations of BAs by CZE are sometimes unsuccessful, especially because they also have low hydrophilic properties and similar dissociation constant (pKa) values, as well as the tendency to be adsorbed on the inner capillary wall. To overcome the solubility problem and improve resolving power, micellar electrokinetic chromatography (MEKC), resulting in a significant change in the migration behaviour of the BAs and their metabolites, was adopted.

An anionic surfactant, such as sodium dodecyl sulphate (SDS), that forms micelles at concentrations greater than its critical micelle concentrations was added to the running electrolyte to provide pseudostationary phases for resolving neutral or zwitterion solutes in MEKC. Negative molecules during the electrophoretic separation slightly interact with micelles and therefore migrate at first, whereas the zwitterion or neutral analytes can interact with a different intensity or even be incorporated into the interior of the micelle. This approach was reflected in the MEKC method described by Miękus et al., [96,97] employed to effectively separate seven BAs in human urine samples. In these works, the effect of BGE additives such as MeOH and α-cyclodextrin was also described.

**Figure 10:**
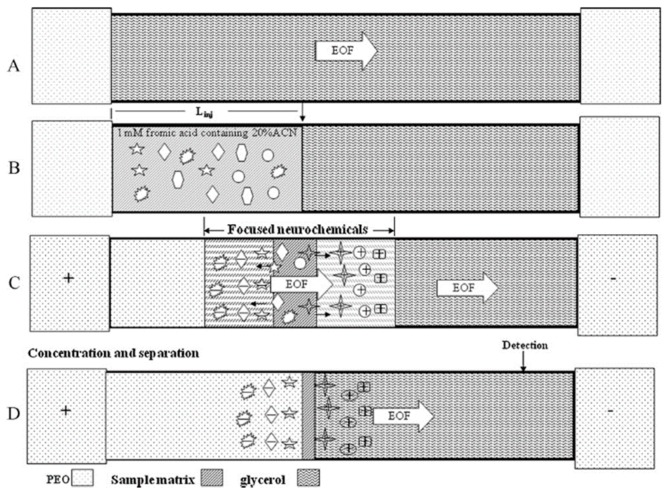
Evaluation of analyte zone in the separation and stacking of neurochemicals. (**A**) Filling of capillary with 500 mM TB (pH 9) containing different concentration of glycerol; (**B**) injection of a large volume of analytes solution; (**C**) stacking of twelve analytes by glycerol and PEO; (**D**) separation of the stacking cationic and anionic neurochemicals by glycerol and PEO. The µ_EOF_ and µ_EP_ present the EOF mobility and the electrophoretic mobilities of cationic and anionic neurochemicals, respectively. Scheme adopted from reference [98] with the copyright permission.

Due to the very low, almost trace levels of BAs in human biological fluids (serum, plasma, urine) and tissues, most of the current electromigration research focuses on improving CE detection limits and sensitivity by adopting preconcentration techniques, developing high sensitivity detectors or combining these approaches. In the last decade, the most common methods of electromigration on-line preconcentration strategies, known as field-amplified sample stacking (FASS) and field-amplified sample injection (FASI) have been employed to enhance sensitivity. The use of a high BGE concentration, the dilution of the injected sample or the addition of an organic solvent to the sample or a combination thereof will result in obtaining the FASS technique, by the hydrodynamic injection of a sample with lower conductivity than the BGE. A CE method for the simultaneous stacking of analytes by combining polymer-based and the FASS technique for selected catecholamine, indoleamine and metanephrine compounds was adopted by Hsieh et al. (Figure 10) [98].

Moreover, in the same studies, the authors used, as a supplement to Tris-borate (TB) buffer, 10% (*v*/*v*) glycerol and 0.5% polyethylene oxide (PEO). Based on the differences in the electric field between the sample zone and the TB-containing electrolyte and viscosity differences between the sample zone and the polyethylene oxide (PEO)-containing buffer, a sensitivity improvement of 116 to 281-fold was obtained, providing LODs at nM levels for cationic and anionic neurochemicals in urine samples from a healthy volunteer.

In order to increase the sensitivity of CE methods, on-line sample preconcentration based on field-amplified sample injection (FASI) was successfully used by Claude et al. [89]. This injection technique consists of stacking ionic analytes at the interface between two zones with different conductivity. Before electrokinetically injecting the sample, a low conductivity solvent is hydrodynamically introduced at the inlet of the capillary, previously filled with a high ionic strength running electrolyte. Finally, analytes are concentrated at the boundary between the pre-injection plug zone with low conductivity (high electric field) and the BGE zone with high conductivity (low electric field) [89].

Different types of on-line sample preconcentration techniques, including FASS, head-column (HC) FASS, electrostacking and sweeping were performed for the determination of A, NA, DA and NM in an N-alkyl substituted imidazolium coated capillary [90]. Moreover, in this work, it was found that long-chain imidazolium ionic liquids (C12MImCl and C16MImCl) in BGE can create a dynamic coating of the quartz capillary walls and generate anode electroosmotic flow. The elaborated method allows LODs to be obtained for selected BAs at a concentration range of 0.3–1.1 ng/mL in urine samples.

**Figure 11:**
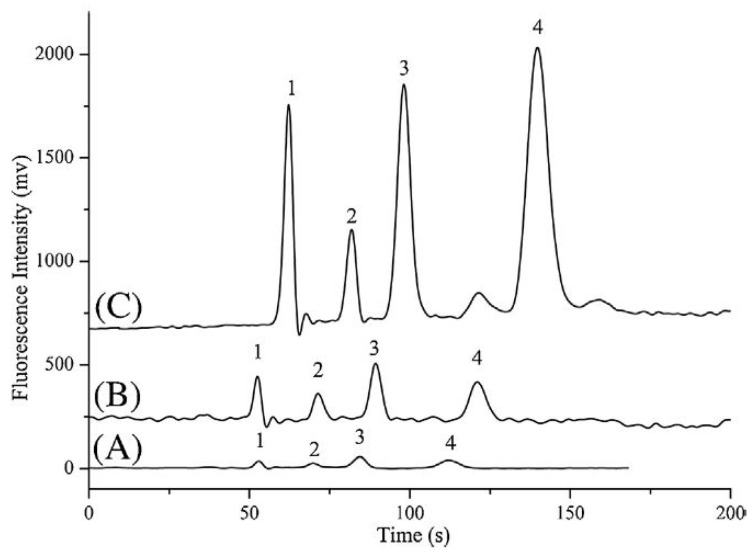
Signal enhancement of the multiple concentration: (**A**) signal intensity without concentration. The running buffer was 90 mM borate solution at pH 9.4 and the sample was diluted with the running buffer solution. The sample injection time was 2 s. (**B**) Signal intensity with FASS. The sample was prepared in a 10-fold diluted running buffer and the sample injection time was 2 s. (**C**) Signal intensity with a combination of FASS and reversed–filed stacking. The sample injection time was 10 s and the reversed polarity time was 8 s. The concentrations of 5-HT, DA and NE in (**A**) were 0.4, 0.6 and 0.7 µM, respectively. The sample concentrations in (**B**) and (**C**) were 1/10 of that in (**A**). Peak identification: 1, the excess of Cy5; 2, 5-HT; 3, DA; and 4, NE. Figure adopted from reference [99] with the copyright permission.

Recently, microchip electrophoresis (MCE), conserving the advantages of low sample consumption and high analysis speed, is increasingly being viewed as a successful alternative to CE technology for rapid analysis. Therefore, an on-line preconcentration strategy combining FASS and reversed-field stacking (RFS) was developed for the efficient and sensitive analysis of DA, NA and 5-HT in real urine samples by MCE with laser-induced fluorescence (LIF) detection [99]. The proposed multiple-preconcentration strategy greatly improved the sensitivity enhancement and surpassed other conventional analytical methods for the quantification of neurotransmitters and can yield a 182- to 292-fold increase in detection sensitivity (Figure 11).

#### 3.6.2. Detection Modes after CE Separation

Various detection modes have been employed for the quantification of BAs, although UV detection was mainly applied because of its low cost and its relatively lax requirements for sample treatment. However, due to low sensitivity, caused by the short optical path length and the low sample injection volumes, the enhancement in the signal sensitivity of detection after CE separation continues to be a hot topic, especially for the analysis of biological samples in which the analytes are present at trace levels [106]. LOD values in the nM range for BAs, using electromigration techniques, have been achieved usually by employing low-UV wavelengths (200-220 nm) and using mostly preconcentration sampling techniques during sample injection. UV detection sensitivities in CE are lower than those of HPLC and can be successfully increased by on-line pre-concentration procedures with coupling CZE with isotachophoresis (ITP). In the last decade, based on this combination a two-dimensional CE method with (2-hydroxypropyl)-β-cyclodextrin (HP-β-CD) additive in background electrolyte was developed for effective determination of 5-HT as biomarker in urine of healthy volunteers [107]. It should be noticed that ITP can serve for the sample pre-separation, elimination of sample matrix constituents (sample clean up) and preconcentration of the analytes. Despite the favourable sensitivity and the possibility of increasing the preconcentration degree of the analytes several thousand times, so far the combination of both ITP and CZE techniques has been mainly used for BA quantifications in food or beverage samples. However, this approach should be seriously considered in the nearest future also for biological matrices, including human samples.

Chemiluminescence detection (CL) has been another system employed for BA determination, principally catecholamines, due to the fact that they tend to increase the chemiluminescence formed as a result of the reaction of luminol with metal complexes in the alkaline solution. This has achieved LODs in the range of 69–100 μM for DA, E and NE in urine samples from patients with pheochromocytoma [100]. To compare the levels of E in the urine of smokers and non-smokers, a sensitive chemiluminescence (CL) system, luminol diperiodato cuprate (III), was developed by Li et al. [94]. In this case, the separation time did not exceed 7 min and the LOD for E was 0.82 ng/mL. Lower LOD values were obtained in the determination of DA and E levels in human urine from healthy volunteers using nanocrystal quantum dots in the CE buffer to catalyse the chemiluminescence reaction between luminol and hydrogen peroxide, achieving a higher CL emission [92].

Alternatively, FL detection has been a popular analytical tool due to higher sensitivity than UV detection. Since BAs do not exhibit strong fluorescence, they could not be detected directly in a sensitive manner. Although the use of laser-induced fluorescence (LIF) detection in CE provides higher sensitivity and better selectivity than UV absorbance detection, it usually requires a derivatization reagent, a compound which needs to fulfil several requirements, such as stability, minimal hydrolysis products, low reaction times and fitting in the excitation wavelengths of an argon-ion laser (351 nm and 488 nm, among others) because this is the most commonly used laser. The CE method, in conjunction with light-emitting diode-induced fluorescence detection using polyethylene oxide solutions containing SDS, has been proposed for the determination of L-Tyr, L-Trypt, NA, DA and 5-HT in human breast cancer cells (MCF-7) and human epithelial cells (H184B5F5/M10) [101]. In the presence of SDS and PEO, the adsorption of analytes on the capillary wall was suppressed, leading to high efficiency and reproducibility, achieving, in conjunction with laser-induced fluorescence or light-emitting diode-induced fluorescence (LEDIF) detection, LODs ranging from 2.06 to 19.17 nM.

Amperometric detection (AmpD) was also applied for the monitoring of BAs in CE methodologies. A dynamic pH junction technique for the CE-AmpD analysis of six BAs, including DA, E, NE, L-Tyr, L-Tryp and 5-HT has been proven to be sensitive, rapid and reproducible. Compared with the classical CE method previously reported, the method described by Tang et al. [102] showed approximately a 100-fold enhancement, which allowed μM levels of LODs to be obtained for the analytes. Zhao et al. developed another microchip electrophoresis (MCE) application with AmpD (Ag/AgCl) to determine DA, E, 5-HT and catechol in spiked CSF from healthy human samples using a microfluidic poly-dimethylsiloxane (PDMS) device with its microchannel coated with polystyrene nanosphere/polystyrene sulfonate to increase the separation efficiency and to stabilize the EOF [103].

The FASS technique using a fused silica capillary coated with gold nanoparticles (AuNPs) embedded in poly(diallyldimethylammonium) chloride (PDDA) with a boron-doped diamond electrode was investigated for the electrophoretic separation of HVA and VMA by Zhou et al. [105]. The authors used the elaborated method for the evaluation of the HVA/VMA ratio in urine samples and for the diagnosis of Menkes disease.

Although over the last decade, CE-MS systems have proven very characteristic to facilitate the unambiguous identification of complex biological samples, there are no reports in the literature about the use of such a strategy for BAs in biological samples of human origin.

### 3.7. Internal Standards

It is generally known that methods based on internal standards allow more precise and accurate quantification of analytes. This validation protocol has been commonly applied in both LC, GC and CE methods, where several compounds were used as internal standards for the quantification of BAs, depending on the applied separation technique and detection mode (Table 2, Table 3 and Table 4). For example, the LC-MS/MS method for the determination of BAs in urine and plasma samples was based on norvaline [25,48] and 3,4-dihydroxybenzylamine (DHBA) [69,70] applied as the internal standards, while 3-phenyl butyric acid was used in GC-MS/MS analysis for the quantification of HVA and VMA in urine samples [66]. However, the most common isotopically-labelled BAs were applied, particularly for the methods based on MS/MS detection, independent of whether the LC [7,16,26,28,30,31,34,41,42,43,44,49,50,53,54,55,56,57,58,59,62,63,64,65,68,76,77] or GC [33,66,67,73,74] technique was used. It is related to the fact that isotopically-labelled analytes are ionized identically to those of unlabelled compounds, which allows better accuracy and precision to be obtained. For BA electrophoretic assays, internal standards were only used for UV detector applications. The most common for this purpose 3,4-DHBA [89,95], phenyl-propanolamine [93] or 4-metoxythyramine [96] were employed.

LC, GC and CE methods without internal standards were also developed and validated. However, as mentioned above, methods based on external standards are burdened by a bigger probability of imprecise and inaccurate assay, especially for biological samples possessing complicated matrices, which have to be extensively cleaned-up and/or derivatized.

## 4. Non-separation Approaches for the Quantification of BAs

### 4.1. Immunoassays

As mentioned above, GC, LC or CE techniques belong to the most important instrumental methods for the precise and accurate determination of BAs. On the other hand, these methods can be considered as time-consuming and labour-intensive as well as requiring considerable skill. An interesting alternative with respect to this seems to be immunoassays for the quantification of BAs, which are generally simple, robust and can be fully automated giving a high sample throughput. Moreover, these methods are also commercially available in the form of test kits and relatively little technical expertise is required for use. They belong also to the most sensitive of all quantification methods [108]. Therefore, immunoassays were applied for the quantification of metanephrines in plasma and urine [109,110], while enzyme-linked immunosorbent assay (ELISA) was used to determine DA, NE, 5-HT and a sulphate derivative of dehydroepiandrosterone (DHEAS) in spot urine [111], as well as DA, NE and 5-HT in serum samples [112,113]. However, immunoassays also possess serious limitations. The most important are related with low specificity and accuracy because of possible cross-reactivity, nonspecific binding or interferences due to matrix effects, which are well known and described in the literature [114,115]. Another drawback is the necessity to use a different assay for each BA. In addition, they are able to determine the analyte in a narrow concentration range. Moreover, internal standards are not used in immunoassays, which decreases confidence in the presented values. A serious problem is also the high inter- and intra-assay variability of commercially-obtained assays, causing difficulties for long-time patient observations, especially that various assays give different results [116]. In effect, the application of immunoassays is not widely applied in clinical practice. On the other hand, they can be considered as attractive tools in small hospital-based laboratories and other institutions in which a low throughput means that a large capital outlay for instrumentation is not economically justified.

### 4.2. Electrochemical Sensors

Nowadays, electrochemical sensors are also available, designed for the recognition of BAs, which, due to their simple construction, low cost and high sensitivity, can be treated as a good alternative to traditional separation techniques. Additionally, these electrochemical sensors may be coupled to miniaturized and portable devices for specific applications in clinical and diagnostic fields. It should be noticed that electrochemical sensors are able to detect substances undergoing reduction or oxidization reactions. However, many compounds coexist in a living organism, including catecholamine metabolites, other BAs, uric acid (UA) and, especially, ascorbic acid (AA) and vitamin C, which also possess electrochemical properties. In consequence, the selectivity of BA determinations can be significantly decreased. Another critical problem of BA quantification is related with the passivation of the electrode surface because of the polymerization of the oxidation products of these substances. This also negatively influences selectivity and reproducibility (electrode poisoning or fouling). To overcome the difficulties mentioned above, many chemical modifications of the electrode surfaces were developed and reported, especially based on molecularly imprinted polymers (MIPs) [117], which enable the electrode kinetics of the compounds of interest and/or the interfering species to change. Moreover, the treatment of the electrode surface to reduce the polymerization of oxidation products and inhibit electrode passivation was also introduced. For example, glass carbon electrodes (GCE) were modified with Nafion-cucurbit [8]uril (CB[8]) and PVC-CB[8] [118], C-cundecylcalix[4]resorcinarene [119], poly(5-hydroxytryptamine) [120], electropolymerized bromothymol blue (BTB) [121], poly(L-lysine)/graphene oxide [122] and benzofuran derivative-functionalized multiwalled carbon nanotubes (MWCNT) and ionic liquid (IL) [123] for the detection of 5-HT, NE and DA in human serum and urine samples, whereas graphene (GR) with a poly 4-amino-3-hydroxy1-naphthalenesulfonic acid modified screen-printed carbon sensor was used for the simultaneous quantification of DA and 5-HT in both human urine and blood samples and pharmacological formulations [124]. The monitoring of E and NE in human blood plasma and urine samples by square wave voltammetry was also based on modified MWCNT with edge plane pyrolytic graphite electrodes (EPPGE) [125]. Another approach for DA detection in serum was with an inkjet-printed Nafion/MWCNT sensor [126] or a modified carbon paste electrode (CPE) using MWCNT-glycine [127]. Additionally, the application of an incorporation of nanomaterials (nanoparticles (NPs), carbon nanotubes (CNTs), GR, among others) on sensor devices improved sensitivity, making easier the quantification of BAs at physiological levels. For example, an electrochemical sensor using MIP oxygen-containing polypyrrole (PPy) decorated CNT composites was applied in the quantification of DA in serum and urine samples [128]. An electrochemical sensor based on modified GCE with cetyltrimethylammonium bromide (CTAB) assisted SnO_2_ NPs was developed for the simultaneous detection of E and NE in urine samples [129], while GCE/MWCNT-metal oxide electrodes with nickel, zinc and iron oxide NPs were applied for 5-HT quantification in urine samples [130]. Reduced graphene oxide (rGO)/polyaniline (PANI) nanocomposites and MIPs embedded with gold NPs (AuNPs) were also fabricated to obtain the sensitive and selective detection of 5-HT in serum samples [131]. An interesting approach is also the modification of the electrode surface with enzymes like, for example quercetin, peroxidase enzyme and metal complexes, as well as by nucleic acids, especially DNA, which are able to recognize specific molecules. Biosensors allowed an increase in the selective detection of many analytes. For example, a DNA and GR bi-layer modified carbon ionic liquid electrode (CILE) was used for the detection of DA, in the presence of excess AA, in the injection solution and human urine samples [132].

It should be noticed that dynamic progress in the development of new electrochemical sensors has recently been observed. This indicates that in the near future, biosensors, due to their short analysis time and the application of disposable transducers based on screen-printing technology produced with good reproducibility and low cost, will be commonly applied in many pharmaceutical and clinical studies. However, further progress in the development of biosensors with high selectivity and sensitivity, which will be able to reliably identify and precisely and accurately quantify analytes is still required.

## 5. Diagnostic Performance

The key requisite to ensure the high quality of diagnostic performance of each laboratory test, including catecholamine-related biomarkers, is the precise and accurate determination of the analytes. So far, MS/MS based technologies, especially LC and UPLC, are considered as analytical tools guaranteeing the best identification and quantification. On the other hand, there are many other factors which can significantly influence the quality of diagnostic tests. One of them is the choice of the best available biomarkers in terms of their selectivity for the diagnosis of specific diseases. Another crucial parameter is the comparison of the obtained results with appropriately determined reference intervals, which should be adjusted to each population, for example hypertensive, normotensive, polymedicated and polymorbid patients [68,133]. The use of adjusted reference intervals is particularly important for determinations in children [134]. The problem with using reliable reference intervals was especially highlighted in respect to the application of immunoassay kit methods.

Moreover, a lot of pharmaco-physiological factors affecting the production or clearance of determined analytes such as drugs and diet or environmental sources, including also conditions of sampling and storage, can be crucial. For example, false-positive results of NM are commonly obtained when tricyclic and other antidepressants are also used by the patient. MAO inhibitors and several α-adrenoceptor agonists, including ephedrine, pseudoephedrine and phenylephrine, can also significantly increase the concentration levels of BAs and their metabolites in the body [135]. For this reason, a thorough drug-history is mandatory for ensuring the correct interpretation of laboratory tests and in some cases, these drugs should also be withdrawn before testing [136].

Another parameter which may cause changes in the levels of circulating and urinary catecholamine-related biomarkers is diet because numerous BAs belong to constituents existing in many foods. In effect, the consumption of catecholamine-rich foods like nuts, tomatoes, potatoes and beans, bananas and fruit juices as well as cheeses and red wine can substantially increase both urinary and plasma levels of these compounds [137]. The same effect can be observed after the consumption of cocaine, amphetamines and alcoholic products, as well as after cigarette smoking [138,139,140]. For this reason, samples for the determination of selected BAs should be collected after an overnight fast to avoid false-positive diagnostic results.

Other factors, such as exercise, stress and co-existing diseases can also result in physiological processes in the body which increase the plasma and urinary levels of BAs and their metabolites. Therefore, detailed instructions should be given to patients in terms of avoiding smoking, caffeinated and alcoholic beverages, catecholamine-rich foods and strenuous physical activity for at least about 8–12 h before sample collection. 24-h urine collection is especially restricted, so patients should obtain a detailed protocol to ensure the adequate collection.

False-negative results with BA determinations are mainly related to the inappropriate time of sample collection, which should be correlated to the periodical secretion of BAs by tumours [141] or the incomplete collection of 24-h urinary specimens [142]. As it was mentioned before many times, BAs belong to very unstable compounds, especially in neutral or basic urine. In order to avoid their degradation, the urine sample should be immediately acidified to a pH below 4.0 and protected from light.

All factors described above may essentially decrease the quality of diagnostic performance based on BA-related biomarkers despite the fact that the identification and quantification of the analytes is performed by precise and accurate analytical methods.

## 6. Clinical Importance of the Determination of BAs in Human Samples as Biomarkers of Different Diseases

### 6.1. BAs as Biomarkers of Oncological Diseases

Neuroendocrine neoplasia represents a heterogeneous group of diseases and BAs or their metabolites could serve as diagnostic biomarkers [96]. On one hand, neuroendocrine tumours (NETs) could be characterized by the excessive secretion of catecholamines, which can cause symptoms such as hypertension, sweating, tachycardia and palpitations. On the other hand, NETs—like carcinoid tumours—could also secrete indoleamines such as 5-HT, whose state is unmasked when symptoms such as flushing, diarrhoea, right-sided heart disease and bronchoconstriction appear. 5-HT and its main metabolite 5-HIAA, have been discussed as advantageous for measuring in blood (or platelet-poor or platelet-rich plasma) and 24-hour urine samples (5-HIAA), as biomarkers of carcinoid syndrome of midgut origin and in patients with metastatic NETs [72,143]. In particular, 5-HT stored in platelets was proposed as the most sensitive marker for the detection of carcinoids. On the one hand, the accuracy and reproducibility of 5-HT assays have to be carefully investigated. The measurement of 5-HIAA in urine as well as serum and plasma can be used for the diagnosis and monitoring of patients with NETs where liver metastases and/or carcinoid syndrome occurred [144]. Still, the measurement of 5-HIAA needs obedience to a restricted diet to avoid false-positive diagnoses of NETs [145]. Moreover, during the analysis of a patient’s data, the presence of carcinoid heart disease, especially for metastatic patients, might be determined as well, owing to the evaluation of the monoamine level in biological samples. Studies conducted by Dobson et al. confirmed the usefulness of the 5-HIAA plasma assessment for the diagnosis of carcinoid heart disease. Those studies also confirmed that a panel of biomarkers (such as: N-terminal pro Brain Natriuretic Peptide together with 5-HIAA) should be more preferably monitored for both the sensitive and specific diagnosis of carcinoid heart disease [146]. Therefore, indoleamine biomarkers could only aid the diagnosis of tumours of neuroendocrine origin (carcinoid tumours especially mid-gut carcinoids but also other NET (pancreas or lung)) and it is preferable to consider the monitoring of multiple biomarkers at the same time [5,96].

Patient data also confirmed the utility of the measurement of plasma catecholamines—E and NE. They are routinely determined for the diagnosis of catecholamine-secreting NETs or the monitoring of the tumour’s progression. The measurements of E and NE were for a long time considered as the most appropriate biomarkers for several NET tumours. Nowadays, scientific data have indicated that preferably, metanephrines are a more precise type of biomarkers for the diagnosis of pheochromocytomas (PHE) and malignant pheochromocytomas and paragangliomas, which generally occur as benign tumours but there are few classifying systems that evaluate the risk of metastases in those neoplasms [5,50,53,55,68,147]. Even though metanephrines are considered as “gold standards” for the diagnosis of pheochromocytomas, there are patients with PHE where it is strongly recommended to determine DA—another catecholamine from the L-Tyr metabolic pathway—in the patients’ body fluids, since some tumours can secrete DA having normal E and NE levels at the same time [148]. For PHE patients, there is no need to evaluate the levels of VMA and HVA in the urine since they lack sensitivity and specificity for those tumours. However, urine metabolites of catecholamines: VMA and HVA, might be routinely tested for the diagnosis and monitoring of NETs other than pheochromocytomas (especially neuroblastoma tumours (NBL)) [43,45,58,77,96,134]. NBL is a heterogeneous disease and can arise anywhere along the sympathetic nervous system. These neural crest-derived tumours have notably different clinical behaviours. Therefore, the prognosis and treatment for NBL is highly dependent on molecular, genetic and pathological tests and biochemical biomarkers such as CATs could be of great importance to improve the diagnosis and surveillance studies. Over 92% of cases of NBL are accompanied by an increase in urinary HVA and/or VMA excretion and also DA [149,150]. Low values of urinary VMA and of VMA/HVA ratios or high values of DA/VMA and of DA/HVA ratios, may be predictive of a poor prognosis in aggressive NBL [151]. But also clinicians should be aware that urinary dopamine presented in urine samples is derived from both the renal extraction and decarboxylation of circulating 3,4-dihydroxyphenylalanine, brought by food. This is caused by the fact that NBLs overproduce E and DA and have high levels of tyrosine hydroxylase–which catalyses the initial and rate-limiting step in the biosynthesis pathway of catecholamines–but NBLs lack the storage granules for E and DA, which leads to the rapid metabolization of those BAs into VMA and HVA [152]. The specificity of catecholamines determined in the NET patients’ urine samples is excellent and approaches 100% in some papers, whereas it drops below 85% in other papers [153]. Still, several countries implement, in their population-based screening programs, the screening of CATs in NET patients’ urine samples. Preferably, 24-hour urine collection is taken into account but some scientific data demonstrated the high correlation between 24-h collections and random or “spot” urine samples, as well. Furthermore, catecholamine metabolites might be useful to verify if a patient suffers from NBL or Wilms’ tumour, since both of them are frequent paediatric solid tumours, both localized in the upper abdominal quadrant [153,154,155]. This discrimination between them could be difficult for clinicians. The addition of urinary free NM with either VMA or HVA significantly enhances the diagnostic sensitivity of the panel of biomarkers [84]. NM, M, HVA, VMA and other catecholamine elevations were observed in neuroblastic tumours, including NBL, ganglioneuroblastoma and ganglioneuroma [156]. Nevertheless, numerous medicines can interfere with urine catecholamine analysis risking a false-positive diagnosis, such as the usage of tricyclic antidepressants, which can cause false-positive M and NM results in both plasma and urine or L-DOPA therapy. In the latter therapy, scientific data revealed that the urinary excretion of DA and HVA is elevated in adult patients treated with L-DOPA for Parkinson’s Disease, which could hinder the diagnosis of NBL. In such cases, it is more appropriate to determine a panel of biomarkers: M, NM, DA, HVA and VMA, instead of VMA/HVA alone [84]. Conventionally, twenty-four hour (24-h) urine samples were used for the analysis of those metabolites but random urine was discussed as advantageous, as well [157].

### 6.2. BAs as Potential Biomarkers of Cardiovascular Diseases

For many diseases of the central nervous system (CNS), such as depression, neurodegenerative diseases (Alzheimer’s Disease (AD), Parkinson’s Disease (PD)) and neurological diseases, a relationship between the level of neurotransmitters and the development and/or progression of the disease has been proven. In addition, the aging of the body contributes to the deepening of changes in the secretion of the most important BAs and hormones, which deepens changes and at the same time, has a significant impact on the development of other diseases, such as cardiovascular disease.

Researchers stress that depression as a heart disease factor in young people is more important than other traditional factors such as smoking, hypertension, obesity and diabetes. Many previous studies of depression and heart disease have affected older patients, who are usually burdened with more heart disease and are often affected by other diseases. Although there are few reports in the available literature regarding the assessment of the relationship between the level of selected neurotransmitters and cardiovascular diseases, a few studies confirm this relationship. For example, the results obtained by Hervet et al. suggest that stress situations result in a massive release of catecholamines, which can be examined after death by E and NE measurements in post-mortem serum, vitreous humor and urine, as well as in pericardial fluid and CSF [158]. Increased serum concentrations of both E and NE have been reported in myocardial infarction deaths [159]. Bessonova et al. showed that high E levels can be caused by high stress and pheochromocytoma of the adrenal gland and are often associated with hypertension and heart disease [160].

Many authors argue that NE increases blood pressure and blood glucose levels, as the hormone, circulating freely in the blood, narrows the peripheral vessels, while at the same time expanding coronary vessels in the heart [10,161,162]. NE is also suspected of playing a role in the development of post-traumatic stress disorder (PTSD) [163].

DA at higher concentrations is also associated with β-adrenergic receptors, accelerating cardiac output and increasing the ejection volume, as well as α-adrenergics, the stimulation of which causes vasoconstriction and increases resistance in peripheral vessels [161,164]. In turn, a study conducted by Zhang et al. concerned the DA levels in plasma samples from neonates suffering from hypotension [54]. Hypotension is common in preterm infants. It very often results in serious problems, including cardiovascular compromise, some neurodevelopmental sequelae and an increased incidence of mortality. However, according to the authors, the causes of neonatal hypotension still remain unclear. It is known that DA plays a significant role in maintaining cardiovascular homeostasis and is the most frequently prescribed medication for neonatal hypotension because of its inotropic and vasoconstrictive actions. For this reason, it is important to measure baseline and therapeutic DA levels for the diagnosis and treatment of hypotension in preterm infants.

The level of DHPG in plasma and the ratio of NE to DHPG concentrations are markedly increased in patients with heart failure and patients with pheochromocytoma. In healthy people, the level of both free DHPG and bound DHPG increases markedly with age. Therapeutic use of (S) -DHPG is possible to maintain the normal blood pressure and heart rate during anaphylactic shock and to treat memory disorders associated with ischemia and hypoxia [165]. It was also confirmed that in patients with known hypertension, the urinary VMA concentrations were more than 7 times higher, while in patients with pheochromocytoma of the adrenal glands, they were determined to be over 35 times higher than in healthy people. Among patients with depression, the level of the urinary excretion of VMA significantly correlates with plasma cortisol with respect to the administered dexamethasone [166].

The determination of NM in the urine is useful in the diagnosis of hypertension [167], whereas the determination of free M levels in the serum is the best tool for diagnosing pheochromocytoma of the adrenal medulla and other neuroendocrine tumours, especially inherited forms and helps in the diagnosis of hypertension [168].

### 6.3. BAs as Potential Biomarkers of Neurodegenerative and Psychiatric Diseases

Numerous associations between the levels of selected BAs in human biological fluids and disease states, including neurodegenerative and psychiatric diseases, have been documented in the scientific literature and can provide valuable information as part of a comprehensive health assessment. BA concentrations in real human samples can vary considerably over the course of a day. Moreover, as it was earlier mentioned, high blood BA levels can be induced both from environmental or physiological stress, such as low blood glucose levels, work stress, exercise, sleep deprivation, as well as high levels of noise. Likewise, their fluctuating concentrations are also affected by the diurnal cycle, with most catecholamine levels dropping during sleep but rising significantly after waking up [169].

BAs can be implicated in several affective behaviours, such as euphoria, depression and anxiety. These responses provide certain important biological functions and can become disordered in such a manner to constitute an illness, generally referred to as mood disorders. The intensification of the serotonergic and noradrenergic tone in the brain is commonly associated with depression, resulting in a lack of the behavioural effects of euphoria and pleasure. In patients with this disorder, low circulating levels of catecholamines, especially 5-HT, have been identified. The dysregulation of 5-HT systems has been implicated in depression, anxiety disorders and schizophrenia [170]. Other neuromodulator transmitters also play a major role in theories of psychiatric diseases, such as acetylcholine for schizophrenia [171], DA, NE and 5-HT for attention-deficit/hyperactivity disorder (ADHD) [172,173]. Moriarty et al. [62] determined the levels of 5-HT, 5-HIAA and DA in urine samples from a control group and children diagnosed with ADHD, to evaluate their usefulness as potential markers for diagnosis and for monitoring the efficacy of treatment. The obtained results confirmed that only 5-HIAA levels were significantly different in the patients with ADHD compared to the control population. However, extended studies including larger groups of patients and controls are required for checking the reliability of the application of 5-HIAA as a biomarker of ADHD.

Moreover, relatively high levels of monoamine transmission, especially DA and 5-HT, can be a characteristic of psychosis, which can also occur as a long-term side effect of several stimulatory psychotropic drugs [174]. Anxiety disorders and schizophrenia are also strongly associated with altered concentrations of BAs in biological fluids. The underlying biochemistry of the major neuropsychiatric disorders is often not completely understood and the therapeutic effects of antidepressants and antipsychotics have mostly provided valuable information about the neurochemical functions of BAs in these disorders. In fact, 5-HT alone is administered as an antidepressant in some cases of depression [175].

Neurodegenerative diseases also result in either an excess or deficit in monoamine availability. Among them, most notable are PD, AD and Huntington’s Disease (APHD) [176]. Clinical studies of the urinary excretion of catecholamines in patients with PD were conducted by Guimaraes et al. [177]. In this study, the determination of monoamine levels and of the DA/L-DOPA ratio, as an indirect measure of DA synthesis, the DOPAC/DA ratio, as an index of central and peripheral DA metabolism and the 3-MT/DA ratio, as an index of the central DA metabolism, was carried out in order to define a profile for the dopaminergic system. PD is characterized by the loss of dopaminergic neurons in the substantia nigra, which leads to decreased levels of DA in the striatum and disrupted motor control. In turn, AD is a chronic progression characterized by loss of memory and cognitive deficits such as agnosia, aphasia and apraxia, among others, facts that cause interference in daily life and in the individual’s work. Subsequent studies have shown that abnormal NE concentrations are associated with stress, anxiety, depression, AD and PD [178].

Of note, it should be kept in mind that some psychiatric diseases may also be associated with a modified 5-HT metabolism. For instance, some autistic subjects display mildly increased 5-HIAA excretion. In psychotic depressed subjects, 5-HIAA excretion was linked to insomnia. Various studies of patients with depression reported differences in 5-HIAA concentrations in CSF although they did not report urinary 5-HIAA levels; however, its concentrations in CSF, serum or urine samples are not modified by schizophrenia [179].

### 6.4. BAs as Potential Biomarkers of Endocrinal, Autoimmunological and Other Diseases

Since catecholamines such as E and NE could play an important role in the endocrine system, their determination in the sera samples of Hashimoto’s patients was carried out. It was revealed that serum catecholamine levels statistically differed in Hashimoto’s thyroiditis patients when compared to healthy subjects. Further case studies revealed that plasma levels of HVA and MHPG could be possible biomarkers for psychotic symptoms among patients diagnosed with Hashimoto’s encephalopathy (HE) (which is believed to be an immune-mediated disorder associated with Hashimoto’s thyroiditis) [180]. A study conducted by Pilotto et al. in 2019 presented strong evidence that BA deficits correlate with specific brain atrophy patterns in adults diagnosed with phenylketonuria and that the serotonergic axis is more endangered by high phenylalanine concentrations than the dopaminergic axis but both 5-HT and DA deficits are common and more heterogeneous in phenylketonuria patients than it was assumed in previous research [181]. Vitiligo is a common pigmentary disorder with complex pathogenesis where biochemical and immunological factors were proposed as important. A study conducted by Basnet et al. [182] suggested that for a better selection of patients for melanocyte transfer, the determination of DA in the 24-h urine collection could be helpful. However, the authors were encouraged to investigate further for the explanation and mechanism underlying disturbances in the levels of plasma and urinary monomines or their metabolites in patients with vitiligo.

In the literature, there are also data indicating that specific BAs can be used as biomarkers for the diagnosis and therapy of addiction illnesses [183]. Saracino et al. [71] measured endogenous levels of E, NE and DA in DPSs and DUSs samples from healthy volunteers, psychiatric patients and poly-drug (e.g., cannabis, ketamine, cocaine) abusers. The concentration levels of all analytes were higher in poly-drug abusers than healthy volunteers, especially for DA. Unfortunately, no evaluation on whether these differences were statistically significant was reported.

Marcos et al. [7] measured urinary levels of 5-HT and 5-HIAA in spot urine samples from three patients from a family with Brunner syndrome. It is a rare X-linked genetic disorder caused by a mutation in the MAO-A gene, which leads to an excess of monoamines in the brain. The authors compared the obtained results for the three patients to spot urine samples collected from a control group. The data confirmed the accumulation of 5-HT in all three patients, which gave an abnormally high ratio of 5-HT in respect to its precursor (L-Tryp). However, too low a conversion of 5-HT to 5-HIAA also led to an abnormal 5-HT/5-HIAA ratio. The disturbance of the VMA, HVA and 5-HIAA and serotonin metabolisms, which were two times or higher for 5-HT and NM, respectively, as well as higher for other catecholamine metabolites in blood and urine from these patients, was also confirmed. These experimental data were correctly correlated to the metabolic abnormality of the X-linked genotype.

L-Tryp is an important biogenic acid whose catabolism in mammalians is mainly based on three pathways: the kynurenine and serotonin pathways, as well as bacterial degradation (Figure 1). As it was mentioned, most of the produced catabolites are biologically active and take part in the pathogenesis of many disease processes. For example, the kynurenine pathway plays an important role in renal physiology. It was confirmed that the blood level of L-Tryp is decreased while KYN is increased in patients with chronic kidney disease (CKD). Therefore, the quantification of L-Tryp and KYN levels in blood could be used as early biomarkers for CKD patients [184]. On the other hand, the ratio of KYN/L-Tryp is considered as a more sensitive indicator of renal function than KYN or L-Tryp alone because two parameters changing in opposite directions are measured in pathological conditions [185]. To evaluate renal function in hypertensive patients, with non-CKD and CKD groups, the levels of L-Tryp, KYN and uric acid (UA) as well as creatinine (Cr), being the most important indicator for the determination of the estimated glomerular filtration rate (eGFR), were measured [32]. The obtained data confirmed significantly higher levels of Cr and UA in patients with CKD than in non-CKD. The patients with CKD also had significantly lower concentrations of L-Tryp and KYN, whereas KYN/L-Tryp was significantly higher in CKD than in non-CKD patients. It confirms that the determination of L-Tryp and its selected metabolites, like KNY and UA, can be treated as a useful tool for the detection of chronic kidney disease, even at an early stage.

## 7. Conclusions

Biogenic amines play an important role throughout the body, both in the central nervous system and in the periphery. In effect, the disturbance of their metabolism or action may result in devastating effects on the homeostasis of the human body. On the other hand, the identification and quantification of the changed BA profiles in the human body may be considered as reliable biomarkers of many disorders, such as neuroendocrine tumours, schizophrenia and Alzheimer’s and Parkinson’s diseases. On the other hand, the determination of BA profiles, in terms of improving diagnostics and monitoring the therapy of various diseases, is a demanding task because many factors can influence false-positive or false-negative results. Therefore, diet, interactions with co-existent drugs, the health status of the patient, stress, physical exercise, sample collection and storage and reference intervals should be taken into account. Moreover, the qualification and quantification of the compounds of interest in complex biological matrices should be performed using a reliable, sensitive and selective analytical method. For years, different separation techniques like conventional HPLC, LC-MS(MS), UPLC-MS(MS), GC and CE, as well as non-separation approaches based on immunoassays and electrochemical sensors, have been developed for the precise and accurate determination of BA profiles in different human samples. However, each of these techniques has both advantages and disadvantages which should be critically evaluated by analysts with respect to the main purpose of the conducted research. The literature data published since 2010 have clearly indicated that LC-based MS techniques have a predominant position in BA investigations due to high selectivity and sensitivity. Moreover, UPLC-MS(MS), offering the effective separation of analytes in a shorter analysis time in respect to LC and CE methods, supported by new preconcentration techniques, strengthened its position in pharmaceutical and clinical laboratories. In many studies, the need for the simultaneous detection of many BAs in complex biological matrices at very low concentration levels requires the application of effective sample pre-treatment, including extraction and/or derivatizing protocols. In this field, we have systematically observed progress in the development of new sample preparation procedures and chromatographic/electrophoretic methodologies in order to guarantee simple, fast and robust detection and quantification of BAs in human samples. In the near future, electrochemical sensors, because of low cost, simplicity and high throughput, will probably play an important role in BA level diagnosis, although further development and validation is still required in order to enhance their capabilities for effective application in clinical diagnostic laboratories. Moreover, the number of publications reporting the determination of BAs as biomarkers of various diseases have systematically increased, which has allowed a deeper understanding of the physiological and pathological mechanisms of BAs to increase the effectiveness of oncological, endocrinal, cardiac or neurodegenerative therapies.

## Figures and Tables

**Table 1:** Classification of selected BAs on the basis on physicochemical parameters.

Analyte	Chemical Structure	pKa Values	Log P (Experimental)	Water Solubility	
				Experimental	Predicted
BAs of an alkaline nature					
DA	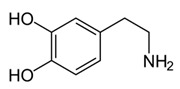	9.27 10.01	−0.98 E	535 ng/mL	7.43 g/L
E	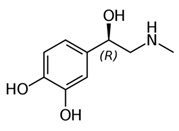	8.91 9.69	−1.37	0.18 mg/mL	18.6 g/L
NE	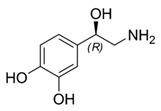	8.85 9.5	−1.24 E −1.4	849 mg/mL	12.5 g/L
L-Tryp	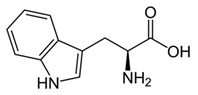	2.54 9.4	−1.06	13.4 mg/mL	1.36 g/L
L-Tyr	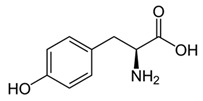	2 9.19	−2.26	0.48 mg/mL	7.67 g/L
Zwitterion BAs					
5-HT	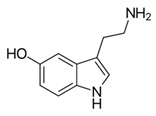	9.31 10	0.21	25.5 mg/mL	2.5 g/L
L-DOPA	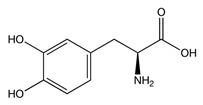	1.65 9.06	−2.39	5 mg/mL	3.3 g/L
DHPG	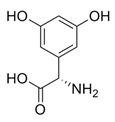	−3 9.21	−1.01	---	16.7 g/L
MHPG	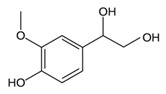	−3 9.91	−0.13	---	8.99 g/L
NM	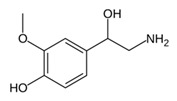	9.06 9.99	−1.05	---	8.44 g/L
M	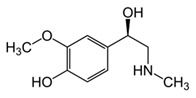	9.25 10.05	0.4	---	13 g/L
BAs with the acidic nature					
VMA	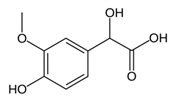	−4.1 3.11	0.43 0.94	---	5.16 g/L
HVA	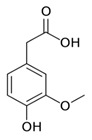	−2.2 −4.9	−0.75 0.67	---	1.34 g/L sulphate
DOPAC	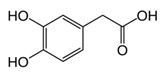	−6.3 3.61	0.98	4 mg/mL	7.23 g/L

**Table 2:** LC methods for the separation of selected BAs in human specimens published since 2010.

Analytes	Human Sample/Volume	Sample Preparation	LC Conditions			Validation			Internal Standard (IS); Analysis Time /Time with Equilibration	Ref.
			Solid Phase	Elution Mode; Mobile Phase	Detector	LOD/LOQ	Linearity Range	Absolute Recovery (%)		
NE, 5-HT, 5-HIAA, DA, DOPAC, HVA	CSF	Derivatization with benzylamine and DPE, except of HVA	For HVA: Atlantis C18 column (2.1 × 50 mm, 3 µm) Other analytes: Acquity UPLC BEH Shield C18 column (2.1 × 100 mm, 1.7 µm)	Isocratic elution: For HVA: 75 mmol/L NaAc buffer, pH 5.0 Other analytes: 15 mmol/L acetate buffer (pH 4.5) in ACN with 1 mmol/L octanesulfonic acid sodium salt	HPLC-FD (λ_ex/em_) For HVA: 275/345 nm, Others: 345/480 nm	LOD: 50 pmol/L LOQ: n.d.	n.d.	n.d.	n.d. HVA–10 min, other analytes: 8 min	[3]
L-Tryp and 8 of its metabolites, DA, HVA and others	Urine 100 μL Plasma 150 μL	Urine: Dilution with water Plasma: Deproteinization with ACN	Acquity BEHC18 column (2.1 × 100 mm, 1.7 µm)	Gradient elution: A: 0.01% FA in wate B: 0.01% FA/1 mM AF in water B: 0.01% FA/1 mM AF in MeOH	LC-MS/MS	Urine: LOD: 1–100 ng/mL LOQ: 5–1000 ng/mL Plasma: LOD: 1–100 ng/mL LOQ: 2–1000 ng/mL	Urine: From 10 to 25000 ng/mL Plasma: From 2 to 30000 ng/mL	n.d.	The deuterated ISs; 9.5/12.0 min	[7]
5-HT, NE, E, 5-HIAA, M, NM	CSF 800 μL plasma 400 μL	SPE-STRATA CW Derivatization by reductive diethylation	Synergi-Fusion-RP C18 column (5.0 × 150 mm, 4 µm)	Gradient elution: A: 0.2% FA in 10 mM AF, pH 3.5 B: 0.1% FA in ACN	LC-MS/MS	In CSF: LOD: n.d LOQ: 0.012 ng/mL In plasma: LOD: n.d. LOQ: 0.018 ng/mL for all analytes except of 5-HT–12 ng/mL	In CSF and plasma: From 0.012 to 140 ng/mL	n.d.	The deuterated ISs; 3.6/4.0 min	[16]
7 Analytes including HVA	Urine 50 μL	Dilution with solvent A of the mobile phase	ZORBAX Eclipse Plus C18 Rapid Resolution column (4.6 × 100 mm, n.d.)	Gradient elution: A: 50 mM AF and 5 mM octanesulfonic acid in 99% water and 1% MeOH, pH 6.55 B: 50 mM AF and 5 mM octanesulfonic acid in 100% MeOH, pH 6.55	HPLC-DAD 233 nm	LOD: n.d. LOQ: 45 mg/mL for HVA	45–1000 mg/L	n.d	External method; 16.5/25 min	[22]
5-HIAA	Urine 50 μL	Automated procedure: centrifugation and injection into LC	Kinetex XB-C18 column (2.1 × 50 mm, 1.7 μm)	Gradient elution: A: 0.05% FA in water B: 0.05% FA in MeOH	LC-MS/MS	LOD: n.d. LOQ: 0.5 mg/L	0.5-100 mg/L	n.d.	The deuterated ISs; 2.5/3.0 min	[23]
L-Tryp and 3 of its metabolites, Cr	Urine 200 μL	Centrifugation and dilution with water	Agilent HC-C18 (2) column (4.6 × 250 mm, 5 µm)	Gradient elution: A: 20 mmol/L NaAc, 30 mmol/L HAc and 3% MeOH B: 20 mmol/L NaAc/HAc, 10% MOH and 10% ACN	HPLC-FD (λ_ex/em_) 295/340 nm	LOD: 0.02 µmol/L for L-Tryp, 0.01 µmol/L for 5-HIAA LOQ: 10 µmol/L for L-Tryp, 5 µmol/L for 5-HIAA	10-100 µmol/L for L-Tryp 5-50 µmol/L for 5-HIAA	96.1–105.2	External method; 25/30 min	[24]
DA, NE, E, 5-HT, L-Tyr, L-Tryp, DOPAC, HVA	Plasma 250 μL	DLLME with dichloromethane/ethanol as extraction/disperser solvent	XBridge Amide™ BEH column (3.0 × 100 mm, 3.5 µm)	Gradient elution: A: 10 mM AF in water, pH 3.0 B: 10 mM AF in ACN	LC-MS/MS	LOD: 3 and 10 ng/mL LOQ: 10 and 30 ng/mL	10–500 and 30–500 ng/mL	76.4–99.3	Norvaline; 12.0/20.0 min	[25]
5-HIAA	Plasma 50 μL	Deproteinization with ACN	SIELC Primesep B mixed-mode column (3.2 × 50 mm, 5 µm)	Gradient elution: A: 0.1% FA/10 mmol/L AA in water B: 0.1% FA/2 mmol/L AA in MeOH	LC-MS/MS	LOD: 5 nmol/L LOQ: 15 nmol/L	15–10000 nmol/L	97–113	The deuterated IS; 3.0/7.9 min	[26]
DA, DOPAC, HVA, NE, VMA MHPG, 5-HT, 5-HIAA and others	Plasma 0.3 mL	Deproteinization with ice-cold ACN; Derivatization with dansyl chloride	Phenomenex Gemini C18 column (2 × 150 mm, 3 µm)	Gradient elution: A: 20 mM AA and 0.1% FA in water B: ACN	LC-MS/MS	LOD: n.d. LOQ: 0.27–1.62 pmol/mL	From 0.8 to up 326 pmol/mL	n.d.	L-aspartic acid-15N (L-Asp-15N); 23/27 min	[27]
E, NE	Plasma 25 μL	Fully automated deproteinization with ACN and reductive ethylation using 96-well plate	ACQUITY UPLCTM BEH phenyl column (2.1 × 100 mm, 1.7 µm)	Gradient elution: A: 0.2% FA/20 mM AA in water B: MeOH	UPLC-MS/MS	LOD: 0.02 ng/mL LOQ: 0.05 ng/mL	0.05–25.0 ng/mL for E and NE	n.d.	The deuterated ISs; 3.0/3.5 min	[28]
VMA, HVA, HMPG sulphate	Urine and serum 500 μL	Urine: Dilution with water Serum: Dilution with water and deproteinization with ACN	Waters Xbridge C18 column (2.1 × 150 mm, n.d.)	Isocratic elution: 2 mM AF in ACN: 0.05% FA in water (15:85, *v*/*v*)	LC-MS/MS	LOD: 0.2 ng/mL for VMA, 0.7 ng/mL for HVA LOQ: 1 ng/mL for both analytes	1–1000 ng/mL	90.6–101.6	The external method; 5.5 min	[29]
L-Tryp and 8 of its metabolites	Plasma 190 μL	Deproteinization with MeOH containing zinc sulphate and 0.05% TCA	Kinetex C18 column (2.1 × 100 mm, 5 µm)	Gradient elution: A: 0.1% FA in water B: 0.1% FA in ACN	LC-MS/MS	LOD: 2 nmol/L for 5-HT, 83 nmol/L for L-Tryp LOQ: 4 nmol/L for 5-HT, 139 nmol/L for L-Tryp	4-600 nmol/L for 5HT, 139–400 μmol/L for L-Tryp	37–104	The deuterated ISs; 10.0/15.0 min	[30]
L-Tryp and 18 of its metabolites, including 5-HT and 5-HIAA	Serum and cell culture supernatants 50 μL Urine 10 μL	Serum and CCS: Dilution with 0.1% FA and deproteinization with MeOH Urine: Dilution with 0.1% FA and centrifugation	An Atlantis T3 RP column (2.1 × 150 mm, 3 μm)	Gradient elution: A: 0.1% FA in water B: 0.1% FA in ACN	LC-MS/MS	LOD: 0.1–50 nM LOQ: 0.5–100 nM	1–20 μM for the majority of analytes and up to 200 μM for L-Tryp	60–130	The deuterated ISs; 12.0/15.0 min	[31]
L-Tryp and others	Plasma 100 μL	Deproteinization with 0.6 M perchloric acid	Agilent HC-C18 (2) column (4.6 × 250 mm, 5 μm)	Gradient elution: A: 20 mmol/L NaAc, 30 mmol/L HAc and 30 mL/L ACN B: 20 mmol/L NaAc, 30 mmol/L HAc, 100 mL/L ACN	HPLC-UV 280 nm for Tryp	LOD: 1 μmol/ L for L-Tryp LOQ: 3.3 μmol/L for L-Tryp	10–140 μmol/L for L-Tryp	92.6 for L-Tryp	External method 15.0/25.0 min	[32]
VMA	Urine 1 mL/50 μL for further analysis	Centrifugation	Luna PFP column (2.0 × 100 mm, 3 µm)	Gradient elution: A: water B: MeOH	LC-MS/MS	LOD: 0.025 µmol/L LOQ: 0.125 µmol/L	1.0–250.0 µmol/L	85–101	d3-VMA; 2.0/4.0 min	[34]
DA, E, NE, M and MN, L-Tyr	Serum 25 µL	Evaporation and derivatization with PITC	Zorbax Eclipse XDB C18 column (3.0 × 100 mm, 3.5 µm)	Gradient elution: A: 0.2% FA in water B: 0.2% FA in ACN	LC-MS/MS	LOD: 0.00444–0.118 nM LOQ: 0.0148–0.393 nM	From 0.025 to up 100 nM	93.2–113	The deuterated ISs;7.0/9.5 min	[35]
L-DOPA, DA, NE, E, M, L-Tryp, 5-HTryp, 5-HT	Urine 10 μL	Configuration, filtration and derivatization with PFOEI	Fluofix-II 120E column (4.6 × 250 mm, 5 µm) XBridge TM C18 column (4.6 × 150 mm, 5 µm)	Gradient elution: MeOH:water:TFA (60:40:0.05, *v*/*v*/*v*) and MeOH:water:TFA (2.5:97.5:0.05, *v*/*v*/*v*)	LC-FL λ_ex/em_ = 280/ 320 nm	LOD: 0.21–4.2 nmol/mL LOQ: 1–10 nmol/mL dependent to the analyte	10–1000 nmol/mL for DOPA; 5–250 nmol/mL for DA, NE, M; 1–100 nmol/mL for E, Trp, 5-HTrp, 5-HT	96.9–103.3	External method; 16.0/20.0 min	[37]
5-HT, 5-HIAA and 3 others	Plasma 70 μL	Deproteinization with THF and derivatization with HFBA	Fluorousphase Fluofix-II 120E column (4.6 × 150 mm, 5 µm)	Isocratic elution: MeOH:water:ACN:TFA (80:13:7:0.1, *v*/*v*/*v*)	LC-FD λ_ex/em_ = 340/ 452 nm	LOD: n.d. LOQ: 4.3 pmol/mL for 5-HT 1.5 pmol/mL for 5-HIAA	4.3–300 pmol/mL for 5-HT; 1.5–300 pmol/mL for 5-HIAA	72.2–84.2	External method; 30.0 min	[38]
L-Tryp, KYN	Serum 10 μL	Deproteinization with ACN and derivatization with DBD-PyNCS	TSKgel ODS-100 V column (2.0 × 250 mm, 5 μm)	Gradient elution: A: H_2_O/ACN (80:20) with 0.1% HAc B: ACN/H_2_O (80:20) with 0.1% HAc	LC-MS	LOD: 150 nM LOQ: 0.5 μM	10-200 μM for L-Tryp; 0.5–5.0 μM for KYN	n.d.	7-Amino-heptanoic acid; 40 min	[39]
NE, E, DA, NM, M, 3-MT	Urine 50 μL	Dilution with 1.0% FA in water and derivatization with acetaldehyde	Atlantis T3 column (2.1 × 150 mm, 3 µm)	Gradient elution: A: 2% FA in water B: ACN	LC-MS/MS	LOD: n.d LOQ n.d.	n.d.	67–78	The deuterated ISs; 4.3/6.2 min	[41]
5-HT, DA, NE and others	Carcinoma stem cell	On-line MD system	Merck ZIC-HILIC column (2.1 × 100 mm, 3 µm)	Isocratic elution: MeOH: 20 mM AF in water, pH 3.0 (55:45, *v*/*v*)	LC-MS/MS	LOD: 2 pg for 5-HT, 10 pg for other analytes LOQ: 5 pg for 5-HT, 20 pg for other analytes	5–500 ng/mL for 5-HT 20–2000 ng/mL for others	n.d	The deuterated ISs; n.d.	[42]
VMA, HVA	Serum 0.5 mL	LLE with ethyl acetate	Waters Acquity UPLC HSS T3 column, (2.1 × 150 mm, 1.8 μm)	Gradient elution: A: 0.1% FA in water B: 0.1% FA in ACN	UPLC-MS/MS	LOD: n.d. LOQ: 0.02 ng/ml for VMA, 0.18 ng/mL for HVA	2–1000 ng/mL	110.5 for VMA, 108.0 for HVA	d3-VMA and 3C6,18O-HVA; 3.3/8.0 min	[43]
E, NE, DA, M, NM	Urine 1 mL	LLE with ethyl acetate	BDS Hypersil C18 column (3.0 × 125 mm, 3 μm)	Isocratic elution: Water:MeOH (98:2) with 0.25 % FA	LC-MS/MS	LOD: 0.3–3.6 µg/L LOQ: 1.0–11.1 µg/L	From 3 to 2130 µg/L	92.1–108.8	The deuterated ISs; 10 min	[44]
E, DA, 3-MT, NE, 5-HT, L-Tryp, L-Tyr and others	Urine 50 μL	LLE with TCA	Acclaim Mixed Mode WCX column (2.1 × 150 mm, 3 μm)	Gradient elution: A: 0.1 % HAc in MeOH/H_2_O (20/80, *v*/*v*) B: 2.0% HAc in MeOH/H_2_O 20/80 (*v*/*v*)	LC-MS/MS	LOD: 0.3–6.6 μg/L LOQ: 1.0–21.9 μg/L	From 1 to 2000 μg/L	72.9–100.0	External method; 10.0/20.0 min	[46]
L-Tyr, L-Tryp, 5-HT, DA, E, NE, 3-MT, 5-HIAA, VMA, HVA, L-DOPA, DOPAC	Urine 980 μL	DLLME with dichloromethane/ethanol as extraction/disperser solvent	XBridge Amide TMBEH column (3.0 × 100 mm, 3.5 µm)	Gradient elution: A: 10 mM AF in water, pH 3.0 B: 10 mM AF in ACN	LC-MS/MS	LOD: 5 and 10 ng/mL LOQ: 10 and 20 ng/mL	10–2000 and 20–2000 ng/mL	99.0	Norvaline; 12.0/20.0 min	[48]
5-HT, 5-HIAA and others	Serum 500 µL	DLLME and derivatization with benzylamine/potassium hexacyanoferrate(III)	A reversed-phase XBridge™ Shield RP18 column (4.6 × 150 mm, 5 µm)	Gradient elution: A: 250 mM acetate buffer (pH 4.3):water:ACN (50:40:10, *v*/*v*) B: 250 mM acetate buffer (pH 4.3):ACN (50:50, *v*/*v*)	HPLC-FD (λ_ex/em_) 345/452 nm	LOD: 0.08–0.33 nM LOQ: 05 nM	0.5–100 nM	66–98	The deuterated ISs; 2.5/5.5 min	[49]
Free E, NE, DA, M, NM	Urine 0.1 mL	SPE with Strata-X-CW	Unison UK-C18 column (2.0 × 100 mm, 3 µm)	Gradient elution: A: water with FA (99.9:0.1, *v*/*v*) B: ACN with FA (99.9:0.1, *v*/*v*)	LC-MS/MS	LOD: n.d. LOQ: 3.5–7.4 µg/L	From 3.5 to 2569 pmol/mL	61–107	The deuterated ISs; 2.6/n.d.	[50]
DA, E, 5-HT, L-Tryp and 2 others	Whole blood 100 μL	Deproteinization with 0.1% FA in cold ACN, automated filtration and *on-line* SPE-HILIC	HILIC column n.d.	Isocratic elution: ACN:100 mM AF at pH 70:30, *v*/*v*)	LC-MS	LOD: 1 nM for DA and 5-HT, 0.2 nM for E, 30 nM for L-Tryp; LOQ: 0.05 nM for DA and E; 5 nM for 5-HT; 250 nM for L-Tryp	0.05-50 nM for DA and E; 5–5000 nM for 5-HT; 250–250000 nM for L-Tryp	33–91	External method; 10/12 min	[51]
E, NE	Plasma 0.5 mL	Alumina B 96-well SPE; Derivatization with d4-acetaldehyde	Onyx Monolith C18 column (3.0 × 100 mm, n.d.)	Gradient elution: A: 10 mM AF in water B: MeOH	LC-MS/MS	LOD: 0.5 pg/mL for E and NE; LOQ: 5 pg/mL for E, 20.0 pg/mL for NE	5.0-500 pg/mL for E; 20.0–2000 pg/mL for NE	>66	The deuterated ISs; 5.4 /6.2 min	[52]
Free NM, MN and 3-MT	Plasma 100 μL	SPE on Oasis WCX μElution 96-well plates	Atlantis HILIC Silica column (2.1 × 30 mm, 3 μm)	Gradient elution: A: 100 mmol/L AF, pH 3 B: ACN	LC-MS/MS	LOD: 0.01–.03 nmol/L LOQ: 0.04–0.06 nmol/L	0.1–23.0 nmol/L	88–98	The deuterated ISs; 2.0/3.5 min	[53]
DA	Neonate plasma 200 μL	SPE-SCX and derivatization with propionic anhydride/pyridine	Waters Acquity UPLC BEH C18 column (2.1 × 100 mm, 1.7 µm)	Gradient elution: A: 0.2% FA in water B: MeOH/ACN (30:70, *v*/*v*)	LC-MS/MS	LOD: n.d. LOQ: 10 pg/mL	10–1000 pg/mL	n.d.	Dopamine-d4; 8.0/8.5 min	[54]
M, NM	Plasma 200 μL	SPE on Oasis WCX μElution 96-well plate	Waters Atlantis HILIC silica column (2.1 × 50 mm, 3.0 µm)	Gradient elution: A: 100 mmol/L AF, pH 3.0 B: ACN	UPLC-MS/MS	LOD: 0.05 nmol/L LOQ: 0.1 nmol/L	1.0–100 nmol/L	n.d.	The deuterated ISs; 15.0/20.0 min	[55]
M, NM	Plasma 0.5 mL	SPE-HyperSep and derivatization with PFHA	Hypercarb PGC column (2.1 × 50 mm, 5 µm)	Gradient elution: Three mobile phase (MP) were used. MP1: 50 mM AA and 1% FA in water; MP2: 0.1% FA in ACN; MP3: 9:9:2 (*v*/*v*/*v*) mix of ACN/isopropanol/ acetone (*v*/*v*/*v*)	LC-MS/MS	LOD: n.d. LOQ: 7.2 pg/mL for M, 18.0 pg/mL for NM	7.2–486.8 pg/mL for M 18.0–989.1 pg/mL for NM	90.5–97.5	The deuterated ISs; 5.5/10.0 min	[57]
VMA, NM, M, 3-MT	Serum 200 μL	SPE on MAX μElution 96 plate	Atlantis HILIC column (2.10 × 50 mm, 2.6 μm)	Gradient elution: A: 100 mmol/L AF, pH 2.2 B: ACN	LC-MS/MS	LOD: 0.7 nmol/L LOQ: 1.25 nmol/L	1.25–10000 nmol/L	97–99	The deuterated ISs; 3.2/10.0 min	[58]
NE, E and DA	PBMC 250 μL	SPE on Oasis HLB 96-well µElution plate	Luna PFP (2) column (2.1 × 150 mm, 3 µm)	Gradient elution: A: 0.01% FA in water B: 0.01% FA in MeOH	UPLC-MS/MS	LOD: 0.5 pg/mL for E, 2.0 pg/mL for NE and DA; LOQ: 1.0 pg/mL for E, 5.0 pg/mL for NE and DA	1.0–2500 pg/mL for E 5.0–2500 pg/mL for NE and DA	81.0–100.5	The deuterated ISs; 4.5/7.0 min	[59]
E, NE	Urine 350 μL	Alumina B 96-well SPE	Phenomenex Kinetex Piphenyl column (2.1 × 100 mm, 2.6 µm)	Gradient elution: A: 0.1% FA in water B: 0.1% FA in MeOH	LC-MS/MS	LOD: n.d. LOQ: 0.005 μmol/L	0.1–2 μmol/L	n.d.	The deuterated ISs; 4.5/6.0 min	[60]
5-HIAA	Serum 100 μL	SPE on WAX μElution plates	Atlantis HILIC Silica column (2.10 × 50 mm, 3 μm)	Gradient elution: A: 90 mmol/L AF, pH 3 B: ACN	LC-MS/MS	LOD: 0.2 nmol/L LOQ: 5 nmol/L	5–10,000 nmol/L	98	5-HIAA-D2; 3.2 /7.0 min	[61]
5-HT, 5-HIAA, DA	Urine 200 μL	SPE-Isolute C18	RP Hypersil Gold aQ column, (2.1 × 150 mm, 3 μm)	Gradient elution: A: 0.1% FA in water B: 0.1% FA in MeOH	LC-MS/MS	LOD: 8.8–18.2 nmol/L LOQ: 29.4–55.7 nmol/L	0.11–3.27 μmol/L	91–107	The deuterated ISs; 5.0/20.0 min	[62]
NE, E, DA, M, NM,	Urine 10 μL	SPE on Oasis HLB 96-well μElution plate	Luna PFP (2) column (2.1 × 150 mm, 3 µm)	Gradient elution: A: 0.01% FA water B: 0.01% FA in MeOH	LC-MS/MS	LOD: 1.0 ng/mL for NE, DA and NM; 0.25 ng/mL for E, M LOQ: 2.5 ng/mL for NE, DA and NM; 0.50 ng/mL for E, M	From 0.5 to 1250 ng/mL dependent to the analyte	74.1–96.3	The deuterated ISs; 4.0/5.5 min	[63]
NM, M, 3-MT	Plasma 900 µL	SPE on OASIS^®^WCX-96 μElution plates	Acquity UPLC HSS T3 column (2.1 × 100 mm, 1.8 µm)	Gradient elution: A: 0.2% FA in water B: 0.2% FA in ACN	UPLC-MS/MS	LOD: 0.02 nmol/L LOQ: 0.022-0.024 nmol/L	2.5-50.0 nmol/L	66–93	The deuterated ISs; 2.5/5.5 min	[64]
NE, E, DA	Urine 0.6 mL	SPE on Oasis HLB 96-well extraction plate	Restek ultra PFPP column (2.1 × 150 mm, 3 μm)	Gradient elution: A: 2% FA in water B: MeOH	LC-MS/MS	LOD: 0.1–1 ng/mL LOQ: 0.25–2.5 ng/mL	2.5-500 ng/mL for NE; 0.25–250 ng/mL for E; 2.5–1000 ng/mL for DA	n.d.	The deuterated ISs; 5.5/7.0 min	[65]
NM, M, 3-MT, NE, E, DA	Urine 100 μL for free forms 500 μL for deconjugated M, NM and 3-MT	Free BAs and their metabolites: SPE on OASIS WCX-96 μElution plates Deconjugated BAs: Hydrolyse with HCl and SPE	Acquity UPLC^®^ HSS T3 column (2.1 × 100 mm, 1.8 μm) from Waters (Eschborn, Germany)	Gradient elution: A: 0.2% FA in water B: 0.2% FA in ACN	UPLC-MS/MS	LOD: n.d. LOQ: 1.3–2.3 nmol/L	506–3264 nmol/L	60–96	The deuterated ISs; 2/n.d min	[68]
DA, E, NE, 5-HT, VMA, HVA, 3- MT, L-Tyr, L-DOPA, L-Tryp, 5-HTryp, DHBA, DOPAC, 5-HIAA	Urine 50 μL plasma 100 μL	MEPS with APS sorbent	XBridge Amide™ BEH column (3.0 × 100 mm, 3.5 µm) from Waters (Millford, Massachusetts, USA)	Gradient elution: A: 10 mM AF in water, pH 3.0 B: 10 mM AF in ACN, pH 3.0	LC-MS/MS	LOD: 2 and 10 ng/mL LOQ: 5 and 20 ng/mL dependent to the analyte	10–2000 and 20–2000 ng/mL dependent to the analyte	87.6–104.3 for plasma 84.2–98.6 for urine	DHBA; 12.0/20.0 min	[69]
5-HT, DA, NE	Urine 500 μL	MEPS C18	Zorbax 300SB C18 RP column (4.6 × 250 mm, 5 µm)	Isocratic elution: 75 mM NaH_2_PO_4_, 1.7 mM OSA, 0.01% TEA (*v*/*v*), 25 mM EDTA and 10% ACN (*v*/*v*), pH 3.5	HPLC-ED 300/150 mV in oxidation/reduction channel	LOD: 2 ng/mL for DA and NE, 20 ng/mL for 5-HT LOQ: 5 ng/mL for DA and NE, 50 ng/mL for 5-HT	5-1000 ng/mL for DA and NE, 50–1000 ng/mL for 5-HT	91.97–10.06	DHBA; 26 min	[70]
NE, E, DA	Plasma 150 μL Urine 10 μL, DPSs, DUSs (one)	MEPS C18	Hypersil Gold RP C18 column (4.6 × 150 mm, 5 µm)	Isocratic elution: MeOH: 30.0 mM CA with 0.5 mM OSA, (2.5:97.5, *v/v*), adjusted to pH 2.92 with 2 N NaOH	HPLC-CD E1/E2: −0.350/+0.400 V	LOD: 0.03 ng/mL LOQ: 0.1 ng/mL	0.1–5.0 ng/mL	86.0–95.2	Catechol; 25.0 min	[71]
5-HT	Serum 100 μL	96-well Oasis WCX μElution plates	Atlantis HILIC Silica column (2.1 × 50 mm, 3 µm)	Gradient elution: A: 90 mmol/L AF, pH 4 B: ACN	LC-MS/MS	LOD: 2.5 nmol/L LOQ: 10 nmol/L	10–10000 nmol/L	95–115	The deuterated ISs; 1.6/6.4 min	[72]
E, NE and others	Serum 0.5 mL	Deproteinization with perchloric acid; re-extraction with NaOH; derivatization with dansyl chloride	Zorbax Eclipse Plus C18 column (4.6 × 150 mm, 5 μm)	Gradient elution: A: 5 mM AF in water B: ACN	HPLC-DAD 254 nm	LOD: 90 ng/L for E, 596 ng/L for NE LOQ: 300 ng/L for E, 980 ng/L for NE	80–5 × 10 ^4^ ng/L	85.7–106.7	External method; 20 min	[75]
23 Amino acids, including L-Tryp and L-Tyr	Exhaled breath conden-sate 2 mL	Evaporation	XBridge Amide column (3.0 × 100 mm, 3.5 µm)	Gradient elution: A: 10 mM of ammonium buffer in water, pH 3 B: 10 mM ammonium buffer in ACN	LC-MS/MS	LOD: 0.25ng/mL for L-Tryp, 0.05 ng/mL for L-Tyr LOQ: 0.75 ng/mL for L-Tryp, 0.15 ng/mL for L-Tyr	0.75-400 ng/mL for L-Tryp, 0.15–400 ng/mL for L-Tyr	52.2–108.2	Leucine-D3; 12.0/18.0 min	[76]
VMA, HVA	Urine n.d.	Dilution and deproteinization with FA	Kinetex XB-C18 column (2.1 × 50 mm, 1.7 μm)	Gradient elution: A: 0.05% (*v*/*v*) FA in water B: 0.05% (*v*/*v*) FA in MeOH	LC-MS/MS	LOD: n.d. LOQ: 0.5 mg/L	0.5–100 mg/L	n.d.	d3-VMA and ring-13C6, 4-hydroxy-18O] (HVA-13C6 18O); 2.3/4.0 min	[77]

AA—ammonium acetate; ACN—acetonitrile; AF—ammonium formate; APS—aminopropyl silane sorbent; CA—citric acid; CD—coulometric detection; Cr—Creatinine; CSF—cerebrospinal fluid; DA—dopamine; DBD-PyNCS—(*R*)-(–)-4-(*N*, *N*-dimethylaminosulfonyl)-7-(3-isothiocyanatopyrrolidin-1-yl)-2,1,3-benzoxadiazole; DHBA—3,4-dihydroxybenzylamine; DLLME—dispersive liquid-liquid microextraction; DOPAC—3,4-dihydroxyphenylacetic acid; DPE—1,2-diphenylethyleendiamine; DPSs—dried plasma spots; DUSs—dried urine spots; E—epinephrine; ED—electrochemical detection; FA—formic acid; HAc—acetic acid; HFBA—4-(3′,3′,4′,4′,5′,5′,6′,6′,7′,7′,8′,8′,9′,9′,10′,10′,10′-heptadecafluorodecyl)benzylamine; HLB—hydrophilic-lipophilic balance; 5-HIAA—5-hydroxyindole-3-acetic acid; HILIC—zwitter-ionic phase; HMPG sulphate—4-hydroxy-3-methoxyphenylglycol sulphate; HPLC—high performance liquid chromatography; 5-HT—5-hydroxytryptamine (serotonin); 5-HTryp—5-hydroxytryptophane; HVA—homovanillic acid; IL—anionic liquid; IS—Internal Standard; KYN—kynurenine; LC—liquid chromatography; LC-MS/MS—liquid chromatography with tandem mass spectrometry; L-DOPA—3,4-dihydroxyphenylalanine; LLE—liquid-liquid extraction; LOD—limit od detection; LOQ—limit of quantification; L-Tyr—L-tyrosine; L-Tryp—L-tryptophan; M—metanephrine; MHPG—3-metoxy-4-hydroxyphenolglycol; MD—microdialysis device; MeOH—methanol; MEPS—microextraction in packed syringe; 3-MT—3-methoxytyramine; NaAc—sodium acetate; NE—norepinephrine; n.d.—no data; NM—normetanephrine; OSA—sodium octyl sulphate; PBMC—peripheral blood mononuclear cells; PFHA—perfluoroheptanoic acid; PFOEI—2-(perfluorooctyl)ethyl isocyanate; PITC—phenylisothiocyanate SCX—strong cation exchange; SPE—solid phase extraction; TCA—trichloroacetic acid; TEA—trimethylamine; TFA—trifluoroacetic acid; THF—tetrahydrofuran; UPLC—ultra performance liquid chromatography; UV—ultraviolet detection; WCX—weak cation exchange; VMA—vanillylmandelic acid.

**Table 3:** GC methods for the separation of selected BAs in human specimens published since 2010.

Analytes	Human Sample Volume	Sample Preparation	GC Conditions		Validation Data			Internal Standard (IS); Analysis Time	Ref.
			Solid Phase	Detector	LOD/LOQ	Linearity Range	Absolute Recovery (%)		
L-Tryp, 5-HT and others	Plasma 100 μL	Deproteinization with TCA, centrifugation and SPE-Empore TM C18 cartridge, Derivatization with PFPOH:PFPA (2:1, *v*/*v*)	HP-1MS^®^ capillary column (30.0 m × 0.25 mm, 0.25 μm film thick-ness)	GC/MS	LOD: n.d LOQ: 5 pmol/L for L-Tryp 2.5 pmol/L for 5-HT	5–60 pmol/L for L-Tryp 2.5–40 pmol/L for 5-HT	83.6–87.2	The deuterated ISs; 10 min	[33]
HVA, VMA	Urine 1 mL	LLE with ethyl acetate and derivatization with BSTFA	HP-5MS fused-silica capillary column (30 m × 0.25 mm, 0.25 μm film thickness)	GC-MS	LOD: n.d. LOQ: 0.9 μmol/L	0.9–193 and 0.9–221 μmol/L for HVA and VMA	92–96	3-Phenyl butyric acid; 16 min	[45]
56 amino acids and their conjugates, 84 organic acids, 9 BAs amines, 4 other analytes	Urine 25 µL	LLME with the THP and re-extraction to acidic isooctane; Derivatization with HFBCF	ZB-XLB silica capillary column (30 m x 0.25 mm, 0.25 µm film thickness)	GC-MS	for BAs LOD: 0.013–0.05 µmol/L LOQ: 2–4 µmol/L for BAs	For BAs 2–400 µmol/L dependent to the analyte	99–116	External method; 38.0 min	[47]
DA, HVA, L-DOPA, NM, E, NE, DOPAC, VMA, 5-HT, 5-HIAA	Urine 2 mL	Hydrolysis with HCl, SPE-MCX and derivatization with HMDS/MBHFBA	DB-5MS capillary column (15 m × 0.25 mm, 0.25 μm film thickness)	GC/MS	LOD: 0.12– 6.24 ng/mL LOQ: 0.17–17.84 ng/mL	From 1 to 2500 ng/mL dependent to the analyte	n.d.	The deuterated ISs; 13.5 min	[66]
20 Analytes	Urine 2 mL	Hydrolysis with HCl, next, SPE-MCX and derivatization with HMDS/MBHFBA	DB-5MS capillary column (20 m × 0.25 mm, 0.25 μm film thick-ness)	GC-MS/MS	LOD:0.47–1.72 ng/mL LOQ: 1.0–25.0 ng/mL	From 1 to 1000 ng/mL dependent to the analyte	n.d.	The deuterated ISs; 15 min	[67]
HVA, VMA, 5-HIAA	Urine 50 μL	Derivatization with ethyl chloroformate/ethanol and SPME with PA fibre in immersion mode	Thermo TR-5MS (30 m × 0.25 mm, 0.25 µm film thickness)	GC–QqQ-MS	LOD: 1.3, 0.046 and 24.3 ug/L for HVA, VMA and 5-HIAA LOQ: 2.7, 0.063 and 49.6 μg/L for HVA, VMA and 5-HIAA	0.5–100 μg/L	n.d.	The deuterated ISs; 25 min	[73]
DA, 5-HT and NE	Urine 600 µL	Derivatization with propyl chloroformate, Next, SPME with PA fibre in immersion mode	Thermo TR-5MS (30 m × 0.25 mm, 0.25 μm film thickness)	GC-QqQ-MS	LOD: 0.38–13.5 µg/L LOQ: 0.81, 0.74 and 21.3 µg/L for DA, 5-HT and NE	10–1000 µg/L for DA and 5-HT 30–1000 µg/L for NE	n.d.	The deuterated ISs; 20 min	[74]

BSTFA—N,O-bis(trimethylsilyl)trifluoroacetamide; DA—dopamine; DOPAC—3,4-dihydroxyphenylacetic acid; E—epinephrine; GC-MS—gas chromatography with mass spectrometry; GC-MS/MS—gas chromatography with tandem mass spectrometry; GC-QqQ-MS—gas chromatography-triple quadrupole mass spectrometry; HFBCF—heptafluorobutyl chloroformate; 5-HIAA—5-hydroxyindole-3-acetic acid; HMDS—hexamethyldisilazane; 5-HT—5-hydroxytryptamine (serotonin); HVA—homovanillic acid; L-DOPA—3,4-dihydroxyphenylalanine; LLE—liquid-liquid extraction; L-Tyr—L-tyrosine; L-Tryp—L- tryptophan; M—metanephrine; MBHFBA -N-methyl-bis-heptafluorobutyramide; MCX—mixed mode cation exchange; NE—norepinephrine; n.d.—no data; NM—normetanephrine; LLE—liquid–liquid extraction; LLME—liquid-liquid microextraction; LOD—limit od detection; LOQ—limit of quantification; SPE—solid phase extraction; SPME—solid phase microextraction; PA—polyacrylate; PFPA—pentafluoropropionic anhydride; PFPOH—2,2,3,3,3-pentafluoro-1-propanol; TCA—trichloroacetic acid, THP—tris(3-hydroxypropyl)phosphine; VMA—vanillylmandelic ac.

**Table 4:** Information on parameter methodologies based on the electromigration techniques developed for BA determinations in human samples since 2010.

Separation Technique										
Analytes	Matrix Sample/Volume	Sample Preparation	CE Parameters			Validation Data			Analysis Time (min)/IS	Ref.
			Technique (and Preconcentration) Mode	BGE	Detection Mode	LOD/LOQ	Linearity Range	Absolute Recovery (%)		
DA, 3-MT, 5-HT	Artificial urine/n.d.	Molecularly imprinted polymer (MIP)-SPE	Field-amplified sample injection (FASI)-CE	80 mM H_3_PO_4_–LiOH (pH 4) Sample buffer: 100 μM phosphate in methanol–water 90/10 (*v*/*v*)	UV 214 nm	LOQ: 46 nM for DA,11 nM for 3-MT, 6 nM for 5-HT LOQ: 50 nM	50–300 nM	40–100	10/3,4-DHBA	[89]
E, NE, DA, NM	Urine/ 4 mL	SPE alumina	HC FASS–CZE, sweeping, electro-stacking	50 mM acetate buffer pH 4.0, 0.5 mM ionic liquid C16MimCl	UV 220 nm	LOD: 0.3–1.3 ng/mL LOQ: -	-	85–90	10/n.d.	[90]
HA, L-Try, TA, 5-HT, DA, NE, E	Urine/n.d.	Centrifugation	CZE	150.0 mM phosphate (Na_2_HPO_4_–NaH_2_PO_4_) and 1.0 mM borax, pH 6.1	DAD 200 nm	LOD: 0.2–1.2 μM LOQ: 0.7–4 μM	0.7–50 μM	-	15/n.d.	[91]
DA, E	Urine/0.5 mL	Centrifugation, dilution and filtration	Laboratory- built CE–CL	25 mM borate solution (pH 9.8) containing 0.6 mM CdTe quantum dot (QD)s and 0.3 mM luminol	CL	LOD: 23 nM for DA, 9.3 μM for E LOQ: 0.08 µM for DA, 0.04 μM for E	0.08–5 μM for DA 0.04–5 μM for E	97–104	10/n.d.	[92]
DA, TA, T, 5-HT, E	Urine/ 1 mL	Filtered and LLE	FASI-CE	20 mM of 2-(morpholino)-ethanesulfonic acid and 30 mM phosphate buffer, 0.05% hydroxypropylcellulose and 10% *v*/*v* methanol, pH 6.5	UV 210 nm	LOD: 0.01–0.15 μM LOQ: n.d.	0–10 μM	89–108	25/PPA	[93]
E	Urine 0.2 mL	Deproteinization and filtration	CE-CL	2.5 mM phosphate buffer (pH 6.0) and 100 μM luminol	CL	LOD: 0.82 ng/mL LOQ: 2.0 ng/mL	2.0–400 ng/mL	86.5–112	7/n.d.	[94]
VMA, HVA, DOPAC	Urine/ 1 mL	LLE diethyl ether 1 mL	MEKC UV/VIS	10 mM sodium tetraborate decahydrate. 30 mM SDS. 15% (*v*/*v*) MeOH.25 mM α-CD. adjusted to pH 9.36 with 1 N NaOH	DAD 200 nm	LOD: 0.1–0.25 μg/mL LOQ: 0.5 μg/mL	0.5–50 μg/mL for VMA and DOPAC, 0.5–100 μg/mL for HVA	>96.1	16/3,4-DHBA	[95]
DA, E, NE, L-Tryp, L-Tyr, 5-HT, L-DOPA	Urine/ 1 mL	DLLME	MEKC	10 mM sodium tetraborate decahydrate, 30 mM SDS, 15% (*v*/*v*) MeOH,25 mM α-CD, adjusted to pH 9.36 with 1 N NaOH	DAD 200 nm	LOD: 0.15μg/mL LOQ: 0.5 μg/mL for all analytes	0.5–300 μg/mL	92.5–108.3	10–15/4-MT	[96]
DA, A, NA, L-Tryp, L-Tyr	Artificial urine/ 1 mL	SPME C18 with ionic liquid	MEKC	10 mM sodium tetraborate decahydrate. 30 mM SDS. 15% (*v*/*v*) MeOH.25 mM α-CD. adjusted to pH 9.36 with 1 N NaOH	DAD 200 nm	- -	-	-	8–10/n.d.	[97]
L-Tyr, 5-HT, 3-MT, L-Tryp, 5-HIAA, VMA, NM, DA, L-DOPA	Urine/ 0.5 mL	Deproteinization and centrifugation	FASS CE -stacking	500 mM Tris–borate (TB) and 10% (*v*/*v*) glycerol pH 9; The ends of the capillary were immersed in the cathodic and anodic vials that both contain 500 mM TB (pH 9.0) and 0–1% PEO	UV 220 nm	LOD: 17.3-313.4 nM LOQ: 1 µM for L-Tyr, 5-HT, 3-MT 10 µM for L-Tryp 5-HIAA, VMA	1–10 μM for L-Tyr, 5-HT, 3_MT; 10–75 μM for L-Tryp, 5-HIAA, VMA	93–109	35/n.d.	[98]
5-HT, DA, NE	Urine/n.d.	SPE alumina B column	FASS-RFS-MCE	10 mM borate solution (pH 9.4)	LIF	LOD: 1.69– 2.73 nM LOQ: 2.0–4.05 nM	2–99 nM for 5-HT; 4–120 nM for DA; 4–110 nM for NE	101.8– 106.4	3/n.d.	[99]
E, NE, DA	Urine/ 5 mL	Centrifugation	laboratory-built CE–CL	1.0 mM luminol in 10 mM sodium borate (pH 9.3)	CL	LOD: 79 nM for E, 100 nM for NE, 69 nM for DA LOQ: -	0.079–3 μM for E; 0.1–5 μM for NE; 0.1–5 μM for DA	96–110 94.7–103.5 92.4–99.4	8/n.d.	[100]
L-Tyr, L-Tryp, NE, DA, 5-HT	breast cancer cells /n.d.	Centrifugation and filtration	CE-LEDIF	0.5 M Tris –borate (pH 10.2) and 30 mM SDS	LIF	LOD: 2.06–19.17 nM LOQ: 15 nM	15–1000 nM	-	16/n.d.	[101]
L-Tryp, 5-HT, L-Tyr, DA, E, NE	Urine/n.d.	SPE-alumina B column	CE-dynamic pH junction	150 mM boric acid. 1 mM ascorbic acid. pH 10.33; sample matrix: 150 mM acetic acid–sodium acetate. pH 3.60.	AmpD	LOD: 6.15–68.3 nM LOQ: 0.04–0.23 μM	0.15–148 μM	83.6–138.3	20/n.d.	[102]
DA, E, 5-HT	CSF/ n.d.	Deproteinization and centrifugation	PDMS -MCE	20 mM PBS	AmpD	LOD: 2–6 μM LOQ: 10 μM	10–600 μM for DA; 10–500 μM for E; 10–600 μM for 5-HT	91.7–106.5	1.5/n.d.	[103]
DA	Urine/ 3 mL	SPE Oasis HLB	CE	0.2 mM PBS (pH 11.5) containing 0.1 mM luminol	CL	LOD: 6 ng/mL LOQ: 10 ng/mL	0.01–50 µg/mL	71.2–83.0	12/n.d.	[104]
VMA, HVA, L-Tryp, NM, E, 5-HT	Urine/ n.d.	Filtration and dilution in BGE	CZE stacking	50 mM H_3_PO_4_-Tris. pH 4	ED	LOD: 0.4–17 μM LOQ: 1.25–5 μM	1.25–50 μM for VMA, HVA and L-Tryp; 5–200 μM for NM and E; 2.5–100 μM for 5-HT	-	13/n.d.	[105]

ACN—acetonitrile, AmpD—amperometric detection, BGE—back ground electrolyte, CD—cyclodexrin, CL—chemiluminescence, CE—capillary electrophoresis, CE-LEDIF—light-emitting diode-induced fluorescence; CSF—cerebrospinal fluid, CZE—capillary zone electrophoresis, DA—dopamine, DAD—diode array detection, 3,4-DHBA—3,4-dihydoxybenzoic acid, E—epinephrine, ED—electrochemical detection, FASI—field-amplified sample injection, FASS—field amplified sample stacking, FASS-RFS-MCE—field-amplified stacking (FASS) and reversed-field stacking (RFS) microchip electrophoresis (MCE); HC FASS–CZE—head-column field amplified sample stacking capillary zone electrophoresis, 5-HIAA—5-hydroxyindole-3-acetic acid, 5-HT—serotonin, IS—internal standard, L-DOPA—levodopa, MEKC—micellar electrokinetic capillary chromatography, 3-MT—3-methoxytyramine, 4-MT—4-methoxytyramine NE—norepinephrine, NM—normetanephrine, PBS—phosphate buffer saline, PDMS—Native Poly(dimethyl-siloxane), PPA—phenylpropanolamine, SDS—sodium dodecyl sulphate, T—tryptamine, TA—tyramine, L-Tryp—L-tryptophan, L-Tyr—L-tyrosine, UV—ultra-violet detection, VMA—vanillylmandelic acid.

## Abbreviations

2-MHA	2-methylhippuric acid
2-PB	2-picoline borane
5-HIAA	5-hydroxyindoleacetic acid
5-HT	5-hydroxytryptamine; serotonine
5-HTrp	5-hydroxytryptophan
AA	ascorbic acid
ACN	acetonitrile
AD	Alzheimer’s Disease
ADHD	Attention-Deficit/Hyperactivity Disorder
AmpD	amperometric detection
APCI	atmospheric pressure chemical ionization
APHD	Huntington’s Disease
APS	amino-propyl silane
AuNPs	gold nanoparticles
BAs	biogenic amines
BDS	base-deactivated silica
CAR/PDMS	carboxen/polydimethylsiloxane
CATs	catecholamines
CE	capillary electrophoresis
CE-AmpD	capillary electrophoresis with amperometric detection
CILE	carbon ionic liquid electrode
CKD	chronic kidney disease
CL	chemiluminescence detection
CNS	central nervous system
COMT	catechol-O-methyltransferase
Cr	creatinine
CSF	cerebrospinal fluid
CSC	carcinoma stem cell sample
CTAB	cetyltrimethylammonium bromide
CZE	capillary zone electrophoresis
DA	dopamine
DAD	diode array detection
DBD-PyNCS	4-(*N*, *N*-dimethylaminosulfonyl)-7-(3-isothiocyanatopyrrolidin-1-yl)-2,1,3-benzoxadiazole
DHEAS	dehydroepiandrosterone
DHPG	3,4-dihydroxyphenylglycol
DLLME	dispersive liquid-liquid extraction
DOPAC	3,4-dihydroxyphenylacetic acid
DPE	diphenylethylendiamine
DPSs	dried plasma spots
DUSs	dried urine spots
DVB/CAR/PDMS	divinylbenzene/carboxen/polydimethylsiloxane
E	epinephrine
ED	electrochemical detection
eGFR	glomerular filtration rate
ELISA	enzyme-linked immunosorbent assay
EOF	electroosmotic flow
ESI	electrospray ionization
FA	formic acid
FASI	field amplified sample injection
FASS	field-amplified sample stacking
GABA	γ-aminobutyric acid
GC	gas chromatography
GCE	glass carbon electrode
GC-MS	gas chromatography coupled with mass spectrometry
GC-MS/MS	gas chromatography coupled with tandem mass spectrometry
GC–QqQ-MS	gas chromatography-triple quadrupole mass spectrometry
GITS	non-neural neoplasm gastrointestinal tumor
Glu	glutamate
GR	graphene
HA	hippuric acid
HC	head-column
HE	Hashimoto Encephalopathy
HFBA	4-(heptadecafluorodecyl)benzylamine
HFBCF	1,1,1,2,2,3,3-heptafluorobutyl chloroformate reagent
HILIC	hydrophilic interaction liquid chromatography
HLB	hydrophilic-lipophilic balanced copolymer sorbent
HMDS	hexamethyldisilazane
HMPG sulfate	4-hydroxy-3-methoxyphenylglycol sulfate
HPLC-(ESI)-MS	high-performance liquid chromatography/electrospray ionization mass spectrometry
HPLC-FD	high performance liquid chromatography with fluorescence detection
HPLC-UV	high performance liquid chromatography with ultraviolet detection
HVA	homovanillic acid
IAA	indole-3-acetic acid
ITP	isotachophoresis
LC	liquid chromatography
LC-ESI-MS/MS	liquid chromatography/electrospray ionization tandem mass spectrometry
LC-FL	liquid chromatography with fluorescence detection
LC-MS/MS	liquid chromatography coupled with tandem mass spectrometry
L-DOPA	L-dihydroxyphenylalanine
LEDIF	light emitting diode-induced fluorescence
LIF	laser-induced fluorescence
L-KYN	L-kynurenine
LLE	liquid-liquid extraction
LLME	liquid-liquid microextraction
LLOQ	lower limit of quantification
LOD	limit of detection
LOQ	limit of quantification
L-Tryp	L-tryptophan
L-Tyr	L-Tyrosine
M	metanephrine
MAO	monoamine oxidase
MAOA	monoamineoxidase A
MBHFBA	N-methyl-bis-heptafluorobutyramide
MCE	microchip electrophoresis
MCX	mixed mode cation exchange
MEKC	micellar electrokinetic chromatography
MEPS	microextraction by packed sorbent
MHPG	3-methoxy-4-hydroxyphenylglycol
MIPs	molecularly imprinted polymers
MNs	metanephrines
MNTI	melanotic neuroectodermal tumor of infancy
MRM	multiple reaction monitoring
NA	niacinamide
NBL	neuroblastoma
NE	norepinephrine
NENs	neuroendocrine neoplasms
NETs	neuroendocrine tumors
NM	normetanephrine
OCT	octopamine hydrochloride
PA	polyacrylate
PBA	diphenylborate
PBMC	peripheral blood mononu-clear cells
PCR	polymerase chain reaction
PD	Parkinson’s Disease
PDDA	poly(diallyl dimethylammonium) chloride
PDMS	polydimethylsiloxane
PDMS/DVB	polydimethylsiloxane/divinylbenzene
PEA	phenylethylamine
PEO	polyethylene oxide
PFHA	perfluoroheptanoic acid
PFOEI	2-(perfluorooctyl)ethyl isocyanate
PFUA	perfluoroundecan-1-al
PGC	porous graphitic carbon
PHE	pheochromocytoma
PITC	phenylisothiocyanate
PTSD	post-traumatic stress disorder
RFS	reversed-field stacking
RP18	reversed phase C18 column
SCX	strong cation exchange
SDS	sodium dodecyl sulphate
SPE	solid phase extraction
SPME	solid phase microextraction
SIM	selective ion monitoring
SRM	selective reaction monitoring
TB	tris-borate buffer
TCA	trichloroacetic acid
THF	tetrahydrofuran
THP	tris(3-hydroxypropyl)phosphine
UA	uric acid
UPLC	ultrafast performance liquid chromatography
UV	ultraviolet
UV/Vis	combined ultraviolet and visible radiation detectors,
VMA	vanillymandelic acid
WCX	weak cation exchange

## References

[1] Daws L.C., Owens W.A., Toney G.M. (2016). Using High-Speed Chronoamperometry to Measure Biogenic Amine Release and Uptake In Vivo. Neurotransmitter Transporters.

[2] Lv C., Li Q., Liu X., He B., Sui Z., Xu H., Yin Y., Liu R., Bi K. (2015). Determination of catecholamines and their metabolites in rat urine by ultra-performance liquid chromatography-tandem mass spectrometry for the study of identifying potential markers for Alzheimer’s disease. J. Mass Spectrom..

[3] Vermeiren Y., Le Bastard N., Van Hemelrijck A., Drinkenburg W.H., Engelborghs S., De Deyn P.P. (2013). Behavioral correlates of cerebrospinal fluid amino acid and biogenic amine neurotransmitter alterations in dementia. Alzheimer’s Dement..

[4] Brichta L., Greengard P., Flajolet M. (2013). Advances in the pharmacological treatment of Parkinson’s disease: Targeting neurotransmitter systems. Trends Neurosci..

[5] Miękus N., Bączek T. (2016). Non-invasive screening for neuroendocrine tumors—Biogenic amines as neoplasm biomarkers and the potential improvement of “gold standards”. J. Pharm. Biomed. Anal..

[6] Palego L., Betti L., Rossi A., Giannaccini G. (2016). Tryptophan Biochemistry: Structural, Nutritional, Metabolic and Medical Aspects in Humans. J. Amino Acids.

[7] Marcos J., Renau N., Valverde O., Aznar-Laín G., Gracia-Rubio I., Gonzalez-Sepulveda M., Pérez-Jurado L.A., Ventura R., Segura J., Pozo O.J. (2016). Targeting tryptophan and tyrosine metabolism by liquid chromatography tandem mass spectrometry. J. Chromatogr. A.

[8] Jenkins T., Nguyen J., Polglaze K., Bertrand P. (2016). Influence of Tryptophan and Serotonin on Mood and Cognition with a Possible Role of the Gut-Brain Axis. Nutrients.

[9] Sánchez-López E., Marcos A., Ambrosio E., Marina M.L., Crego A.L. (2016). Enantioseparation of the constituents involved in the phenylalanine-tyrosine metabolic pathway by capillary electrophoresis tandem mass spectrometry. J. Chromatogr. A.

[10] Eisenhofer G. (2004). Catecholamine Metabolism: A Contemporary View with Implications for Physiology and Medicine. Pharmacol. Rev..

[11] Andreou D., Söderman E., Axelsson T., Sedvall G.C., Terenius L., Agartz I., Jönsson E.G. (2014). Polymorphisms in genes implicated in dopamine, serotonin and noradrenalin metabolism suggest association with cerebrospinal fluid monoamine metabolite concentrations in psychosis. Behav. Brain Funct..

[12] Yu J., Kong L., Zhang A., Han Y., Liu Z., Sun H., Liu L., Wang X. (2017). High-Throughput Metabolomics for Discovering Potential Metabolite Biomarkers and Metabolic Mechanism from the APPswe/PS1dE9 Transgenic Model of Alzheimer’s Disease. J. Proteome Res..

[13] de Jong W.H.A., de Vries E.G., Kema I.P. (2011). Current status and future developments of LC-MS/MS in clinical chemistry for quantification of biogenic amines. Clin. Biochem..

[14] Bicker J., Fortuna A., Alves G., Falcão A. (2013). Liquid chromatographic methods for the quantification of catecholamines and their metabolites in several biological samples—A review. Anal. Chim. Acta.

[15] Eisenhofer G., Peitzsch M., McWhinney B.C. (2016). Impact of LC-MS/MS on the laboratory diagnosis of catecholamine-producing tumors. TrAC Trends Anal. Chem..

[16] Parent A.J., Beaudet N., Daigle K., Sabbagh R., Sansoucy Y., Marchand S., Sarret P., Goffaux P. (2015). Relationship Between Blood- and Cerebrospinal Fluid–Bound Neurotransmitter Concentrations and Conditioned Pain Modulation in Pain-Free and Chronic Pain Subjects. J. Pain.

[17] Taj A., Jamil N. (2018). Cerebrospinal Fluid Concentrations of Biogenic Amines: Potential Biomarkers for Diagnosis of Bacterial and Viral Meningitis. Pathogens.

[18] Grace A.A., Gerfen C.R., Aston-Jones G. (1997). Catecholamines in the Central Nervous System. Adv. Pharmacol..

[19] Plonka J. (2015). Methods of biological fluids sample preparation-biogenic amines, methylxanthines, water-soluble vitamins. Biomed. Chromatogr..

[20] Chan E.C.Y., Wee P.Y., Ho P.C. (2000). Evaluation of degradation of urinary catecholamines and metanephrines and deconjugation of their sulfoconjugates using stability-indicating reversed-phase ion-pair HPLC with electrochemical detection. J. Pharm. Biomed. Anal..

[21] Cudjoe E., Pawliszyn J. (2014). Optimization of solid phase microextraction coatings for liquid chromatography mass spectrometry determination of neurotransmitters. J. Chromatogr. A.

[22] Remane D., Grunwald S., Hoeke H., Mueller A., Roeder S., von Bergen M., Wissenbach D.K. (2015). Validation of a multi-analyte HPLC-DAD method for determination of uric acid, creatinine, homovanillic acid, niacinamide, hippuric acid, indole-3-acetic acid and 2-methylhippuric acid in human urine. J. Chromatogr. B.

[23] Clark Z.D., Cutler J.M., Frank E.L. (2017). Practical LC-MS/MS Method for 5-Hydroxyindoleacetic Acid in Urine. J. Appl. Lab. Med..

[24] Zhao J., Chen H., Ni P., Xu B., Luo X., Zhan Y., Gao P., Zhu D. (2011). Simultaneous determination of urinary tryptophan, tryptophan-related metabolites and creatinine by high performance liquid chromatography with ultraviolet and fluorimetric detection. J. Chromatogr. B.

[25] Konieczna L., Roszkowska A., Stachowicz-Stencel T., Synakiewicz A., Bączek T. (2018). Bioanalysis of a panel of neurotransmitters and their metabolites in plasma samples obtained from pediatric patients with neuroblastoma and Wilms’ tumor. J. Chromatogr. B.

[26] Miller A.G., Brown H., Degg T., Allen K., Keevil B.G. (2010). Measurement of plasma 5-hydroxyindole acetic acid by liquid chromatography tandem mass spectrometry—Comparison with HPLC methodology. J. Chromatogr. B.

[27] Cai H.-L., Zhu R.-H., Li H.-D. (2010). Determination of dansylated monoamine and amino acid neurotransmitters and their metabolites in human plasma by liquid chromatography–electrospray ionization tandem mass spectrometry. Anal. Biochem..

[28] Ji C., Walton J., Su Y., Tella M. (2010). Simultaneous determination of plasma epinephrine and norepinephrine using an integrated strategy of a fully automated protein precipitation technique, reductive ethylation labeling and UPLC–MS/MS. Anal. Chim. Acta.

[29] Fang L., Lv Y., Sheng X., Yao S. (2012). Sensitive, Rapid and Easy Analysis of Three Catecholamine Metabolites in Human Urine and Serum by Liquid Chromatography Tandem Mass Spectrometry. J. Chromatogr. Sci..

[30] Boulet L., Faure P., Flore P., Montérémal J., Ducros V. (2017). Simultaneous determination of tryptophan and 8 metabolites in human plasma by liquid chromatography/tandem mass spectrometry. J. Chromatogr. B.

[31] Zhu W., Stevens A.P., Dettmer K., Gottfried E., Hoves S., Kreutz M., Holler E., Canelas A.B., Kema I., Oefner P.J. (2011). Quantitative profiling of tryptophan metabolites in serum, urine and cell culture supernatants by liquid chromatography–tandem mass spectrometry. Anal. Bioanal. Chem..

[32] Zhao J. (2015). Simultaneous determination of plasma creatinine, uric acid, kynurenine and tryptophan by high-performance liquid chromatography: Method validation and in application to the assessment of renal function. Biomed. Chromatogr..

[33] Sano M., Ferchaud-Roucher V., Nael C., Aguesse A., Poupeau G., Castellano B., Darmaun D. (2014). Simultaneous detection of stable isotope-labeled and unlabeled l-tryptophan and of its main metabolites, l-kynurenine, serotonin and quinolinic acid, by gas chromatography/negative ion chemical ionization mass spectrometry. J. Mass Spectrom..

[34] Shen Y., Lu J., Tang Q., Guan Q., Sun Z., Li H., Cheng L. (2015). Rapid, easy analysis of urinary vanillylmandelic acid for diagnostic testing of pheochromocytoma by liquid chromatography tandem mass spectrometry. J. Chromatogr. B.

[35] Zheng J., Mandal R., Wishart D.S. (2018). A sensitive, high-throughput LC-MS/MS method for measuring catecholamines in low volume serum. Anal. Chim. Acta.

[36] Baranyi A., Meinitzer A., Rothenhäusler H.-B., Amouzadeh-Ghadikolai O., Lewinski D.V., Breitenecker R.J., Herrmann M. (2018). Metabolomics approach in the investigation of depression biomarkers in pharmacologically induced immune-related depression. PLoS ONE.

[37] Sakaguchi Y., Yoshida H., Hayama T., Itoyama M., Todoroki K., Yamaguchi M., Nohta H. (2011). Selective liquid-chromatographic determination of native fluorescent biogenic amines in human urine based on fluorous derivatization. J. Chromatogr. A.

[38] Sakaguchi Y., Ikenaga J., Yoshida H., Hayama T., Itoyama M., Todoroki K., Imakyure O., Yamaguchi M., Nohta H. (2015). Selective and sensitive liquid chromatographic determination method of 5-hydroxyindoles with fluorous and fluorogenic derivatization. J. Pharm. Biomed. Anal..

[39] Ohashi H., Iizuka H., Yoshihara S., Otani H., Kume M., Sadamoto K., Ichiba H., Fukushima T. (2013). Determination of L-tryptophan and L-kynurenine in Human Serum by using LC-MS after Derivatization with (R)-DBD-PyNCS. Int. J. Tryptophan Res..

[40] Sa M., Ying L., Tang A.-G., Xiao L.-D., Ren Y.-P. (2012). Simultaneous determination of tyrosine, tryptophan and 5-hydroxytryptamine in serum of MDD patients by high performance liquid chromatography with fluorescence detection. Clin. Chim. Acta.

[41] Ellis A.G., Zeglinski P.T., Coleman K.E., Whiting M.J. (2017). Dilute, derivatise and shoot: Measurement of urinary free metanephrines and catecholamines as ethyl derivatives by LC-MSMS. Clin. Mass Spectrom..

[42] Tang Y.-B., Sun F., Teng L., Li W.-B., An S.-M., Zhang C., Yang X.-J., Lv H.-Y., Ding X.-P., Zhu L. (2014). Simultaneous determination of the repertoire of classical neurotransmitters released from embryonal carcinoma stem cells using online microdialysis coupled with hydrophilic interaction chromatography–tandem mass spectrometry. Anal. Chim. Acta.

[43] Sadilkova K., Dugaw K., Benjamin D., Jack R.M. (2013). Analysis of vanillylmandelic acid and homovanillic acid by UPLC–MS/MS in serum for diagnostic testing for neuroblastoma. Clin. Chim. Acta.

[44] Diniz M.E.R., Vilhena L.S., Paulo B.P., Barbosa T.C.C., Mateo E.C. (2015). Simultaneous Determination of Catecholamines and Metanephrines in Urine by Liquid Chromatography Electrospray Ionization Tandem Mass Spectrometry: Successful Clinical Application. J. Braz. Chem. Soc..

[45] Tran M.T.C., Baglin J., Tran T.T.T., Hoang K.T., Phung L.T., Read A., Greaves R.F. (2014). Development of a new biochemical test to diagnose and monitor neuroblastoma in Vietnam: Homovanillic and vanillylmandelic acid by gas chromatography–mass spectrometry. Clin. Biochem..

[46] Gosetti F., Mazzucco E., Gennaro M.C., Marengo E. (2013). Simultaneous determination of sixteen underivatized biogenic amines in human urine by HPLC-MS/MS. Anal. Bioanal. Chem..

[47] Hušek P., Švagera Z., Hanzlíková D., Řimnáčová L., Zahradníčková H., Opekarová I., Šimek P. (2016). Profiling of urinary amino-carboxylic metabolites by in-situ heptafluorobutyl chloroformate mediated sample preparation and gas chromatography–mass spectrometry. J. Chromatogr. A.

[48] Konieczna L., Roszkowska A., Niedźwiecki M., Bączek T. (2016). Hydrophilic interaction chromatography combined with dispersive liquid–liquid microextraction as a preconcentration tool for the simultaneous determination of the panel of underivatized neurotransmitters in human urine samples. J. Chromatogr. A.

[49] Hayama T., Yabuuchi Y., Iwamatsu T., Tamashima E., Kawami Y., Itoyama M., Yoshida H., Yamaguchi M., Nohta H. (2013). Concerted derivatization and concentration method with dispersive liquid–liquid microextraction for liquid chromatographic analysis of 5-hydroxyindoles in human serum. Talanta.

[50] Woo H.I., Yang J.S., Oh H.J., Cho Y.Y., Kim J.H., Park H.-D., Lee S.-Y. (2016). A simple and rapid analytical method based on solid-phase extraction and liquid chromatography–tandem mass spectrometry for the simultaneous determination of free catecholamines and metanephrines in urine and its application to routine clinical analysis. Clin. Biochem..

[51] Johnsen E., Leknes S., Wilson S.R., Lundanes E. (2015). Liquid chromatography-mass spectrometry platform for both small neurotransmitters and neuropeptides in blood, with automatic and robust solid phase extraction. Sci. Rep..

[52] Zhang G., Zhang Y., Ji C., McDonald T., Walton J., Groeber E.A., Steenwyk R.C., Lin Z. (2012). Ultra sensitive measurement of endogenous epinephrine and norepinephrine in human plasma by semi-automated SPE-LC–MS/MS. J. Chromatogr. B.

[53] Peaston R.T., Graham K.S., Chambers E., van der Molen J.C., Ball S. (2010). Performance of plasma free metanephrines measured by liquid chromatography–tandem mass spectrometry in the diagnosis of pheochromocytoma. Clin. Chim. Acta.

[54] Zhang D., Wu L., Chow D.S.L., Tam V.H., Rios D.R. (2016). Quantitative determination of dopamine in human plasma by a highly sensitive LC-MS/MS assay: Application in preterm neonates. J. Pharm. Biomed. Anal..

[55] Petteys B.J., Graham K.S., Parnás M.L., Holt C., Frank E.L. (2012). Performance characteristics of an LC–MS/MS method for the determination of plasma metanephrines. Clin. Chim. Acta.

[56] Tamashima E., Hayama T., Yoshida H., Imakyure O., Yamaguchi M., Nohta H. (2015). Direct tandem mass spectrometric analysis of amino acids in plasma using fluorous derivatization and monolithic solid-phase purification. J. Pharm. Biomed. Anal..

[57] He X., Gabler J., Yuan C., Wang S., Shi Y., Kozak M. (2011). Quantitative measurement of plasma free metanephrines by ion-pairing solid phase extraction and liquid chromatography–tandem mass spectrometry with porous graphitic carbon column. J. Chromatogr. B.

[58] Tohmola N., Itkonen O., Turpeinen U., Joenväärä S., Renkonen R., Hämäläinen E. (2015). Preanalytical validation and reference values for a mass spectrometric assay of serum vanillylmandelic acid for screening of catecholamine secreting neuroendocrine tumors. Clin. Chim. Acta.

[59] Li X.S., Li S., Kellermann G. (2016). An integrated liquid chromatography-tandem mass spectrometry approach for the ultra-sensitive determination of catecholamines in human peripheral blood mononuclear cells to assess neural-immune communication. J. Chromatogr. A.

[60] Bergmann M.L., Sadjadi S., Schmedes A. (2017). Analysis of catecholamines in urine by unique LC/MS suitable ion-pairing chromatography. J. Chromatogr. B.

[61] Tohmola N., Itkonen O., Sane T., Markkanen H., Joenväärä S., Renkonen R., Hämäläinen E. (2014). Analytical and preanalytical validation of a new mass spectrometric serum 5-hydroxyindoleacetic acid assay as neuroendocrine tumor marker. Clin. Chim. Acta.

[62] Moriarty M., Lee A., O’Connell B., Kelleher A., Keeley H., Furey A. (2011). Development of an LC-MS/MS method for the analysis of serotonin and related compounds in urine and the identification of a potential biomarker for attention deficit hyperactivity/hyperkinetic disorder. Anal. Bioanal. Chem..

[63] Li X.S., Li S., Kellermann G. (2016). Pre-analytical and analytical validations and clinical applications of a miniaturized, simple and cost-effective solid phase extraction combined with LC-MS/MS for the simultaneous determination of catecholamines and metanephrines in spot urine samples. Talanta.

[64] Peitzsch M., Prejbisz A., Kroiss M., Beuschlein F., Arlt W., Januszewicz A., Siegert G., Eisenhofer G. (2013). Analysis of plasma 3-methoxytyramine, normetanephrine and metanephrine by ultraperformance liquid chromatography-tandem mass spectrometry: Utility for diagnosis of dopamine-producing metastatic phaeochromocytoma. Ann. Clin. Biochem..

[65] Li X., Li S., Wynveen P., Mork K., Kellermann G. (2014). Development and validation of a specific and sensitive LC-MS/MS method for quantification of urinary catecholamines and application in biological variation studies. Anal. Bioanal. Chem..

[66] Park N.-H., Hong J.Y., Shin H.J., Hong J. (2013). Comprehensive profiling analysis of bioamines and their acidic metabolites in human urine by gas chromatography/mass spectrometry combined with selective derivatization. J. Chromatogr. A.

[67] Shin H.J., Park N.H., Lee W., Choi M.H., Chung B.C., Hong J. (2017). Metabolic profiling of tyrosine, tryptophan and glutamate in human urine using gas chromatography–tandem mass spectrometry combined with single SPE cleanup. J. Chromatogr. B.

[68] Peitzsch M., Pelzel D., Glöckner S., Prejbisz A., Fassnacht M., Beuschlein F., Januszewicz A., Siegert G., Eisenhofer G. (2013). Simultaneous liquid chromatography tandem mass spectrometric determination of urinary free metanephrines and catecholamines, with comparisons of free and deconjugated metabolites. Clin. Chim. Acta.

[69] Konieczna L., Roszkowska A., Synakiewicz A., Stachowicz-Stencel T., Adamkiewicz-Drożyńska E., Bączek T. (2016). Analytical approach to determining human biogenic amines and their metabolites using eVol microextraction in packed syringe coupled to liquid chromatography mass spectrometry method with hydrophilic interaction chromatography column. Talanta.

[70] Oppolzer D., Moreno I., da Fonseca B., Passarinha L., Barroso M., Costa S., Queiroz J.A., Gallardo E. (2013). Analytical approach to determine biogenic amines in urine using microextraction in packed syringe and liquid chromatography coupled to electrochemical detection. Biomed. Chromatogr..

[71] Saracino M.A., Santarcangelo L., Raggi M.A., Mercolini L. (2015). Microextraction by packed sorbent (MEPS) to analyze catecholamines in innovative biological samples. J. Pharm. Biomed. Anal..

[72] Lindström M., Tohmola N., Renkonen R., Hämäläinen E., Schalin-Jäntti C., Itkonen O. (2018). Comparison of serum serotonin and serum 5-HIAA LC-MS/MS assays in the diagnosis of serotonin producing neuroendocrine neoplasms: A pilot study. Clin. Chim. Acta.

[73] Monteleone M., Naccarato A., Sindona G., Tagarelli A. (2013). A reliable and simple method for the assay of neuroendocrine tumor markers in human urine by solid-phase microextraction–gas chromatography-triple quadrupole mass spectrometry. Anal. Chim. Acta.

[74] Naccarato A., Gionfriddo E., Sindona G., Tagarelli A. (2014). Development of a simple and rapid solid phase microextraction-gas chromatography–triple quadrupole mass spectrometry method for the analysis of dopamine, serotonin and norepinephrine in human urine. Anal. Chim. Acta.

[75] Trifunovic-Macedoljan J., Pantelic N., Damjanovic A., Raskovic S., Nikolic-Djurovic M., Pudar G., Jadranin M., Juranic I., Juranic Z. (2016). LC/DAD determination of biogenic amines in serum of patients with diabetes mellitus, chronic urticaria or Hashimoto’s thyroiditis. J. Serbian Chem. Soc..

[76] Konieczna L., Pyszka M., Okońska M., Niedźwiecki M., Bączek T. (2018). Bioanalysis of underivatized amino acids in non-invasive exhaled breath condensate samples using liquid chromatography coupled with tandem mass spectrometry. J. Chromatogr. A.

[77] Clark Z.D., Cutler J.M., Pavlov I.Y., Strathmann F.G., Frank E.L. (2017). Simple dilute-and-shoot method for urinary vanillylmandelic acid and homovanillic acid by liquid chromatography tandem mass spectrometry. Clin. Chim. Acta.

[78] Wright M.J., Thomas R.L., Stanford P.E., Horvath A.R. (2015). Multiple Reaction Monitoring with Multistage Fragmentation (MRM3) Detection Enhances Selectivity for LC-MS/MS Analysis of Plasma Free Metanephrines. Clin. Chem..

[79] Dunand M., Donzelli M., Rickli A., Hysek C.M., Liechti M.E., Grouzmann E. (2014). Analytical interference of 4-hydroxy-3-methoxymethamphetamine with the measurement of plasma free normetanephrine by ultra-high pressure liquid chromatography–tandem mass spectrometry. Clin. Biochem..

[80] Matuszewski B.K., Constanzer M.L., Chavez-Eng C.M. (2003). Strategies for the Assessment of Matrix Effect in Quantitative Bioanalytical Methods Based on HPLC−MS/MS. Anal. Chem..

[81] Caban M., Migowska N., Stepnowski P., Kwiatkowski M., Kumirska J. (2012). Matrix effects and recovery calculations in analyses of pharmaceuticals based on the determination of β-blockers and β-agonists in environmental samples. J. Chromatogr. A.

[82] Pussard E., Neveux M., Guigueno N. (2009). Reference intervals for urinary catecholamines and metabolites from birth to adulthood. Clin. Biochem..

[83] Eisenhofer G., Lattke P., Herberg M., Siegert G., Qin N., Darr R., Hoyer J., Villringer A., Prejbisz A., Januszewicz A. (2013). Reference intervals for plasma free metanephrines with an age adjustment for normetanephrine for optimized laboratory testing of phaeochromocytoma. Ann. Clin. Biochem..

[84] Kelly A.U., Srivastava R., Dow E., Davidson D.F. (2017). L-DOPA therapy interferes with urine catecholamine analysis in children with suspected neuroblastoma: A case series. Ann. Clin. Biochem..

[85] Davidson D.F., Hammond P.J., Murphy D., Carachi R. (2011). Age-related medical decision limits for urinary free (unconjugated) metadrenalines, catecholamines and metabolites in random urine specimens from children. Ann. Clin. Biochem..

[86] Weise M., Merke D.P., Pacak K., Walther M.M., Eisenhofer G. (2002). Utility of Plasma Free Metanephrines for Detecting Childhood Pheochromocytoma. J. Clin. Endocrinol. MeTable.

[87] Pacak K., Eisenhofer G., Ahlman H., Bornstein S.R., Gimenez-Roqueplo A.-P., Grossman A.B., Kimura N., Mannelli M., McNicol A.M., Tischler A.S. (2007). Pheochromocytoma: Recommendations for clinical practice from the First International Symposium. Nat. Clin. Pract. Endocrinol. MeTable.

[88] Fitzgibbon M.C., Tormey W.P. (1994). Paediatric Reference Ranges for Urinary Catecholamines/Metabolites and Their Relevance in Neuroblastoma Diagnosis. Ann. Clin. Biochem..

[89] Claude B., Nehmé R., Morin P. (2011). Analysis of urinary neurotransmitters by capillary electrophoresis: Sensitivity enhancement using field-amplified sample injection and molecular imprinted polymer solid phase extraction. Anal. Chim. Acta.

[90] Kolobova E., Kartsova L., Kravchenko A., Bessonova E. (2018). Imidazolium ionic liquids as dynamic and covalent modifiers of electrophoretic systems for determination of catecholamines. Talanta.

[91] Li T., Wang Z., Xie H., Fu Z., He L., Ren J., Shi Z., Xu Z., Zhao Y., Zhao S. (2016). Separation of Key Biogenic Amines by Capillary Electrophoresis and Determination of Possible Indicators of Sport Fatigue in Athlete’s Urine. J. Chromatogr. Sci..

[92] Zhao Y., Zhao S., Huang J., Ye F. (2011). Quantum dot-enhanced chemiluminescence detection for simultaneous determination of dopamine and epinephrine by capillary electrophoresis. Talanta.

[93] Bacaloni A., Insogna S., Sancini A., Ciarrocca M., Sinibaldi F. (2013). Sensitive profiling of biogenic amines in human urine by capillary electrophoresis with field amplified sample injection. Biomed. Chromatogr..

[94] Li T., Wang Z., Xie H., Fu Z. (2012). Highly sensitive trivalent copper chelate-luminol chemiluminescence system for capillary electrophoresis detection of epinephrine in the urine of smoker. J. Chromatogr. B.

[95] Miekus N., Kowalski P., Oledzka I., Plenis A., Bień E., Miekus A., Krawczyk M., Adamkiewicz-Drozyńska E., Baczek T. (2015). Cyclodextrin-modified MEKC method for quantification of selected acidic metabolites of catecholamines in the presence of various biogenic amines. Application to diagnosis of neuroblastoma. J. Chromatogr. B Anal. Technol. Biomed. Life Sci..

[96] Miękus N., Olędzka I., Plenis A., Kowalski P., Bień E., Miękus A., Krawczyk M.A., Adamkiewicz-Drożyńska E., Bączek T. (2016). Determination of urinary biogenic amines’ biomarker profile in neuroblastoma and pheochromocytoma patients by MEKC method with preceding dispersive liquid–liquid microextraction. J. Chromatogr. B.

[97] Miękus N., Olędzka I., Kossakowska N., Plenis A., Kowalski P., Prahl A., Bączek T. (2018). Ionic liquids as signal amplifiers for the simultaneous extraction of several neurotransmitters determined by micellar electrokinetic chromatography. Talanta.

[98] Hsieh M.-M., Lin E.-P., Huang S.-W. (2012). On-line concentration and separation of cationic and anionic neurochemicals by capillary electrophoresis with UV absorption detection. Talanta.

[99] Zhang Y., Zhang Y., Wang G., Chen W., Li Y., Zhang Y., He P., Wang Q. (2016). Sensitive determination of neurotransmitters in urine by microchip electrophoresis with multiple-concentration approaches combining field-amplified and reversed-field stacking. J. Chromatogr. B.

[100] Xu X., Zhang H., Shi H., Ma C., Cong B., Kang W. (2012). Determination of three major catecholamines in human urine by capillary zone electrophoresis with chemiluminescence detection. Anal. Biochem..

[101] Kao Y.-Y., Liu K.-T., Huang M.-F., Chiu T.-C., Chang H.-T. (2010). Analysis of amino acids and biogenic amines in breast cancer cells by capillary electrophoresis using polymer solutions containing sodium dodecyl sulfate. J. Chromatogr. A.

[102] Tang W., Ge S., Gao F., Wang G., Wang Q., He P., Fang Y. (2013). On-line sample preconcentration technique based on a dynamic pH junction in CE-amperometric detection for the analysis of biogenic amines in urine. Electrophoresis.

[103] Zhao J., Zhang Q., Yang H., Tu Y. (2011). Electrophoretic separation of neurotransmitters on a polystyrene nano-sphere/polystyrene sulphonate coated poly(dimethylsiloxane) microchannel. Biomicrofluidics.

[104] Wang L., Liu Y., Xie H., Fu Z. (2012). Trivalent copper chelate-luminol chemiluminescence system for highly sensitive CE detection of dopamine in biological sample after clean-up using SPE. Electrophoresis.

[105] Zhou L., Glennon J.D., Luong J.H.T. (2010). Electrophoretic Analysis of Biomarkers using Capillary Modification with Gold Nanoparticles Embedded in a Polycation and Boron Doped Diamond Electrode. Anal. Chem..

[106] Xu X., Jia Z., Shu Y., Liu L. (2015). Dynamic pH junction–sweeping technique for on-line concentration of acidic amino acids in human serum by capillary electrophoresis with indirect UV detection. J. Chromatogr. B.

[107] Piešťanský J., Maráková K., Mikuš P. (2017). Two-Dimensional Capillary Electrophoresis with On-Line Sample Preparation and Cyclodextrin Separation Environment for Direct Determination of Serotonin in Human Urine. Molecules.

[108] Lepage R., Albert C. (2006). Fifty years of development in the endocrinology laboratory. Clin. Biochem..

[109] Unger N., Hinrichs J., Deutschbein T., Schmidt H., Walz M., Mann K., Petersenn S. (2012). Plasma and Urinary Metanephrines Determined by an Enzyme Immunoassay but not Serum Chromogranin A for the Diagnosis of Pheochromocytoma in Patients with Adrenal Mass. Exp. Clin. Endocrinol. Diabetes.

[110] Sarathi V., Pandit R., Patil V., Lia A., Bandgar T., Shah N. (2012). Performance of Plasma Fractionated Free Metanephrines by Enzyme Immunoassay in the Diagnosis of Pheochromocytoma and Paraganglioma in Children. Endocr. Pract..

[111] Wijaya C., Lee J., Husain S., Ho C., McIntyre R., Tam W., Ho R. (2018). Differentiating Medicated Patients Suffering from Major Depressive Disorder from Healthy Controls by Spot Urine Measurement of Monoamines and Steroid Hormones. Int. J. Environ. Res. Public Health.

[112] Palus M., Formanová P., Salát J., Žampachová E., Elsterová J., Růžek D. (2015). Analysis of serum levels of cytokines, chemokines, growth factors and monoamine neurotransmitters in patients with tick-borne encephalitis: Identification of novel inflammatory markers with implications for pathogenesis. J. Med. Virol..

[113] Abu-Samak M.S. (2015). Correlation of Elevated Serum Serotonin Levels with Regular Aerobic Exercise is Associated with Alterations in Monocyte Count and Hemoglobin Levels during Winter Season. J. Appl. Sci..

[114] Taieb J., Benattar C., Birr A.S., Lindenbaum A. (2002). Limitations of steroid determination by direct immunoassay. Clin. Chem..

[115] Weismann D., Peitzsch M., Raida A., Prejbisz A., Gosk M., Riester A., Willenberg H.S., Klemm R., Manz G., Deutschbein T. (2015). Measurements of plasma metanephrines by immunoassay versus liquid chromatography with tandem mass spectrometry for diagnosis of pheochromocytoma. Eur. J. Endocrinol..

[116] Wood W.G. (2008). Immunoassays & Co.: Past, present, future?—A review and outlook from personal experience and involvement over the past 35 years. Clin. Lab..

[117] Si B., Song E. (2018). Recent Advances in the Detection of Neurotransmitters. Chemosensors.

[118] del Pozo M., Mejías J., Hernández P., Quintana C. (2014). Cucurbit[8]uril-based electrochemical sensors as detectors in flow injection analysis. Application to dopamine determination in serum samples. Sens. Actuators B Chem..

[119] Wang F., Wu Y., Lu K., Ye B. (2013). A simple but highly sensitive and selective calixarene-based voltammetric sensor for serotonin. Electrochim. Acta.

[120] Shi P., Miao X., Yao H., Lin S., Wei B., Chen J., Lin X., Tang Y. (2013). Characterization of poly(5-hydroxytryptamine)-modified glassy carbon electrode and applications to sensing of norepinephrine and uric acid in preparations and human urines. Electrochim. Acta.

[121] Xu X., Lin Q., Liu A., Chen W., Weng X., Wang C., Lin X. (2010). Simultaneous Voltammetric Determination of Ascorbic Acid, Dopamine and Uric Acid Using Polybromothymol Blue Film-Modified Glassy Carbon Electrode. Chem. Pharm. Bull..

[122] Zhang Y., Lei W., Xu Y., Xia X., Hao Q. (2016). Simultaneous Detection of Dopamine and Uric Acid Using a Poly(l-lysine)/Graphene Oxide Modified Electrode. Nanomaterials.

[123] Mazloum-Ardakani M., Khoshroo A. (2014). High sensitive sensor based on functionalized carbon nanotube/ionic liquid nanocomposite for simultaneous determination of norepinephrine and serotonin. J. Electroanal. Chem..

[124] Raj M., Gupta P., Goyal R.N., Shim Y.-B. (2017). Graphene/conducting polymer nano-composite loaded screen printed carbon sensor for simultaneous determination of dopamine and 5-hydroxytryptamine. Sens. Actuators B Chem..

[125] Goyal R.N., Bishnoi S. (2011). Simultaneous determination of epinephrine and norepinephrine in human blood plasma and urine samples using nanotubes modified edge plane pyrolytic graphite electrode. Talanta.

[126] Zhao J., Yu Y., Weng B., Zhang W., Harris A.T., Minett A.I., Yue Z., Huang X.-F., Chen J. (2013). Sensitive and selective dopamine determination in human serum with inkjet printed Nafion/MWCNT chips. Electrochem. Commun..

[127] Thomas T., Mascarenhas R.J., Swamy B.E.K., Martis P., Mekhalif Z., Sherigara B.S. (2013). Multi-walled carbon nanotube/poly(glycine) modified carbon paste electrode for the determination of dopamine in biological fluids and pharmaceuticals. Colloids Surf. B Biointerfaces.

[128] Qian T., Yu C., Zhou X., Ma P., Wu S., Xu L., Shen J. (2014). Ultrasensitive dopamine sensor based on novel molecularly imprinted polypyrrole coated carbon nanotubes. Biosens. Bioelectron..

[129] Lavanya N., Sekar C. (2017). Electrochemical sensor for simultaneous determination of epinephrine and norepinephrine based on cetyltrimethylammonium bromide assisted SnO_2_ nanoparticles. J. Electroanal. Chem..

[130] Fayemi O.E., Adekunle A.S., Ebenso E.E. (2017). Electrochemical determination of serotonin in urine samples based on metal oxide nanoparticles/MWCNT on modified glassy carbon electrode. Sens. Bio-Sens. Res..

[131] Si B., Song E. (2018). Molecularly imprinted polymers for the selective detection of multi-analyte neurotransmitters. Microelectron. Eng..

[132] Wang X., You Z., Sha H., Cheng Y., Zhu H., Sun W. (2014). Sensitive electrochemical detection of dopamine with a DNA/graphene bi-layer modified carbon ionic liquid electrode. Talanta.

[133] Peaston R.T., Weinkove C. (2004). Measurement of catecholamines and their metabolites. Ann. Clin. Biochem..

[134] Franscini L.C., Vazquez-Montes M., Buclin T., Perera R., Dunand M., Grouzmann E., Beck-Popovic M. (2015). Pediatric reference intervals for plasma free and total metanephrines established with a parametric approach: Relevance to the diagnosis of neuroblastoma. Pediatr. Blood Cancer.

[135] Eisenhofer G., Goldstein D.S., Walther M.M., Friberg P., Lenders J.W.M., Keiser H.R., Pacak K. (2003). Biochemical Diagnosis of Pheochromocytoma: How to Distinguish True- from False-Positive Test Results. J. Clin. Endocrinol. MeTable.

[136] Bai T.R. (2009). Adrenergic Agonists and Antagonists. Allergy and Allergic Diseases.

[137] de Jong W.H.A., Post W.J., Kerstens M.N., de Vries E.G., Kema I.P. (2010). Elevated Urinary Free and Deconjugated Catecholamines after Consumption of a Catecholamine-Rich Diet. J. Clin. Endocrinol. MeTable.

[138] Devlin R.J., Henry J.A. (2008). Clinical review: Major consequences of illicit drug consumption. Crit. Care.

[139] Bannan L.T., Potter J.F., Beevers D.G., Saunders J.B., Walters J.R.F., Ingram M.C. (1984). Effect of alcohol withdrawal on blood pressure, plasma renin activity, aldosterone, cortisol and dopamine β-hydroxylase. Clin. Sci..

[140] Haass M., Kübler W. (1997). Nicotine and sympathetic neurotransmission. Cardiovasc. Drugs Ther..

[141] Davidson D.F. (2002). Phaeochromocytoma with normal urinary catecholamines: The potential value of urinary free metadrenalines. Ann. Clin. Biochem..

[142] Heron E. (1996). The Urinary Metanephrine-to-Creatinine Ratio for the Diagnosis of Pheochromocytoma. Ann. Intern. Med..

[143] Woltering E.A., Tellez M.R., Mamikunian G., Vinik A.I., O’Dorisio T.M. (2012). A Single Fasting Plasma 5-HIAA Value Correlates With 24-Hour Urinary 5-HIAA Values and Other Biomarkers in Midgut Neuroendocrine Tumors (NETs). Pancreas.

[144] Adaway J.E., Dobson R., Walsh J., Cuthbertson D.J., Monaghan P.J., Trainer P.J., Valle J.W., Keevil B.G. (2016). Serum and plasma 5-hydroxyindoleacetic acid as an alternative to 24-h urine 5-hydroxyindoleacetic acid measurement. Ann. Clin. Biochem..

[145] Tohmola N., Johansson A., Sane T., Renkonen R., Hamalainen E., Itkonen O. (2015). Transient elevation of serum 5-HIAA by dietary serotonin and distribution of 5-HIAA in serum protein fractions. Ann. Clin. Biochem..

[146] Dobson R., Burgess M.I., Banks M., Pritchard D.M., Vora J., Valle J.W., Wong C., Chadwick C., George K., Keevil B. (2013). The Association of a Panel of Biomarkers with the Presence and Severity of Carcinoid Heart Disease: A Cross-Sectional Study. PLoS ONE.

[147] Zhong X., Ye L., Su T.W., Xie J., Zhou W., Jiang Y., Jiang L., Ning G., Wang W. (2017). Establishment and evaluation of a novel biomarker-based nomogram for malignant phaeochromocytomas and paragangliomas. Clin. Endocrinol..

[148] Rifai N., Horvath A.R., Andrea R., Wittwer C., Tietz N.W. (2019). Tietz Fundamentals of Clinical Chemistry and Molecular Diagnostics.

[149] Barco S., Gennai I., Reggiardo G., Galleni B., Barbagallo L., Maffia A., Viscardi E., De Leonardis F., Cecinati V., Sorrentino S. (2014). Urinary homovanillic and vanillylmandelic acid in the diagnosis of neuroblastoma: Report from the Italian Cooperative Group for Neuroblastoma. Clin. Biochem..

[150] Verly I.R.N., van Kuilenburg A.B.P., Abeling N.G.G.M., Goorden S.M.I., Fiocco M., Vaz F.M., van Noesel M.M., Zwaan C.M., Kaspers G.L., Merks J.H.M. (2017). Catecholamines profiles at diagnosis: Increased diagnostic sensitivity and correlation with biological and clinical features in neuroblastoma patients. Eur. J. Cancer.

[151] Sandoval J.A., Malkas L.H., Hickey R.J. (2012). Clinical Significance of Serum Biomarkers in Pediatric Solid Mediastinal and Abdominal Tumors. Int. J. Mol. Sci..

[152] Nagatsu T., Nakashima A., Ichinose H., Kobayashi K. (2019). Human tyrosine hydroxylase in Parkinson’s disease and in related disorders. J. Neural Transm..

[153] Owens C., Irwin M. (2012). Neuroblastoma: The impact of biology and cooperation leading to personalized treatments. Crit. Rev. Clin. Lab. Sci..

[154] Gaetan G., Ouimet A., Lapierre C., Teira P., Sartelet H. (2014). Neuroblastoma presenting like a Wilms’ tumor with thrombus in inferior vena cava and pulmonary metastases: A case series. Springerplus.

[155] Dickson P.V., Sims T.L., Streck C.J., McCarville M.B., Santana V.M., McGregor L.M., Furman W.L., Davidoff A.M. (2008). Avoiding misdiagnosing neuroblastoma as Wilms tumor. J. Pediatr. Surg..

[156] Makrlíková A., Ktena E., Economou A., Fischer J., Navrátil T., Barek J., Vyskočil V. (2017). Voltammetric Determination of Tumor Biomarkers for Neuroblastoma (Homovanillic Acid, Vanillylmandelic Acid and 5-Hydroxyindole-3-acetic Acid) at Screen-printed Carbon Electrodes. Electroanalysis.

[157] Shen Y., Li H., Lu J., Luo X., Guan Q., Cheng L. (2019). Analytical validation and clinical application of urinary vanillylmandelic acid and homovanillic acid by LC-MS/MS for diagnosis of neuroblastoma. Biomed. Chromatogr..

[158] Hervet T., Grouzmann E., Grabherr S., Mangin P., Palmiere C. (2016). Determination of urinary catecholamines and metanephrines in cardiac deaths. Int. J. Legal Med..

[159] Pakanen L., Kortelainen M.-L., Särkioja T., Porvari K. (2011). Increased Adrenaline to Noradrenaline Ratio Is a Superior Indicator of Antemortem Hypothermia Compared with Separate Catecholamine Concentrations. J. Forensic Sci..

[160] Bessonova E., Kartsova L., Gallyamova V. (2017). Ionic liquids based on imidazole for online concentration of catecholamines in capillary electrophoresis. J. Sep. Sci..

[161] Fitzgerald P.A. (2011). Chapter 11. Adrenal Medulla and Paraganglia. Greenspan’s Basic & Clinical Endocrinology.

[162] Espay A.J., LeWitt P.A., Kaufmann H. (2014). Norepinephrine deficiency in Parkinson’s disease: The case for noradrenergic enhancement. Mov. Disord..

[163] Claußen M., Suter B. (2005). BicD-dependent localization processes: From Drosophilia development to human cell biology. Ann. Anat..

[164] Arias-Carrión Ó., Pöppel E. (2007). Dopamine, learning and reward-seeking behavior. Acta Neurobiol. Exp..

[165] Wiśniewski K., Car H. (2002). (S)-3,5-DHPG: A review. CNS Drug Rev..

[166] Roy A., Pickar D., De Jong J., Karoum F., Linnoila M. (1988). Norepinephrine and its metabolites in cerebrospinal fluid, plasma and urine. Relationship to hypothalamic-pituitary-adrenal axis function in depression. Arch. Gen. Psychiatry.

[167] Foti A., Adachi M., Dequattro V. (1982). The Relationships of Free to Conjugated Normetanephrine in Plasma and Spinal Fluid of Hypertensive Patients*. J. Clin. Endocrinol. MeTable.

[168] Eisenhofer G., Keiser H., Friberg P., Mezey E., Huynh T.-T., Hiremagalur B., Ellingson T., Duddempudi S., Eijsbouts A., Lenders J.W.M. (1998). Plasma Metanephrines Are Markers of Pheochromocytoma Produced by Catechol- O -Methyltransferase Within Tumors. J. Clin. Endocrinol. MeTable.

[169] Menon J.M.L., Nolten C., Achterberg E.J.M., Joosten R.N.J.M.A., Dematteis M., Feenstra M.G.P., Drinkenburg W.H., Leenaars C.H.C. (2019). Brain Microdialysate Monoamines in Relation to Circadian Rhythms, Sleep and Sleep Deprivation—A Systematic Review, Network Meta-analysis and New Primary Data. J. Circadian Rhythms.

[170] Perry M., Li Q., Kennedy R.T. (2009). Review of recent advances in analytical techniques for the determination of neurotransmitters. Anal. Chim. Acta.

[171] Iglesias S., Tomiello S., Schneebeli M., Stephan K.E. (2017). Models of neuromodulation for computational psychiatry. Wiley Interdiscip. Rev. Cogn. Sci..

[172] Cook E.H., Stein M.A., Krasowski M.D., Cox N.J., Olkon D.M., Kieffer J.E., Leventhal B.L. (1995). Association of attention-deficit disorder and the dopamine transporter gene. Am. J. Hum. Genet..

[173] Spivak B., Vered Y., Yoran-Hegesh R., Averbuch E., Mester R., Graf E., Weizman A. (1999). Circulatory levels of catecholamines, serotonin and lipids in attention deficit hyperactivity diiorder. Acta Psychiatr. Scand..

[174] Fahn S. (1979). The Biochemical Basis of Neuropharmacology. Neurology.

[175] Purves D., Augustine G.J., Fitzpatrick D., Hall W.C., LaMantia A.-S., McNamara J.O., White L.E. (2008). Neuroscience.

[176] Poovaiah N., Davoudi Z., Peng H., Schlichtmann B., Mallapragada S., Narasimhan B., Wang Q. (2018). Treatment of neurodegenerative disorders through the blood–brain barrier using nanocarriers. Nanoscale.

[177] Guimarães J., Vieira-Coelho M.A., Moura E., Afonso J., Rosas M.J., Vaz R., Garrett C. (2014). Urinary profile of catecholamines and metabolites in Parkinson patients with deep brain stimulation. Eur. J. Neurol..

[178] Klimek V., Stockmeier C., Overholser J., Meltzer H.Y., Kalka S., Dilley G., Ordway G.A. (1997). Reduced levels of norepinephrine transporters in the locus coeruleus in major depression. J. Neurosci..

[179] Corcuff J.-B., Chardon L., El Hajji Ridah I., Brossaud J. (2017). Urinary sampling for 5HIAA and metanephrines determination: Revisiting the recommendations. Endocr. Connect..

[180] Horikoshi S., Miura I., Kunii Y., Asano S., Kanno-Nozaki K., Mashiko H., Yabe H. (2017). Hashimoto encephalopathy with high plasma monoamine metabolite levels: A case report. Neuropsychiatr. Dis. Treat..

[181] Pilotto A., Blau N., Leks E., Schulte C., Deuschl C., Zipser C., Piel D., Freisinger P., Gramer G., Kölker S. (2019). Cerebrospinal fluid biogenic amines depletion and brain atrophy in adult patients with phenylketonuria. J. Inherit. Metab. Dis..

[182] Basnet B., Bhushan A., Khan R., Kumar G., Sharma V., Sharma A., Gupta S. (2018). Plasma & urinary catecholamines & urinary vanillylmandelic acid levels in patients with generalized vitiligo. Indian J. Med. Res..

[183] Tritsch N.X., Sabatini B.L. (2012). Dopaminergic Modulation of Synaptic Transmission in Cortex and Striatum. Neuron.

[184] Zhao Y.-Y. (2013). Metabolomics in chronic kidney disease. Clin. Chim. Acta.

[185] Zhao J. (2013). Plasma Kynurenic Acid/Tryptophan Ratio: A Sensitive and Reliable Biomarker for the Assessment of Renal Function. Ren. Fail..

